# Prehospital coagulation management and fluid replacement therapy in patients with multiple and/or severe injuries – A systematic review and clinical practice guideline update

**DOI:** 10.1007/s00068-025-02984-7

**Published:** 2025-11-14

**Authors:** Bjoern Hussmann, Peter Hilbert-Carius, Till Berk, Manuel Florian Struck, Erwin Strasser, Orkun Oezkurtul, Bjoern Hossfeld, Käthe Goossen, Charlotte M. Kugler, Marc Maegele

**Affiliations:** 1 Department of Trauma Surgery, Hochsauerland Hospital, Stolte Ley 5, 59759 Arnsberg, Germany; 2 Department of Orthopaedics and Trauma Surgery, Düsseldorf University Hospital, Moorenstraße 5, 40225 Düsseldorf, Germany; 3 Department of Anaesthesiology, Emergency and Intensive Care Therapy, Bergmannstrost BG Hospital Halle (Saale) gGmbH, Merseburgerstr. 165, 06112 Halle (Saale), Germany; 4 Department of Orthopedics, Trauma and Reconstructive Surgery, University Hospital RWTH Aachen, Pauwelsstraße 30, 52074 Aachen, Germany; 5 Manuel Florian Struck, Department of Anesthesiology and Intensive Care Medicine, University Hospital Leipzig, Liebigstr. 20, 04103 Leipzig, Germany; 6 Division of Transfusion Medicine, Cell Therapeutics and Haemostaseology, University Hospital, LMU Munich, Munich, Germany; 7 Orkun Oezkurtul, Department of Trauma and Reconstructive Surgery, BG Klinikum Bergmannstrost, 06112 Halle, Germany; 8 Department of Anesthesiology, Intensive Care Medicine, Emergency Medicine and Pain Therapy, Center of Emergency Medicine, Bjoern Hossfeld, HEMS “Christoph 22”, Armed Forces Hospital, Ulm, Germany; 9 Institute for Research in Operative Medicine (IFOM), Witten/Herdecke University, Cologne, Germany; 10 Department for Trauma and Orthopaedic Surgery, Cologne- Merheim Medical Center (CMMC), Institute for Research in Operative Medicine (IFOM), Witten/Herdecke University, Ostmerheimer Str. 200, 51109 Cologne, Germany

**Keywords:** Prehospital volume, Outcome, Coagulation therapy, Polytrauma guideline

## Abstract

**Purpose:**

Our aim was to update the evidence-based and consensus-based recommendations for prehospital coagulation management and fluid replacement therapy in patients with multiple and/or severe injuries on the basis of current evidence. This guideline topic is part of the 2022 update of the German Guideline on the Treatment of Patients with Multiple and/or Severe Injuries.

**Methods:**

MEDLINE and Embase were systematically searched to May 2021. Further literature reports were obtained from clinical experts. Randomised controlled trials, prospective cohort studies, and comparative registry studies were included if they compared interventions for fluid replacement therapy, the transfusion of blood products, the management of coagulation, or intravenous/intraosseous access in patients with multiple and/or severe injuries in the prehospital setting. We considered patient-relevant clinical outcomes such as mortality and bleeding control, or coagulation parameters as surrogate outcomes. Risk of bias was assessed using NICE 2012 checklists. The evidence was synthesised narratively, and expert consensus was used to develop recommendations and determine their strength.

**Results:**

Thirty-five new studies were identified. Interventions covered were prehospital fluid replacement therapy (*n* = 5 studies), infusions (*n* = 3), transfusions (*n* = 11), coagulation management (*n* = 13), and intraosseous access (*n* = 2). Four recommendations were modified, and six additional recommendations were developed. All achieved strong consensus.

**Conclusion:**

The following key recommendations are made. Fluid replacement therapy should be initiated in severely injured patients. In patients with uncontrolled bleeding, fluid replacement therapy should be limited (MAP of 65 mmHg, SBP of 80 mmHg) in order to maintain minimum haemodynamic stability while not increasing blood loss. In hypotensive patients with suspected isolated or concomitant significant traumatic brain injury, the objective of fluid replacement should be to maintain normal blood pressure (MAP of 85 mmHg, SBP of 110 mmHg). Intravenous access is used in trauma patients. If intravenous access cannot be achieved in trauma patients, intraosseous access is used for the delivery of fluids and medications. If there are no signs and symptoms of volume depletion, fluid replacement therapy should not be provided. If the administration of a sufficient volume of fluids fails to achieve adequate blood pressure in a polytrauma patient, the titrated use of vasopressors for circulatory support may be considered. The lethal triad of hypothermia, acidosis, and coagulopathy should be addressed at the prehospital stage. One gram of tranexamic acid should be administered in cases of existing or imminent haemorrhagic shock. The administration of fibrinogen may also be considered in cases of uncontrollable bleeding, as may the administration of erythrocyte and plasma concentrates.

**Supplementary Information:**

The online version contains supplementary material available at 10.1007/s00068-025-02984-7.

## Introduction

Hardly any other prehospital intervention has changed as profoundly in recent years and decades as coagulation and fluid replacement therapy. Whereas the administration of large amounts of fluids at the scene of injury after blunt trauma was propagated especially in the 1980 s and 1990 s, recent studies have shown that moderate volumes of fluids can improve overall outcome after blunt trauma [1–4]. This is in line with lessons learned from the treatment of patients with severe injuries after penetrating trauma. Since the 1990 s, if not earlier, it has been known that rapid transport to an appropriate hospital and restrictive fluid therapy (permissive hypotension) improve outcome [5].

Apart from prehospital fluid therapy, an increasingly important role is played by the coagulation system and coagulation disorders resulting from severe injuries that must be addressed and should be stabilised in the prehospital setting. Examples of this are the prehospital administration of tranexamic acid as well as the prehospital use of blood products and substances that influence coagulation. Against this background, advice and recommendations on how to optimise coagulation and on how to manage coagulation disorders in the prehospital setting are given below, with a focus on new findings in the fields of pathophysiology, diagnosis and treatment.

In this context, it should be noted that scientific investigations are difficult especially when it comes to prehospital treatments. One reason for this is that patient populations are highly heterogeneous. Large prospective studies with a high level of evidence are thus rare in recent literature.

Bleeding remains the most preventable cause of death worldwide [6]. The consequences of severe bleeding include not only the loss of oxygen carriers and reduced blood flow to organs, resulting in organ or multi-organ failure, but also a loss of clotting factors. Taken together, this leads to a further increase in mortality if targeted treatment, which should ideally be administered at the scene of the accident, is not provided. Our aim was to update the evidence-based and consensus-based recommendations for prehospital coagulation management and fluid replacement therapy in patients with multiple and/or severe injuries on the basis of current evidence. This guideline is therefore intended to be an important tool for solving the medical problem of “bleeding after severe trauma and coagulation failure” and to assist emergency medical personnel in their practical work.

## Methods

This guideline topic is part of the 2022 update of the German Guideline on the Treatment of Patients with Multiple and/or Severe Injuries [7]. The guideline update is reported according to the RIGHT tool [8], the systematic review part according to the Preferred Reporting Items for Systematic Reviews and Meta-Analyses (PRISMA) 2020 reporting guideline [9]. The development and updating of recommendations followed the standard methodology set out in the guideline development handbook issued by the German Association of the Scientific Medical Societies (AWMF) [10]. All methods were defined a priori, following the methods report of the previous guideline version from July 2016 [11] with minor modifications, as detailed below. Because this methods report was publicly available, the systematic review protocol was not published separately. This work is based on the corresponding chapter of the S3 guideline on polytrauma [7]. Publication as a systematic review has the advantage that, in contrast to the full guideline, the relevant parts of the method report, the guideline chapter and the evidence tables are directly related to each other so that the reader gets a clear overview of all these aspects in one work. This approach was chosen to increase the dissemination and improve implementation of the guideline content overall.

### PICO questions and eligibility criteria

Population, intervention, comparison, and outcome (PICO) questions were retained from the previous guideline version, and supplemented with questions on prehospital coagulation management which had previously been addressed in Sect. 2.16 (Coagulation Management) of the 2016 guideline. In addition, the participating professional societies involved in guideline development were asked to submit new PICO questions. The overarching PICO question for this topic area was:

*In adult patients (≥ 14 years) with known or suspected polytrauma and/or severe injuries*,* does a specific prehospital fluid replacement therapy*,* blood transfusion protocol*,* or approach to coagulation management improve patient-relevant outcomes compared to any other intervention?*

The full set of predefined PICO questions is listed in Table S1 (Online Resource 1). The study selection criteria in the PICO format are shown in Table 1.

**Table 1:** Predefined selection criteria

Population:	adult patients (≥ 14 years) with polytrauma and/or severe injuries^a, b^
Intervention/ comparison:	• prehospital fluid therapy or transfusion of blood products *or* • prehospital management of coagulation or severe bleeding *or* • prehospital intravenous/intraosseous access
Outcomes:	any patient-relevant clinical outcomes, such as mortality and bleeding control; if unavailable, coagulation parameters as surrogate outcomes
Study type:	• comparative, prospective studies (randomised controlled trials, cohort studies) • comparative registry^c^ data (incl. case-control studies) • systematic reviews based on the above primary study types
Language:	English or German
Other inclusion criteria:	• full text of study published and accessible • study matches predefined PICO question
Exclusion criteria:	• multiple publications of the same study without additional information • study already included in previous guideline version

^a^ Defined by an Injury Severity Score (ISS) > 15, Glasgow Coma Scale (GCS) < 9, or comparable values on other scales, or, in the prehospital setting, clinical suspicion of polytrauma/severe injury with a need for life-saving interventions

^b^ For new PICO questions, indirect evidence from other populations was eligible for inclusion if direct evidence was unavailable.

^c^ Using the Agency for Healthcare Research and Quality (AHRQ) definition of registries [12]

### Literature search

An information specialist systematically searched for literature in MEDLINE (Ovid) and Embase (Elsevier). The search strategy described in the 2016 guideline update was used with minor modifications. It contained index (MeSH/Emtree) and free text terms for the population and intervention. Additional terms were included for new PICO questions. For the topic area of coagulation management and fluid replacement therapy, a combined search was conducted covering both the prehospital and hospital settings. The searches were completed on 7 May 2021. No start date was used in the searches for new PICO questions. The start date for update searches was 1 January 2014. Table S2 (Online Resource 1) provides details for all searches. Studies referenced in the Methods section of included studies were also retrieved, and clinical experts were asked to submit additional relevant references.

### Study selection

Study selection was performed independently by two reviewers (K.G., C.K.) in a two-step process using the predefined eligibility criteria: (1) title/abstract screening of all references retrieved from database searches using Rayyan software [13] and (2) full-text screening of all articles deemed potentially relevant by at least one reviewer at the title/abstract level in Endnote (Endnote, Version: 20 [Software], Clarivate, Boston, Massachusetts, USA, https://endnote.com/). Studies limited to the hospital setting were excluded during full-text screening. Disagreements were resolved through consensus or by consulting a third reviewer (B.H.). The reasons for full-text exclusion were recorded (Table S3, Online Resource 1).

### Assessment of risk of bias and level of evidence

Two reviewers (K.G., C.K.) sequentially assessed the risk of bias of included studies at study level using the relevant checklists from the NICE guidelines manual 2012 [14] and assigned each study an initial level of evidence (LoE) using the Oxford Centre for Evidence-based Medicine Levels of Evidence (2009) [15]. For studies with baseline imbalance and unadjusted analyses, post-hoc secondary analyses, indirectness of the study population, or low power and imprecision of the effect estimate, the LoE was downgraded and marked with an arrow (↓). Any disagreements were resolved through consensus or by consulting a third reviewer.

### Data extraction and data items

Data were extracted into a standardised data table by one reviewer (K.G. or C.K.) and checked by another (C.K. or K.G.). A predefined data set was collected for each study, consisting of study characteristics (study type, aims, setting), patient selection criteria and baseline characteristics (age, gender, injury scores, other relevant variables), intervention and control group treatments (including important co-interventions), patient flow (number of patients included and analysed), matching/adjusting variables, and data on outcomes for any time point reported.

### Outcome measures

Outcomes were extracted as reported in the study publications. For prospective cohort studies and registry data, preference was given to data obtained after propensity-score matching or statistical adjustment for risk-modulating variables over unadjusted data.

### Synthesis of studies

Studies were grouped by interventions. An interdisciplinary expert group used their clinical experience to synthesise studies narratively by balancing beneficial and adverse effects extracted from the available evidence. Priority was given to reducing mortality, immediate complications, and long-term adverse effects. Clinical heterogeneity was explored by comparing inclusion criteria and patient characteristics at baseline as well as clinical differences in the interventions and co-interventions.

### Development and updating of recommendations

For each PICO question, the following updating options were available: (1) the recommendation of the preceding version remains valid and requires no changes (“confirmed”); (2) the recommendation requires modification (“modified”); (3) the recommendation is no longer valid or required and is deleted; (4) a new recommendation needs to be developed (“new”). An interdisciplinary expert group of clinicians with expertise in trauma and acute care reviewed the body of evidence, drafted recommendations based on the homogeneity of clinical characteristics and outcomes, the balance between benefits and harms as well as their clinical expertise, and proposed grades of recommendation (Table 2). In the absence of eligible evidence, good practice recommendations were made based on clinical experience, data from studies with a lower level of evidence, and expert consensus in cases where the Guideline Group felt a statement was required due to the importance of the topic. These were not graded, and instead labelled as good (clinical) practice points (GPP). For GPPs, the strength of a recommendation is presented in the wording shown in Table 2.

**Table 2:** Grading of recommendations

Symbol	Grade of recommendation	Description	Wording (examples)
⇑⇑	A	strong recommendation	“use …”, “do not use …”
⇑	B	recommendation	“should use …”, “should not use …”
⇔	0	open recommendation	“consider using …”, “… can be considered”

### Consensus process

The Guideline Group finalised the recommendations during a web-based, structured consensus conference on 14 June 2021 via Zoom (Zoom, Version: 5.x [Software], Zoom Video Communications, Inc., San José, California, USA, https://zoom.us). A neutral moderator facilitated the consensus conference. Voting members of the Guideline Group were delegates of all participating professional organisations, including clinicians, emergency medical services personnel and nurses, while guideline methodologists attended in a supporting role. Members with a moderate, thematically relevant conflict of interest abstained from voting on recommendations, members with a high, relevant conflict of interest were not permitted to vote or participate in the discussion. Attempts to recruit patient representatives were unsuccessful. A member of the expert group presented recommendations. Following discussion, the Guideline Group refined the wording of the recommendations and modified the grade of recommendation as needed. Agreement with both the wording and the grade of recommendation was assessed by anonymous online voting using the survey function of Zoom. Abstentions were subtracted from the denominator of the agreement rate. Consensus strength was classified as shown in Table 3.

**Table 3:** Classification of consensus strength

Description	Agreement rate
strong consensus	> 95% of participants
consensus	> 75 to 95% of participants
majority approval	> 50 to 75% of participants
no approval	< 50% of participants

Recommendations were accepted if they reached consensus or strong consensus. For consensus recommendations with ≤ 95% agreement, diverging views by members of the Guideline Group were detailed in the background texts. Recommendations with majority approval were returned to the expert group for revision and further discussion at a subsequent consensus conference. Recommendations without approval were considered rejected.

### External review

During a four-week consultation phase, the coordinator of the overall guideline (Dr Dan Bieler) submitted the recommendations and background texts to all participating professional organisations for review. The participating organisations selected their own external reviewers, typically either their guidelines officers, or members of their executive board. The authors of this guideline topic were not involved in this process. Comments were collected using a structured review form. The results were then assessed, discussed and incorporated into the text by the guideline coordinator with the relevant author group.

The guideline was adopted by the executive board of the German Trauma Society on 17 January 2023.

### Quality assurance

The guideline recommendations were reviewed for consistency between guideline topic areas by the steering group. Where necessary, changes were made in collaboration with the clinical leads for all topic areas concerned. The final guideline document was checked for errors by the guideline chair and methodologist.

## Results

**Fig. 1:**
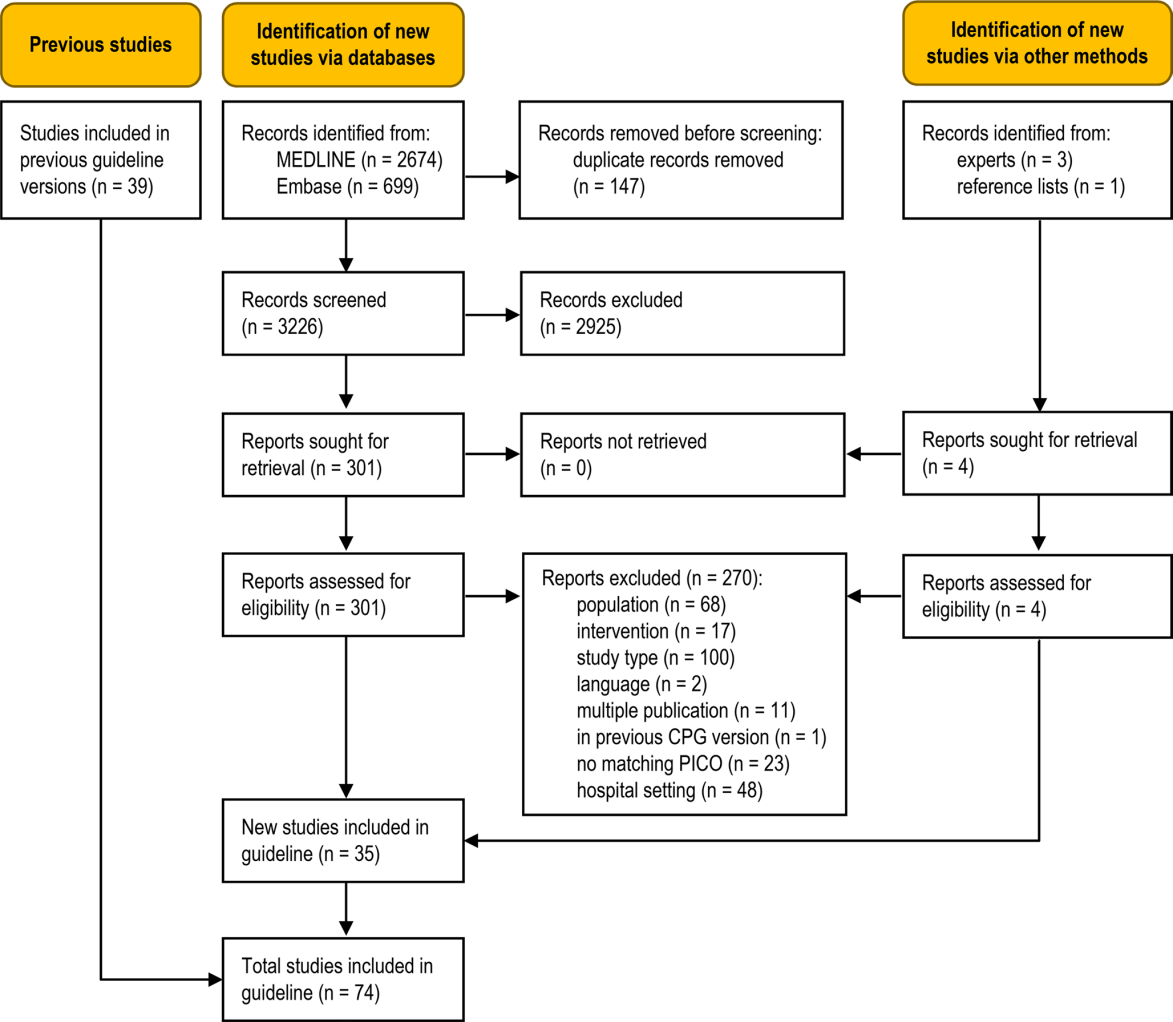
Modified PRISMA 2020 flow diagram showing the systematic literature search and selection of studies.

The database searches identified 3226 unique records (Fig. 1). Additional records were obtained from clinical experts, and from the reference list of an included study. Thirty-five new studies were eligible for this update [2, 4, 16–48], adding to the body of evidence from the 39 studies previously included in the guideline [5, 49–86]. A total of 271 full-text articles were excluded (Table S3, Online Resource 1).

### Characteristics of studies included in this update

Study characteristics, main outcomes, levels of evidence, and risk-of-bias assessments are presented in Table 4. Full details are provided in Table S4, Online Resource 1. This update included nine RCTs [16–24], eleven secondary analyses of RCT data [25–35], seven prospective cohort studies [36–42], and seven comparative registry studies [2, 4, 43–47], as well as one retrospective study with prospective data collection using audiovisual recordings [48]. Twenty studies were performed in North America, eight in Europe, two in Asia, and five were international multi-centre studies. Eligible patient populations were adults with severe injuries, mostly with severe bleeding or known/suspected haemorrhagic shock. Some studies were limited to subpopulations such as patients with traumatic brain injury [21, 26, 31, 44, 45], patients with abdominal trauma [43], or elderly patients [4].

**Table 4:** Characteristics of studies included in the update (see table S4, online resource 1 for details)

Study, ref., design	Population	Interventions (*N* patients)	Main outcomes (selection)^*^	LoE, risk of bias (RoB)^§^, comments
*Prehospital fluid therapy*				
Heuer 2015 [43] Registry study	136 severely injured patients^a^ with abdominal organ trauma	Prehospital fluid replacement: IG: 0–1000 mL (*N* = 68) CG: ≥1500 mL (*N* = 68)	**Matched cohort analysis** *Inhospital mortality*,* n (%)* 8 (11.8) vs. 13 (19.1) *24 h mortality*,* n (%)* 6 (8.8) vs. 12 (17.6)	LoE 2b Unclear RoB
Hussmann 2019 [44] Registry study	338 severely injured patients^a^ with severe TBI	Prehospital fluid replacement: IG: ≤1000 mL (*N* = 169) CG: >1500 mL (*N* = 169)	**Matched cohort analysis** *Inhospital mortality (%)* 45.6 vs. 45.6, *p* = 1.0	LoE 2b Unclear RoB
Hussmann 2015 [2] Registry study	7641 severely injured patients^a^	Prehospital fluid replacement: IG1: 0–500 mL (*N* = 1597) IG2: 501–1000 mL (*N* = 2047) IG3: 1001–1500 mL (*N* = 1530) IG4: 1501–2000 mL (*N* = 1161) IG5: ≥2001 mL (*N* = 1306)	**Multivariate regression** *Overall mortality*,* OR (95% CI)* IG1: reference IG2: 0.91 (0.73–1.14) IG3: 0.91 (0.71–1.12) IG4: 1.10 (0.79–1.35) IG5: 1.34 (1.02–1.73)	LoE 2b High risk of selection/performance bias Significant mortality benefit in patients *without* severe TBI
Leenen 2014 [4] Registry study	352 elderly (≥ 60 y) hypotensive^b^ patients with multiple trauma	Prehospital fluids (crystalloids plus colloids): IG: 0–1000 mL (*N* = 176) CG: >1000 mL (*N* = 176)	**Matched cohort analysis** *Mortality*,* n (%)* 52 (29.5) vs. 54 (30.7)	LoE 2b Unclear RoB Potential population overlap with Heuer 2015
Schreiber 2015 [16] RCT	292 hypotensive^c^ trauma patients	IG: controlled resuscitation (*N* = 97) CG: standard resuscitation (*N* = 95)	*24 h mortality*,* n/N (%)* 5/96 (5.2) vs. 14/95 (14.7) Adj. OR (95% CI): 0.39 (0.12–1.25)	LoE 2b↓ High risk of performance bias Underpowered for mortality endpoint (imprecision)
*Normotonic or hypertonic solutions*				
Delano 2015 [25] RCT	34 trauma patients in hypovolaemic shock^d^	Single prehospital bolus: IG1: 7.5% hypertonic saline (HTS) (*N* = 9) IG2: 7.5% HTS/6% Dextran 70 (*N* = 8) CG: 0.9% normal saline (*N* = 17)	*Base deficit*,* mEq/L*,* mean (SEM)* IG1: −9.2 (5.6) vs. CG: −9.4 (6.7) IG2: −13.1 (12.2) vs. CG: −9.4 (6.7) *Coagulopathy*,* n (%)* IG1: 5 (55.6) vs. CG: 8 (47.1) IG2 5 (62.5) vs. CG: 8 (47.1)	LoE 2b↓ Unclear RoB Surrogate outcomes only (indirectness), study possibly underpowered
Han 2015 [17] RCT	246 trauma patients in hypovolaemic shock^d^	IG1: 3% HTS (*N* = 82) IG2: 7.5% HTS (*N* = 80) CG: lactated Ringer’s solution (*N* = 84)	*24 h survival* “Better” in IG1 and IG2 than in CG, but difference n.s. *Coagulopathy*,* n (%)* IG1: 0 vs. CG: 9 (10.7), *p* < 0.001 IG2: 2 (2.5) vs. CG: 9 (10.7), *p* < 0.001	LoE 1b Unclear RoB Mortality endpoint not reported numerically
Rowell 2016 [36] Prospective cohort study	791 severely injured patients^e^ with and without TBI	*TBI subgroup (AIS*_*head*_ *≥3)* IG: lactated Ringer’s (*N* = 52) CG: normal saline (*N* = 256) *non-TBI subgroup (AIS*_*head*_ *<3)* IG: lactated Ringer’s (*N* = 65) CG: normal saline (*N* = 418)	Cox proportional hazards model * 30 d mortality*,* HR (95% CI)* *TBI patients*: 1.78 (1.04–3.04) *non-TBI patients*: 1.49 (0.757–2.95)	LoE 2b High risk of selection/performance bias Mortality adjusted for baseline imbalance (ISS)
*Prehospital transfusion of blood products*				
Brown 2015 [37] Prospective cohort study	113 blunt trauma patients in haemorrhagic shock	IG: prehospital RBC transfusion (*N* = 35)^f^ CG: no prehospital RBC transfusion (*N* = 78)^f^	Matched cohort analysis *24 h mortality*,* OR (95% CI)* 0.02 (0.01–0.69) * 30 d mortality*,* HR (95% CI)* 0.12 (0.03–0.61)	LoE 3b Unclear RoB Post-hoc analysis; pretrauma centre care not standardised
Gruen 2020 [26] RCT	166 trauma patients at risk for haemorrhagic shock; TBI subgroup	IG: thawed plasma (2 units followed by standard care) (*N* = 74) CG: standard care (crystalloid or crystalloid and packed RBCs) (*N* = 92)	* 30 d mortality*,* n (%)* 26 (35.1) vs. 51 (55.4), *p* = 0.01 *24 h mortality (n (%)* 12 (16.2) vs. 33 (35.9), *p* = 0.008	LoE 1b High risk of performance bias
Guyette 2021 [27] Secondary analysis of an RCT	407 trauma patients at risk for haemorrhagic shock	IG1: pRBCs + plasma (*N* = 38) IG2: plasma (*N* = 147) IG3: pRBCs only (*N* = 83) CG: crystalloids only (*N* = 139)	* 30 d mortality*,* n (%)* IG1: 10 (26) IG2: 31 (23) IG3: 30 (36) CG: 47 (37), (*p* = 0.05)	LoE 2b High risk of performance bias
Henriksen 2016 [38] Prospective cohort study	257 haemorrhaging trauma patients^g^	IG: prehospital plasma and/or RBCs (*N* = 75) CG: inhospital RBCs, plasma and platelets (*N* = 182)	*Unadj. 24 h mortality*,* n (%)* 12 (16) vs. 19 (10.4), *p* = 0.213 *Unadj. inhospital mortality*,* n (%)* 20 (26.7) vs. 38 (20.9), *p* = 0.313	LoE 3b↓ High risk of selection bias Mortality unadjusted for baseline imbalance
Holcomb 2017 [39] Prospective cohort study	109 high-risk patients with traumatic injuries (1058 before matching)	IG: prehospital transfusion (plasma and/or red blood cells) (*N* = 43 after matching) CG: crystalloid resuscitation (*N* = 66 after matching)	Matched cohort analysis *24 h mortality*,* adj. OR (95% CI)* 0.74 (0.25–2.17) * 30 d mortality*,* adj. OR (95% CI)* 0.85 (0.32–2.28)	LoE 3b↓ High risk of attrition bias Study probably underpowered (imprecision)
Holcomb 2015 [18] RCT	680 haemorrhaging patients with severe trauma	Transfusion of plasma, platelets and RBCs IG: ratio 1:1:1 (*N* = 338) CG: ratio 1:1:2 (*N* = 342)	*24-h mortality*,* adj. RR (95% CI)* 0.75 (0.52–1.08) *30-d mortality*,* adj. RR (95% CI)* 0.86 (0.65–1.12)	LoE 1b Unclear RoB
Moore 2018 [19] RCT	144 trauma patients in or at risk for haemorrhagic shock^d^	IG: two units of AB plasma (~ 250 mL each) in the prehospital setting (ITT *N* = 75; as treated *N* = 65) CG: standard of care (normal saline 0.9%, volume based on haemodynamic need) (ITT *N* = 69; as treated *N* = 60)	*24 h mortality*,* RR (95% CI)* 1.23 (0.45–3.34) (as treated) * 28 d mortality*,* RR (95% CI)* 1.54 (0.60–3.98) (as treated)	LoE 2b↓ High risk of performance bias Early termination of the study for futility (imprecision)
Pusateri 2020 [28] Two RCTs	626 trauma patients in or at risk for haemorrhagic shock^d^	IG: prehospital plasma (*N* = 297) CG: standard care (crystalloids) (*N* = 329)	By prehospital transport time *24 h mortality*,* adj. HR (95% CI)* *≤ 20 min*: 1.89 (0.65–5.40), *p* = 0.25 *> 20 min*: 0.53 (0.34–0.82), *p* = 0.004 * 28 d mortality*,* adj. HR (95% CI)* *≤ 20 min*: 1.71 (0.70–4.16), *p* = 0.24 *> 20 min*: 0.56 (0.40–0.80), *p* = 0.001	LoE 2b↓ High risk of performance bias Post-hoc subgroup analysis
Reitz 2020 [29] 2 RCTs	626 trauma patients in or at risk for haemorrhagic shock^d^	IG: prehospital plasma (N = n.r.) CG: standard care (crystalloids) (N = n.r.)	By mechanism of injury *24 h mortality*,* HR (95% CI)* *Blunt*: 0.59 (0.370–0.947) *Penetrating*: 1.16 (0.430–3.103) * 28 d mortality*,* HR (95% CI)* *Blunt*: 0.68 (0.472–0.965) *Penetrating*: 1.16 (0.430–3.103)	LoE 2b↓ High risk of performance bias Post-hoc subgroup analysis
Robinson 2018 [30] RCT	454 haemorrhaging patients with severe trauma; documented P/F ratio	Transfusion of plasma, platelets and RBCs IG: ratio 1:1:1 (*N* = 230) CG: ratio 1:1:2 (*N* = 224)	*ARDS*,* n (%)* 34 (14.8) vs. 41 (18.3) *p* = 0.35 * 30 d mortality*,* n (%)* 28 (12.3) vs. 28 (12.5), *p* = 0.91	LoE 2b↓ Unclear RoB Post-hoc analysis
Sperry 2018 [20] RCT	501 trauma patients in or at risk for haemorrhagic shock^d^	IG: prehospital administration of thawed plasma (group AB or group A) (*N* = 230) CG: standard-care resuscitation (crystalloids) (*N* = 271)	* 30 d mortality*,* n (%)* 53 (23.2) vs. 89 (33.0) Adj. OR (95% CI): 0.61 (0.40–0.91) *24 h mortality*,* n (%)* 32 (13.9) vs. 60 (22.1)	LoE 1b High risk of performance bias
*Tranexamic acid (TXA)*				
Bossers 2021 [45] Registry study	1827 patients with suspected severe TBI	IG: TXA (*N* = 693) CG: no TXA (*N* = 1134)	Adjusted survival analysis * 30 d mortality*,* OR (95% CI)* *Full cohort*: 1.18 (0.73–1.90) *Confirmed TBI*: 1.27 (0.68–2.35) *Isolated TBI*: 4.49 (1.57–12.87)	LoE 2b Unclear RoB
Brenner 2020 [31] RCT	7637 non-moribund^h^ TBI patients^i^ treated within 3 h of injury	IG: TXA CG: 0.9% sodium chloride (*N* = 7637 patients)	*24 h mortality*,* RR (95% CI)* 0.74 (0.58–0.94) * 28 d mortality*,* RR (95% CI)* 0.93 (0.83–1.03)	LoE 2b↓ Low RoB Post-hoc exploratory analysis
CRASH-3 collaborators 2019 [21] RCT	12,737 TBI patients^i^ treated within 3 h of injury	IG: TXA (*N* = 6406) CG: 0.9% sodium chloride (*N* = 6331)	*Head-injury-related 28 d mortality*,* RR (95% CI)* All patients: 0.94 (0.86–1.02) Non-moribund^h^: 0.89 (0.80–1.00.80.00) GCS score 9–15: 0.78 (0.64–0.95) GCS score 3–8: 0.99 (0.91–1.07)	LoE 1b Low RoB
Guyette 2020 [22] RCT	903 injured patients at risk for haemorrhage	IG1: TXA/PBO/PBO (*N* = 151) IG2: TXA/TXA/PBO (*N* = 141) IG3: TXA/TXA/TXA (*N* = 150) CG: PBO/PBO/PBO (*N* = 456)	* 30 d mortality*,* RR (95% CI)* 0.82 (0.60–1.11) By time from injury (post-hoc) * 30 d mortality*,* RR (95% CI)* *≤ 1 h*: 0.60 (0.44–0.83) *> 1 h*: 0.92 (0.52–1.64)	LoE 1b Low RoB
Khan 2018 [32] Secondary analysis of an RCT	93 trauma patients with hyperfibrinolysis^k^ (117 before matching)	IG: TXA (*N* = 31) CG: no TXA (*N* = 62)	Matched cohort analysis *24 h mortality*,* %* 26 vs. 39, *p* = 0.25 * 30 d mortality: %* 45 vs. 50, *p* = 0.82	LoE 2b Low RoB
Meizoso 2018 [40] Prospective cohort study	218 severely injured trauma patients	IG: TXA (*N* = 35) CG: no TXA (*N* = 183)	*Mortality*,* n (%)* 6 (17.1) vs. 27 (14.8), *p* = 0.718 *Fibrinolysis shutdown*,* n (%)* 32 (91.4) vs. 107 (58.5), *p* < 0.0001	LoE 3b↓ High risk of selection bias Outcomes unadjusted for baseline imbalance
Moore 2017 [41] Prospective cohort study	232 severely injured trauma patients	IG: TXA (*N* = 26) CG: no TXA (*N* = 206)	By fibrinolysis phenotype *Inhospital mortality*,* %*,* adj. p-value* *Hyperfibrinolysis*: 56 vs. 19, *p* = 0.116 *Fibrinolysis shutdown*: 38 vs. 28, *p* = 0.597 *Physiologic fibrinolysis*: 63 vs. 11, *p* = 0.018	LoE 2b High risk of selection/performance bias
Nishijima 2019 [33] RCT	13,432 trauma patients with or at risk of significant bleeding^m^	IG: TXA (*N* = 6753) CG: PBO (*N* = 6679)	*Functional status (modified Oxford Handicap Scale score)*,* mean ± SD* 0.66 ± 0.33 vs. 0.64 ± 0.34 MD (95% CI): 0.02 (0.01–0.03), *p* < 0.001	LoE 1b Low RoB
Roberts 2014 [34] RCT	20,211 trauma patients with or at risk of significant bleeding^m^	IG: TXA (*N* = 10,093) CG: PBO (*N* = 10,114)	By days since injury *Death due to bleeding*,* HR (95% CI)* Day 0: 0.83 (0.73, 0.93) Day 1: 0.91 (0.79, 1.04) Day 2: 0.96 (0.77, 1.19) Day 3: 1.01 (0.76, 1.34) Day 4: 0.96 (0.70, 1.36)	LoE 1b Low RoB Unclear for predefined analysis
Roberts 2017 [35] RCT	20,211 trauma patients with or at risk of significant bleeding^m^	IG: TXA (*N* = 10,093) CG: PBO (*N* = 10,114)	By time to TXA treatment *Death due to bleeding*,* RR (95% CI)* ≤ 1 h: 0.68 (0.57–0.82) > 1 to ≤ 3 h: 0.79 (0.64–0.97) > 3 h: 1.44 (1.12–1.84)	LoE 1b Low RoB Predefined subgroup analysis
Shiraishi 2017 [46] Registry study	500 severely injured patients^a^	IG: TXA within 3 h after injury (*N* = 250) CG: no TXA (*N* = 250)	Matched cohort analysis *28-day mortality*,* OR (95% CI)* 0.49 (0.29, 0.83)	LoE 2b Unclear RoB
Spinella 2020 [23] RCT	150 patients with severe traumatic bleeding^n^	IG1: 2 g of TXA (*N* = 49) IG2: 4 g of TXA (*N* = 50) CG: PBO (*N* = 50)	*28-day mortality n/N*,* (%)* CG: 6/49 (12.2) TXA 2 g: 5/44 (11.4) TXA 4 g: 4/48 (8.33), *p* = 0.8	LoE 2b↓ Low RoB Underpowered for mortality (imprecision)
Wafaisade 2016 [47] Registry study	512 trauma patients with critical injuries^o^	IG: prehospital TXA (*N* = 258) CG: no prehospital TXA (*N* = 258)	Matched cohort analysis *24 h mortality*,* n (%)* 15 (5.8) vs. 32 (12.4), *p* = 0.01 * 30 d mortality*,* n (%)* 36 (14.0) vs. 42 (16.3), *p* = 0.54	LoE 2b Unclear RoB
*Fibrinogen*				
Ziegler 2021 [24] RCT	53 trauma patients with major or occult bleeding	IG: fibrinogen concentrate (*N* = 37) CG: PBO (*N* = 30)	*MCF at admission*,* median difference (IQR)* *FIBTEM*: −4, (−7 to −2), *p* < 0.0026 *EXTEM*: −5 (−9 to −2), *p* = 0.0102	LoE 2b↓ High risk of attrition bias Surrogate outcomes (indirectness)
*Intraosseous access*				
Chreiman 2018 [48] Retrospective cohort study^p^	38 hypovolaemic trauma patients in extremis (145 vascular access attempts)	IG: intraosseous access (*N* = 52 attempts) CG1: peripheral IV access (*N* = 37) CG2: central venous catheter access (*N* = 52) CG3: intracardiac line (*N* = 4)	*Success rate*,* n/N (%)* IG 48/52 (92); CG1: 12/37 (43.2) CG2: 23/52 (44.2) CG3: 3/4 (75), *p* < 0.001	LoE 3b↓ High risk of selection bias Baseline comparability unclear
Leidel 2012 [42] Prospective cohort study	40 severely injured or critically ill patients under resuscitation	IG: intraosseous access (*N* = 40) CG: central venous catheter (*N* = 40)	*Success rate on 1 st attempt*,* % (95% CI) * 85 (74–96) vs. 60 (45–75), *p* = 0.024 *Procedure time [min]*,* median (IQR)* 2.0 (1.0–3.0) vs. 8.0 (5.5–10.0), *p* < 0.001	LoE 3b↓ High risk of performance bias Population incl. non-trauma patients (indirectness)

* Data for IG versus CG unless otherwise specified. ^§^ Risk of bias: low RoB=RoB low for all domains; unclear RoB=RoB unclear for at least one domain, no high RoB in any domain; for studies with high RoB, all domains with high RoB are named, with RoB low or unclear for all other domains (for full details Table S4, Online Resource 1). ↓ indicates that the level of evidence was lowered due to concerns related to the study design, as detailed in the Reviewers’ conclusion.^a^ Severely injured patients defined as patients with an ISS ≥16; ^b^ hypotensive defined as “systolic blood pressure at the accident site between 60 and 100 mmHg”; ^c^ hypotensive defined as “either a SBP <70 mmHg or an absent radial pulse”; ^d^ hypovolaemic shock defined as out-of-hospital systolic blood pressure (SBP) ≤70 mmHg or 71–90 mmHg with a heart rate ≥108 beats/min;^e^ severely injured patients defined as patients who required the highest trauma level activation; ^f^ at any time before the subject’s arrival at the study trauma centre; ^g^ defined as patients who received blood before arrival at ED or after hospital admittance within 6 h of ED arrival; ^h^ non-moribund patients: excluded were patients with a GCS score of 3 or bilateral unreactive pupils; ^i^ patients with TBI defined as adults with a GCS score ≤12 or intracranial bleeding; ^k^ defined as Ly30 ≥3% on thromboelastography on admission; ^m^ SBP <90 mmHg and/or heart rate >110 beats/min; ^n^ patients who sustained a traumatic injury that required them to receive at least one unit of red blood cells (RBCs) or required an emergent operation for possible bleeding control; ^o^ defined as preclinically assessed NACA IV (potentially life-threatening), NACA V (acute danger), or NACA VI (respiratory and/or cardiac arrest); ^p^ study included because “the use of audiovisual recordings allowed us to collect data in a fashion similar to prospective real-time data collection […].” For abbreviations and acronyms see list included.

### Risk-of-bias assessment for included studies and levels of evidence

The risk of bias was unclear for eleven studies that reported insufficient study details. Eight studies, all RCTs or secondary analyses of RCT data, were judged to be at low risk of bias in all domains. The risk of selection bias was high in six further studies, eleven were at high risk of performance bias, and two were at high risk of attrition bias.

The level of evidence was downgraded for fifteen studies. Reasons for downgrading were baseline imbalance and unadjusted analysis (three studies), post-hoc secondary analysis (five studies), low power and imprecision of the effect estimate (four studies), and indirectness for three studies that included patients with non-severe injuries or reported surrogate outcomes only.

### Recommendations

Four recommendations were modified. Six additional recommendations or good practice points were developed based on the updated evidence and expert consensus (Table 5). All achieved strong consensus. Four recommendations from the 2016 Guideline were not retained in the 2022 update (Table S5, Online Resource 1).

**Table 5:** List of recommendations with grade of recommendation and strength of consensus

No.	GoR	New evidence, consensus	Recommendation	Status 2022
*Key recommendations*				
1	B ⇑	– 100%	Fluid replacement therapy should be initiated in severely injured patients. In patients with uncontrolled bleeding, fluid replacement therapy should be limited (MAP of 65 mmHg, SBP of 80 mmHg) in order to maintain minimum haemodynamic stability while not increasing blood loss.	Modified
2	B ⇑	– 100%	In hypotensive patients with suspected isolated or concomitant significant traumatic brain injury, the objective of fluid replacement should be to maintain normal blood pressure (MAP of 85 mmHg, SBP of 110 mmHg).	Modified
3	A ⇑⇑	– 100%	Use intravenous access in trauma patients.	Confirmed
4	A ⇑⇑	[42, 48] 100%	If intravenous access cannot be achieved in trauma patients, use intraosseous access for the delivery of fluids and medications.	New
5	B ⇑	[2] 100%	If there are no signs and symptoms of volume depletion, fluid replacement therapy should not be provided.	Modified
6	GPP	– 100%	If the administration of a sufficient volume of fluids fails to achieve adequate blood pressure in a polytrauma patient, consider the titrated use of vasopressors for circulatory support.	New
*Solutions for fluid replacement therapy*				
7	A ⇑⇑	– 100%	Use balanced crystalloid, isotonic electrolyte solutions for fluid replacement in trauma patients. If possible, first warm solutions.	Modified
8	0 ⇔	– 100%	Consider using balanced solutions, i.e. Ringer’s acetate or malate, instead of Ringer’s lactate.	Confirmed
*Coagulation management*				
9	GPP	– 100%	Address the lethal triad of hypothermia, acidosis and coagulopathy in the prehospital setting through (a) the prevention of further cooling of the patient (target: normal body temperature), (2) the appropriate treatment of haemorrhagic shock (bleeding control, fluid replacement and coagulation therapy), and (3) adequate oxygenation and ventilation (if necessary, intubation according to intubation criteria).	New
10	B ⇑	[22, 32, 34, 35] 100%	In polytrauma patients with actual or impending haemorrhagic shock, a bolus of 1 g of tranexamic acid (TXA) should be administered rapidly over ten minutes.	New
11	0 ⇔	[24] 100%	In polytrauma patients with uncontrolled haemorrhage, consider the administration of fibrinogen after the administration of tranexamic acid.	New
12	0 ⇔	[20, 26–28, 37, 38] 100%	In polytrauma patients with uncontrolled haemorrhage, consider the administration of red blood cell and plasma concentrates (fresh frozen plasma concentrates or lyophilised plasma concentrates) if logistics permit and transport to the destination hospital is not delayed.	New

GoR, grade of recommendation; MAP, mean arterial pressure; SBP, systolic blood pressure; TXA, tranexamic acid.

## Discussion

### Rationale for recommendations

#### Fluid replacement therapy

Hypoperfusion resulting from haemorrhage and subsequent traumatic haemorrhagic shock lead to an imbalance between oxygen supply and demand in the tissue [3, 82]. This impairment of microcirculation is considered to be responsible for secondary damage after haemorrhagic shock. Accordingly, the objective of fluid replacement therapy should be to improve microcirculation and thus organ perfusion. Previously, expert opinion was that vigorous fluid therapy had a positive effect on the outcome of patients with acute bleeding [87, 88]. In the literature, there is a paucity of prospective randomised studies addressing fluid therapy and severe injuries in the prehospital setting. In a recent prospective randomised study from 2015, Schreiber et al. showed that a controlled prehospital resuscitation strategy (boluses of 250 cc of fluid) did not have any disadvantages compared to aggressive fluid therapy (2000 mL) and that both types of treatment resulted in equal outcomes [16].It must be noted, however, that the majority of patients who were investigated in the study by Schreiber et al. had an Injury Severity Score (ISS) < 15 and thus did not meet the definition of severely injured patients.

Four older randomised controlled studies did not confirm the rationale for aggressive fluid therapy in the prehospital phase of care [1, 5, 59, 82]. In a study by Turner et al. [82], patients were randomly allocated to one of two treatment protocols and either received or did not receive fluid therapy in the prehospital setting. A total of 1309 patients were included. There were no differences between the groups in mortality, morbidity and long-term outcome [82].

In a study from 2002, Dutton et al. [1] randomised 110 patients with haemorrhagic shock to one of two fluid resuscitation protocols: target SBP >100 mmHg or target SBP of 70 mmHg. They found no differences between the groups. There were four deaths in each group. A similar protocol was used by Morrison et al. [59], who too randomised patients to one of two protocols with different target mean arterial pressures.

Bickell et al. [5] reported a negative effect of fluid therapy on the survival of patients after bleeding. Their study, however, included only patients with penetrating torso injuries. In this selective group of patients, prehospital fluid resuscitation was associated with a higher mortality rate (38% versus 30%) and a higher rate of postoperative complications (30% versus 23%) than delayed fluid resuscitation. The authors concluded that fluid therapy should not be administered in the prehospital setting and that surgery should be initiated as soon as possible.

Wang et al. conducted a meta-analysis, which was mainly based on the four studies mentioned above. They too concluded that aggressive fluid therapy might be associated with higher mortality [65]. In another large meta-analysis, Curry et al. performed a literature search in order to assess the extent to which modern volume therapies improved mortality, the correction of coagulopathy, and transfusion requirements. The authors found that there had been little improvement [53].

Apart from the aforementioned controlled trials, a wide variety of publications confirm these conclusions [89–93]. Special emphasis, however, is always placed on the situation of uncontrolled intrathoracic or intra-abdominal bleeding. In these cases, surgery should be performed as soon as possible and should not be delayed by prehospital measures. The objective should be moderate fluid therapy, i.e. “controlled hypotension” and systolic blood pressure of about 90 mmHg [1, 3, 79]. Recent retrospective registry studies have shown that the prehospital administration of lower volumes of fluid (< 1500 mL) improved outcome [36]. In a study from 2015, multivariate regression analysis demonstrated that the prehospital administration of increasing volumes of fluid was an independent risk factor for increased mortality [2]. A positive effect of moderately increased fluid volumes was found only in patients with concomitant severe traumatic brain injury (TBI).

For this reason, there are studies advising against the use of restrictive fluid therapy in patients with cardiac injuries or TBI [3, 81, 94]. As already mentioned, recent studies have shown that moderate fluid therapy had a mortality advantage especially in patients with concomitant severe TBI [2]. A study from 2019, however, did not demonstrate an advantage of aggressive over restrictive fluid therapy in patients with severe TBI [44].

By contrast, some authors advocated aggressive fluid therapy but investigated other patient populations such as patients with extremity injuries without uncontrollable bleeding [88, 95, 96].

Most studies recommended the initiation of vigorous fluid therapy after hospital arrival and before surgery and in patients with controllable bleeding. Expert opinion is that patients should receive fluid volumes that allow a target haematocrit of 25–30% to be achieved [97, 98]. Controlled studies, however, are not available.

A study found that patients who were given prehospital fluids spent 12–13 min longer at the incident scene than did patients who were not given fluids [82]. Some authors consider this delay to be of minor relevance [46], other authors believe that this delay is an important factor with adverse effects on mortality [99, 100]. Based on the information presented here and the contradictions in the current literature, the optimal rescue time remains unclear. The extent to which a prolonged rescue time in the range of minutes leads to a poorer outcome also remains controversial. Ultimately, the reference to the “golden hour of shock” that has existed for years remains. The ATLS principle of “stop the bleeding” may also be an indication for rapid treatment. In conclusion, further studies are needed to determine whether, in addition to these indications, there is an optimal rescue time that is accurate to the minute.

Intravenous access must be established in every patient since it is a prerequisite for the administration of medications and/or fluids. If venous access cannot be obtained in severely injured patients, for example because of marked hypovolaemia and venous collapse, intraosseous access must be established if it is provided by the emergency services [42]. The use of catecholamines in the management of severely injured patients is a matter of controversy and is considered as a last resort when the administration of a sufficient volume of fluids fails to maintain adequate circulation [101, 102].

Controversy has persisted for years over the best choice of solution to use. Since most data were obtained from animal experiments or during surgical procedures, they have always been of limited value. Especially the use of colloid solutions has been a matter of intense debate. In 2013, the Federal Institute for Drugs and Medical Devices (Bundesinstitut für Arzneimittel und Medizinprodukte) published an information note [103] that placed major restrictions on the use of solutions containing hydroxyethyl starches (HES). As a result, HES lost its role in fluid replacement therapy.

Even before the Federal Institute provided its clear recommendation, evidence suggested that the use of crystalloid solutions had advantages for trauma patients. In 1989, Velanovich et al. found a 12.3% decrease in mortality when crystalloid fluids were used for resuscitation [84]. Choi et al. confirmed this result in 1999 and reported that crystalloid resuscitation was associated with a lower mortality in trauma patients [73]. A Cochrane analysis from 2008 did not find differences between colloid and crystalloid solutions in trauma patients [71, 72, 104]. When crystalloid fluids are used, Ringer’s lactate should be preferred over isotonic saline [75, 105–107]. Experimental studies reported the occurrence of dilutional acidosis following the infusion of large volumes of isotonic saline solution [107–109]. When lactate is added to a balanced electrolyte solution (Ringer’s lactate), lactate is metabolised to bicarbonate and water. As a result, a source of bicarbonate is provided and dilutional acidosis can be prevented. Recent experimental studies, however, demonstrated disadvantages of Ringer’s lactate. For example, Ringer’s lactate was reported to activate neutrophils and thus to cause an increase in lung damage [67, 110–112]. It also appeared to increase apoptosis of granulocytes [112]. This has not been confirmed in clinical studies. Plasma lactate is used as a laboratory parameter in the diagnosis of the severity of shock. Ringer’s lactate leads to an iatrogenic increase in the concentration of lactate in plasma and thus may interfere with diagnosis [113, 114]. Ringer’s malate or acetate can be used instead of Ringer’s lactate. In animal experiments, Ringer’s malate was found to be associated with a lower mortality rate. A problem of Ringer’s lactate is that it is slightly hypo-osmolar and may therefore worsen cerebral oedema after traumatic brain injury. By contrast, Ringer’s acetate and malate are completely iso-osmolar [113, 114]. This means that the use of Ringer’s lactate can apparently no longer be recommended.

#### Coagulation management

Since hypothermia ≤ 34 °C considerably influences platelet function and the activity of clotting factors [115], care must be taken to maintain normothermia [116].

Apart from severe TBI (40–50% of deaths after severe trauma), uncontrolled bleeding is one of the most frequent causes of death and accounts for approximately 20–40% of deaths. Concomitant coagulopathy considerably exacerbates haemorrhage [117, 118]. This additional coagulation disorder is often caused by the bleeding itself (consumptive coagulopathy) or is not uncommonly triggered by trauma-induced and non-trauma-induced changes. Coagulopathy in polytrauma patients (trauma-induced coagulopathy, TIC) has been known for several decades [119]. In the early phase of care, it is therefore essential to address coagulopathy in the prehospital setting and to stabilise coagulation since uncontrolled haemorrhage leads to a significant increase in the number of deaths during the first six to twelve hours after trauma, with a peak in the first one to two hours [120].

With the publication of the Clinical Randomisation of an Antifibrinolytic in Significant Haemorrhage 2 (CRASH-2) trial, the early use of tranexamic acid (TXA) has been firmly established in the management of severely injured patients. The CRASH-2 trial was a prospective, randomised, placebo-controlled trial of the effects of the early administration of TXA on death, vascular occlusive events, and blood transfusion in trauma patients [121]. An analysis of 10,060 patients who had been allocated to tranexamic acid (TXA group) and 10,067 patients who had been allocated to placebo (placebo group) showed that the endpoint “mortality” was reduced with TXA (14.5% versus 16.0%, *p* = 0.0035). The risk of death due to bleeding was reduced as well (4.9% versus 5.7%, *p* = 0.0077). This trial, however, did not document data on laboratory parameters, injury severity (e.g. Injury Severity Score), and on the administration of other coagulation-active products or substances.

The CRASH-3 trial, which was a prospective, randomised, placebo-controlled trial of TXA in patients with traumatic brain injury [21], was published in 2019. The authors reported that TXA significantly reduced mortality in patients with mild or moderate TBI but had no advantage in patients with severe TBI. Brenner et al. obtained similar results [31], whereas Bossers et al. found in a study from 2021 that the administration of tranexamic acid was associated with increased mortality in patients with severe TBI [45].

In a double-blind, placebo-controlled trial from 2020, Guyette et al. demonstrated a positive effect of tranexamic acid on mortality in patients with severe haemorrhagic shock (systolic blood pressure ≤ 70 mmHg) [22]. Compared with H the placebo group, 30-day mortality was significantly lower when tranexamic acid was administered. Other authors reported similar results [32, 46, 47]. The early administration of TXA had a positive effect on outcome in all cases. The early time point appears to be the decisive factor. Nishijima et al. were able to show that TXA given within three hours from injury was associated with better outcomes than placebo [33]. Similar results were obtained by other authors [35].

A secondary explorative analysis of data from the CRASH-2 trial revealed that a survival benefit was detected only if treatment with TXA was initiated within the first three hours after trauma. TXA administration within the first three hours also reduced the risk of death due to bleeding by 28%, whereas TXA treatment given after three hours seemed to increase mortality [34, 122].

Of all coagulation factors, fibrinogen appears to be the most vulnerable in patients with severe haemorrhage and is the first to reach critical levels [123]. Fibrinogen concentrations decrease in patients with trauma-induced coagulopathy not only as a result of hyperfibrinolysis but also because of an increase in fibrinogen breakdown, acidosis, a decrease in fibrinogen synthesis due to hypothermia, and loss/dilution [124, 125]. In a double-blind, prospective, randomised trial from 2021, Ziegler et al. showed that the early prehospital administration of fibrinogen improves blood clot initiation and clot stability [24].

Uncontrolled bleeding continues to be a relevant problem in the prehospital management of severely injured patients and is a main cause of negative effects on patient outcome. The options for addressing uncontrolled bleeding at the scene of injury and during patient transport are, however, limited. Surgical bleeding control is usually not indicated in the prehospital setting. For this reason, the prehospital administration of oxygen carriers and plasma has been receiving considerable interest in the scientific community. Many recent studies demonstrated a survival benefit of the prehospital administration of red blood cell and plasma concentrates in severely injured patients. Brown et al., for example, reported that patients who received prehospital red blood cells had improved 30-day survival [37]. Guyette et al., too, found that red blood cell concentrates had an advantage over crystalloid volumes [27].

The prehospital administration of plasma also appeared to improve outcome in recent studies. In a study that was published in the New England Journal of Medicine in 2018, Sperry et al. showed a significant survival benefit in patients who received plasma [20]. Patients in particular benefit from plasma resuscitation during prolonged rescue operations. Pusateri et al. reported that prehospital plasma was associated with a survival benefit when transport times were longer than 20 min [28]. Similar results were obtained by other authors [29]. In addition, Gruen et al. found that the prehospital administration of plasma had a positive effect in patients with severe TBI [26]. By contrast, Zhang et al. showed that the early transfusion of a low dose (5 mL/kg body weight) of fresh frozen plasma was associated with an increased incidence of delayed traumatic intracranial haematoma in patients with TBI [126].

Disadvantages of prehospital plasma administration were reported, for example, by Moore et al., whose findings did not indicate that plasma improved outcomes or had positive effects [19]. The use of plasma even tended to be associated with higher mortality and a higher incidence of multiple organ failure.

In summary, the recent literature suggests a positive effect of the prehospital administration of red blood cell concentrates and plasma on the outcome of severely injured patients. Currently, however, these resources are not generally available in all types of ambulances. This would require that logistical challenges be successfully addressed. As a rule, a delay in transportation to the target hospital resulting from the administration of red blood cell concentrates and/or plasma must be prevented because uncontrolled haemorrhage requires surgical bleeding control or embolisation in the majority of cases. Unfortunately, the studies listed here do not explicitly examine whether the administration of coagulants prolongs rescue time. Further prospective randomized studies are therefore urgently needed and should be the subject of further investigation.

### Limitations of the guideline

Patient values and preferences were sought but not received. The effect of this on the guideline is unclear, and there is a lack of research evidence on the effect of patient participation on treatment decisions or outcomes in the emergency setting.

## Supplementary Information

Below is the link to the electronic supplementary material.

Supplementary Material 1 (PDF. 1.92 MB)

## Data Availability

A full version of the guideline and its methods/evidence report are available online at https://register.awmf.org/de/leitlinien/detail/187-023.

## Abbreviations

adj.: adjusted
ARDS: acute respiratory distress syndrome
CG: control group
CI: confidence interval
CRASH: Clinical Randomisation of an Antifibrinolytic in Significant Haemorrhage
d: days
ED: emergency department
GCS: Glasgow Coma Scale
GoR: grade of recommendation
GPP: good (clinical) practice point
h: hours
HR: hazard ratio
IG: intervention group
IQR: interquartile range
ISS: Injury Severity Score
LoE: level of evidence
MAP: mean arterial pressure
MCF: maximum clot firmness
MD: mean difference
NACA: National Advisory Committee for Aeronautics
n.r.: not reported
n.s.: not significant
OR: odds ratio
PBO: placebo
P/F ratio: ratio of arterial oxygen partial pressure (PaO2) to fractional inspired oxygen (FiO2)
RBC: red blood cell
RCT: randomised controlled trial
RoB: risk of bias
RR: risk ratio
SBP: systolic blood pressure
SD: standard deviation
TBI: traumatic brain injury
TXA: tranexamic acid
unadj.: unadjusted
y: years
