# Supplementary material for: Prehospital coagulation management and fluid replacement therapy in patients with multiple and/or severe injuries – A systematic review and clinical practice guideline update
Source: Eur J Trauma Emerg Surg. 2025 Nov 14;51(1):328. doi: 10.1007/s00068-025-02984-7 (PMC12618399; doi:10.1007/s00068-025-02984-7)
Supplement: Supplementary file 1 — Supplementary Material 1 (PDF. 1.92 MB) [file 68_2025_2984_MOESM1_ESM.pdf]

Online Resource

Prehospital coagulation management and fluid replacement therapy in patients with multiple and/or severe injuries – A systematic review and clinical practice guideline

Table S1. PICO questions[1]

The target population of the guideline consists of adults (≥14 years) with suspected polytrauma or trauma-related severe injury (ISS ≥16 and/or GCS <9) in the prehospital phase of care.

	Population	Intervention(s)	Control(s)	Outcome(s)
	**Volume therapy**			
1	Target population	prehospital volume therapy	No prehospital volume therapy	Bleeding, other clinically relevant outcomes
2	Target population, with uncontrollable bleeding	prehospital volume therapy in reduced form (blood pressure target low-stable)	prehospital volume therapy in non-reduced form (blood pressure target normotension)	Bleeding, other clinically relevant outcomes
3	Target population, hypotensive patients with traumatic brain injury	Volume therapy with normotension as blood pressure target	Reduced volume therapy with blood pressure target hypotension	clinically relevant outcomes
4	Target population, without evidence of volume depletion	No prehospital volume therapy	prehospital volume therapy	clinically relevant outcomes
	**Infusions**			
5	Target population, with volume deficiency	Crystalloid	open	clinically relevant outcomes
6	Target population, with volume deficiency	isotonic saline solution	open	clinically relevant outcomes
7	Target population, with volume deficiency	balanced, isotonic whole electrolyte solutions	open	clinically relevant outcomes

[1] Table translated from German using DeepL Translate (https://www.deepl.com/translator) with manual modifications

	Population	Intervention(s)	Control(s)	Outcome(s)
8	Target population, with volume deficiency	balanced, isotonic whole electrolyte solutions with acetate or malate	balanced, isotonic whole electrolyte solutions with lactate	clinically relevant outcomes
9	Target population, with volume deficiency	volume therapy with solutions containing human albumin	open	clinically relevant outcomes
	**Hypertonic solutions**			
10	Target population, after blunt trauma with hypotonic blood circulation	hypertonic solutions	open	clinically relevant outcomes
11	Target population, with penetrating trauma and volume deficiency	hypertonic solutions	open	clinically relevant outcomes
12	Target population, hypotonic, with severe traumatic brain injury	hypertonic solutions	open	clinically relevant outcomes
	**Accesses**			
13	Target population	venous access	no venous access	clinically relevant outcomes
14§	Target population	central venous catheters (subclavian, femoral or jugular)	peripheral venous catheters	clinically relevant outcomes
15§	Target population	arterial catheters	venous catheters	clinically relevant outcomes
16§	Target population	intraosseous access	other types of access (peripheral venous, central venous, arterial)	clinically relevant outcomes
17§	Target population receiving access to the blood stream	Placement of access with ultrasound diagnostics	Placement of access without ultrasound diagnosis	clinically relevant outcomes
	**Tranexamic acid**			
18§	Target population, massive bleeding	Tranexamic acid (prehospital)	no tranexamic acid (preclinical); other intervention	clinically relevant outcomes
	**Blood products**			
19§	Target population	transfusion with whole blood or blood components	open	clinically relevant outcomes

	Population	Intervention(s)	Control(s)	Outcome(s)
20[§]	Target population	Fibrinogen (prehospital)	No fibrinogen (prehospital); other intervention	Clinically relevant outcomes

[§] new PICO question

Table S2. Literature search details

Search strategy 2021, MEDLINE (via Ovid)	Date: 07.05.2021	2.545 Hits

1. exp Multiple Trauma/
2. (polytrauma* or trauma patient?).ti,ab,kf. or (severe adj2 shock).ti,ab,kf.
3. ((multiple or major or severe* or serious*) adj3 (trauma* or injur*)).ti,ab,kf.
4. ((blunt or penetrating) adj5 (trauma* or injur*)).ti,ab,kf.
5. (*Critical Care/ or *Emergencies/ or (life threatening or critical care or emergen*).ti,ab,kf.) and (trauma* or injur*).ti,ab,kf.
6. 1 or 2 or 3 or 4 or 5
7. exp animals/ not humans.sh.
8. (comment or editorial or letter).pt. or case report*.mp.
9. Shock, Hemorrhagic/ or exp Exsanguination/ or exp Hypovolemia/ or exp Shock, Traumatic/
10. (((uncontrolled or uncontrollable or acute or active or massive or life threatening or severe) adj2 (bleeding or h?emorrhage)) or ((hypovol?emic or h?emorrhagic or traumatic) adj (shock or trauma*)) or "damage control resuscitation").ti,ab,kf.
11. exp Hemostasis/ or exp Blood Coagulation/ or exp disseminated intravascular coagulation/ or exp Hemorrhage/
12. (h?emosta* or coagula* or clotting or coagulopath* or h?emorrhag* or bleed*).ti,ab,kf.
13. 9 or 10 or 11 or 12
14. exp Fluid Therapy/ or exp rehydration solutions/ or exp hypotonic solutions/ or exp isotonic solutions/ or exp crystalloid solutions/ or exp Colloids/ or exp ringer's lactate/ or exp saline solution/ or exp ringer's solution/ or exp Hydroxyethyl Starch Derivatives/ or Hypertonic Solutions/ or exp Saline Solution, Hypertonic/ or exp Plasma/ or exp Erythrocytes/ or exp Blood Transfusion/ or prothrombin complex concentrates.mp. or exp Deamino Arginine Vasopressin/ or exp Factor VIII/ or exp Platelet Transfusion/ or exp Fibrinogen/ or exp Tranexamic Acid/ or exp Blood Coagulation Tests/ or exp Thrombelastography/
15. (((fluid or volume) adj2 (therap* or replacement? or expansion or management or substitute or substitution or administration or resuscitation)) or "hypotensive resuscitation" or "permissive hypotension").ti,ab,kf. or (((isotonic or normal) adj (saline or sodium chloride or NACL)) or ((hypotonic or isotonic or buffered or balanced) adj (infusion? or solution? or electrolyte?)) or crystalloid? or colloid? or ringer* or albumin or hydroxyethyl starch* or hetastarch or dextran).ti,ab,kf. or ((hypertonic or hyperosmotic or hyperoncotic or hyperosmolar) adj2 (infusion? or solution? or electrolyte? or saline or resuscitation)).ti,ab,kf. or (((lactic acid or lactate) and shock) or (base adj (excess or deficit)) or acidosis or acid-base-status).ti,ab,kf. or (normotherm* or hypotherm* or ((preservation or retention or conservation or control or management) adj1 (temperature or thermic* or heat or warm*))).ti,ab,kf. or acid?emia.ti,ab,kf. or (hypocalc?emia or (reduced adj2 calcium)).ti,ab,kf. or (plasma or PRBC or PRBCs or transfusion or "factor concentrate?" or fibrinogen or hypofibrinogen?emia or factor xiia or factor viia or rfviia or thrombin).ti,ab,kf. or (tranexamic acid or txa).ti,ab,kf. or (thrombo* adj1 prophyla*).ti,ab,kf. or (blood gas analysis or quick or partial thromboplastin time or aptt or ((platelet or thrombocyte) adj count) or viscoelastic test? or thromb?elasto* or ROTEM or (rotation* and thromb?elastometry)).ti,ab,kf.
16. 14 or 15
17. 13 and 6 and 16
18. 17 not 7
19. 18 not 8
20. limit 19 to dt=20140101-20210322

Search strategy 2021, Additional question on intraosseous access, MEDLINE (via Ovid)
Date: 07.05.2021 83 Hits

1. exp Multiple Trauma/
2. (polytrauma* or trauma patient?).ti,ab,kf. or (severe adj2 shock).ti,ab,kf.
3. ((multiple or major or severe* or serious*) adj3 (trauma* or injur*)).ti,ab,kf.
4. ((blunt or penetrating) adj5 (trauma* or injur*)).ti,ab,kf.
5. (*Critical Care/ or *Emergencies/ or (life threatening or critical care or emergen*).ti,ab,kf.) and (trauma* or injur*).ti,ab,kf.
6. 1 or 2 or 3 or 4 or 5
7. exp animals/ not humans.sh.
8. (comment or editorial or letter).pt. or case report*.mp.
9. exp Infusions, Intraosseous/
10. ((intraosseous adj2 (infusion? or puncture? or access or device?))).ti,ab,kf.
11. 9 or 10
12. 6 and 11
13. 12 not 7
14. 13 not 8
15. limit 13 to dt=19460101-20210322

Search strategy 2021, Additional questions on access/catheterisation, MEDLINE (via Ovid)
Date: 07.05.2021 46 Hits

1. exp Multiple Trauma/
2. (polytrauma* or trauma patient?).ti,ab,kf. or (severe adj2 shock).ti,ab,kf.
3. ((multiple or major or severe* or serious*) adj3 (trauma* or injur*)).ti,ab,kf.
4. ((blunt or penetrating) adj5 (trauma* or injur*)).ti,ab,kf.
5. (*Critical Care/ or *Emergencies/ or (life threatening or critical care or emergen*).ti,ab,kf.) and (trauma* or injur*).ti,ab,kf.
6. 1 or 2 or 3 or 4 or 5
7. exp animals/ not humans.sh.
8. (comment or editorial or letter).pt. or case report*.mp.
9. Shock, Hemorrhagic/ or exp Exsanguination/ or exp Hypovolemia/ or exp Shock, Traumatic/
10. (((uncontrolled or uncontrollable or acute or active or massive or life threatening or severe) adj2 (bleeding or h?emorrhage)) or ((hypovol?emic or h?emorrhagic or traumatic) adj (shock or trauma*)) or "damage control resuscitation").ti,ab,kf.
11. exp Hemostasis/ or exp Blood Coagulation/ or exp disseminated intravascular coagulation/ or exp Hemorrhage/
12. (h?emosta* or coagula* or clotting or coagulopath* or h?emorrhag* or bleed*).ti,ab,kf.
13. 9 or 10 or 11 or 12
14. exp Catheterization, Central Venous/
15. (((heat exchang* or thermoregulatory or warming or hypothermia) adj4 catheter) or ((endovascular or intravascular) adj cooling)).ti,ab,kf. or (((central venous or arterial) adj catheter*) and (subclavian or femoral* or jugular*)).ti,ab,kf. or ((intravascular or vascular or intravenous or venous or iv or vein or arterial) adj (access* or cannulation)).ti,ab,kf. and (exp Ultrasonography/ or (ultrasonography* or ultrasound).ti,ab,kf.)

16 14 or 15

17. 13 and 6 and 16
18. 17 not 7

19. 18 not 8

20. limit 19 to dt=19460101-20210322

**Search strategy 2021, Embase (via Elsevier)**	**Date: 07.05.2021**	**667 Hits**

#1 'multiple trauma'/exp

#2 (polytrauma* OR "trauma patient?"):ti,ab,kw OR (severe NEXT/2 shock):ti,ab,kw

#3 ((multiple OR major OR severe* OR serious*) NEXT/3 (trauma* OR injur*)):ti,ab,kw

#4 ((blunt OR penetrating) NEXT/5 (trauma* OR injur*)):ti,ab,kw

#5 ('intensive care'/mj OR 'emergency'/mj OR ("life threatening" OR "critical care" OR emergen*):ti,ab,kw) AND (trauma* OR injur*):ti,ab,kw

#6 #1 OR #2 OR #3 OR #4 #5

#7 'animals'/exp NOT 'humans'/de

#8 (comment OR editorial OR letter):it OR "case report*":ti,ab,kw

#9 [1-1-2014]/sd NOT [23-3-2021]/sd

#10 [embase]/lim

#11 embase NOT (embase AND medline)

#12 'hemorrhagic shock'/exp OR 'hypovolemic shock'/exp OR 'traumatic shock'/exp OR 'exsanguination'/exp OR 'hypovolemia'/exp

#13 (((uncontrolled OR uncontrollable OR acute OR active OR massive OR "life threatening" OR severe) NEAR/2 (bleeding OR h$emorrhage)) OR ((hypovol$emic OR h$emorrhagic OR traumatic) NEXT/1 (shock OR trauma*)) OR "damage control resuscitation"):ti,ab,kw

#14 'hemostasis'/exp OR 'blood clotting'/exp OR 'disseminated intravascular clotting'/exp OR 'bleeding'/exp

#15 (h$emosta* OR coagula* OR clotting OR coagulopath*OR h$emorrhag* OR bleed*):ti,ab,kw

#16 #12 OR #13 OR #14 OR #15

#17 'fluid therapy'/exp OR 'rehydration'/exp OR 'hypotonic solution'/exp OR 'isotonic solution'/exp OR 'crystalloid'/exp OR 'colloid'/exp OR 'Ringer lactate solution'/exp OR 'sodium chloride'/exp OR 'Ringer solution'/exp OR 'hetastarch'/exp OR 'hypertonic solution'/exp OR 'sodium chloride'/exp OR 'blood clotting test'/exp OR 'thromboelastography'/exp OR 'plasma'/exp OR 'erythrocyte'/exp OR 'blood transfusion'/exp OR 'prothrombin complex'/exp OR 'argipressin[1 deamino]'/exp OR 'blood clotting factor 8'/exp OR 'thrombocyte transfusion'/exp OR 'fibrinogen'/exp OR 'tranexamic acid'/exp

#18 (((fluid OR volume) NEAR/2 (therap* OR replacement? OR expansion OR management OR substitute OR substitution OR administration OR resuscitation)) OR "hypotensive resuscitation" OR "permissive hypotension"):ti,ab,kw OR (((isotonic OR normal) NEAR/1 (saline OR “sodium chloride” OR NACL)) OR ((hypotonic OR isotonic OR buffered OR balanced) NEAR/1 (infusion? OR solution? OR electrolyte?)) OR crystalloid? OR colloid? OR ringer* OR albumin OR “hydroxyethyl starch*” OR hetastarch OR dextran):ti,ab,kw OR ((hypertonic OR hyperosmotic OR hyperoncotic OR hyperosmolar) NEAR/2 (infusion? OR solution? OR electrolyte? OR saline OR resuscitation)):ti,ab,kw OR (“blood gas analysis” OR quick OR “partial thromboplastin time” OR aptt OR ((platelet OR thrombocyte) NEXT/1 count) OR “viscoelastic test?” OR thromb$elasto* OR ROTEM OR (rotation* AND thromb$elastometry)):ti,ab,kw OR (((“lactic acid” OR lactate) AND shock) OR (base NEXT/1 (excess or deficit)) OR acidosis OR acid-base-status):ti,ab,kw OR (normotherm* OR hypotherm* OR ((preservation OR retention OR conservation OR control OR management) NEAR/1 (temperature OR thermic* OR heat OR warm*))):ti,ab,kw OR acid$emia:ti,ab,kw OR (hypocalc$emia OR (reduced NEAR/2 calcium)):ti,ab,kw OR (plasma OR PRBC OR PRBCs OR transfusion OR "factor concentrate?" OR fibrinogen OR hypofibrinogen$emia OR “factor xiia” OR “factor viia” OR rfviia OR thrombin):ti,ab,kw OR (“tranexamic acid” OR txa):ti,ab,kw OR (thrombo* NEAR/1 prophyla*):ti,ab,kw

#19 #17 OR #18
#20 #6 AND #16 AND #19
#21 #20 NOT #7
#22 #21 NOT #8
#23 #22 AND #9
#24 #23 AND #10
#25 #24 AND #11
#26 #25 AND ('article'/it OR 'article in press'/it OR 'erratum'/it)

Search strategy 2021, Additional question on intraosseous access, Embase (via Elsevier)
Date: 06.05.2021 15 Hits

#1 'multiple trauma'/exp
#2 (polytrauma* OR "trauma patient?"):ti,ab,kw OR (severe NEXT/2 shock):ti,ab,kw
#3 ((multiple OR major OR severe* OR serious*) NEXT/3 (trauma* OR injur*)):ti,ab,kw
#4 ((blunt OR penetrating) NEXT/5 (trauma* OR injur*)):ti,ab,kw
#5 ('intensive care'/mj OR 'emergency'/mj OR ("life threatening" OR "critical care" OR emergen*):ti,ab,kw) AND (trauma* OR injur*):ti,ab,kw
#6 #1 OR #2 OR #3 OR #4 #5
#7 'animals'/exp NOT 'humans'/de
#8 (comment OR editorial OR letter):it OR "case report*":ti,ab,kw
#9 [embase]/lim
#10 embase NOT (embase AND medline)
#11 'intraosseous drug administration'/exp
#12 (intraosseous NEAR/2 (infusion? OR puncture? OR access OR device?)):ti,ab,kw
#13 #11 OR #12
#14 #6 AND #13
#15 #14 NOT #7
#16 #15 NOT #8
#17 #16 AND #9
#18 #17 AND #10
#19 #18 AND ('article'/it OR 'article in press'/it OR 'review'/it)
#20 [1-1-1947]/sd NOT [23-3-2021]/sd
#21 #19 AND #20

Search strategy 2021, Additional questions on access/catheterisation, Embase (via Elsevier)
Date: 06.05.2021 17 Hits

#1 'multiple trauma'/exp
#2 (polytrauma* OR "trauma patient?"):ti,ab,kw OR (severe NEXT/2 shock):ti,ab,kw
#3 ((multiple OR major OR severe* OR serious*) NEXT/3 (trauma* OR injur*)):ti,ab,kw
#4 ((blunt OR penetrating) NEXT/5 (trauma* OR injur*)):ti,ab,kw
#5 ('intensive care'/mj OR 'emergency'/mj OR ("life threatening" OR "critical care" OR emergen*):ti,ab,kw) AND (trauma* OR injur*):ti,ab,kw
#6 #1 OR #2 OR #3 OR #4 #5
#7 'animals'/exp NOT 'humans'/de
#8 (comment OR editorial OR letter):it OR "case report*":ti,ab,kw
#9 [embase]/lim
#10 embase NOT (embase AND medline)

#11 'hemorrhagic shock'/exp OR 'hypovolemic shock'/exp OR 'traumatic shock'/exp OR 'exsanguination'/exp OR 'hypovolemia'/exp

#12 (((uncontrolled OR uncontrollable OR acute OR active OR massive OR "life threatening" OR severe) NEAR/2 (bleeding OR h$emorrhage)) OR ((hypovol$emic OR h$emorrhagic OR traumatic) NEXT/1 (shock OR trauma*)) OR "damage control resuscitation"):ti,ab,kw

#13 'hemostasis'/exp OR 'blood clotting'/exp OR 'disseminated intravascular clotting'/exp OR 'bleeding'/exp

#14 (h$emosta* OR coagula* OR clotting OR coagulopath*OR h$emorrhag* OR bleed*):ti,ab,kw

#15 #11 OR #12 OR #13 OR #14

#16 'central venous catheterization'/exp

#17 (((“heat exchang*” OR thermoregulatory OR warming OR hypothermia) NEXT/4 catheter) OR ((endovascular OR intravascular) NEXT/1 cooling)):ti,ab,kw OR (((“central venous” OR arterial) NEXT/1 catheter*) AND (subclavian OR femoral* OR jugular*)):ti,ab,kw OR (((intravascular OR vascular OR intravenous OR venous OR i.v. OR vein OR arterial) NEXT/1 (access* or cannulation)):ti,ab,kw AND ('echography'/exp OR (ultrasonography* OR ultrasound):ti,ab,kw))

#18 #16 OR #17

#19 #6 AND #15 AND #18

#20 #19 NOT #7

#21 #20 NOT #8

#22 #21 AND #9

#23 #22 AND #10

#24 #23 AND ('article'/it OR 'review'/it)

#25 [1-1-1947]/sd NOT [23-3-2021]/sd

#26 #15 AND #20

Table S3. List of excluded studies

Study	Year	Title	Reason for exclusion
Abdoulhossein, D., I. Taheri, M. A. Saba, H. Akbari, S. Shafagh and A. Zataollah	2018	Effect of vitamin C and vitamin E on lung contusion: A randomized clinical trial study	not relevant to any question
Abt, R., T. Lustenberger, J. F. Stover, E. Benninger, P. M. Lenzlinger, R. Stocker and M. Keel	2009	Base excess determined within one hour of admission predicts mortality in patients with severe pelvic fractures and severe hemorrhagic shock	Study type/Outcome
Adair, K. E., J. D. Patrick, E. J. Kliber, M. N. Peterson, Holl and S. R.	2020	TXA (Tranexamic Acid) Risk Evaluation in Combat Casualties (TRECC)	Study type/Outcome
Ageron, F. X., A. Gayet-Ageron, K. Ker, T. J. Coats, H. Shakur-Still, I. Roberts and C. Antifibrinolytics Trials	2020	Effect of tranexamic acid by baseline risk of death in acute bleeding patients: a meta-analysis of individual patient-level data from 28 333 patients	Population
Ageron, F. X., T. J. Coats, V. Darioli and I. Roberts	2021	Validation of the BATT score for prehospital risk stratification of traumatic haemorrhagic death: usefulness for tranexamic acid treatment criteria	Study type/Outcome
Agyabeng-Dadzie, K., J. E. Hunter, T. R. Smith, M. Jordan, K. Safcsak, J. A. Ibrahim, M. L. Cheatham and I. S. Bhullar	2020	Antiplatelet Agent Reversal Is Unnecessary in Blunt Traumatic Brain Injury Patients Not Requiring Immediate Craniotomy	Study type/Outcome
Albreiki, M. and D. Voegeli	2018	Permissive hypotensive resuscitation in adult patients with traumatic haemorrhagic shock: a systematic review	Multiple publication without additional information
Allam, M. G. I. M.	2020	Activated factor seven (Afvii) versus aminocaproic acid for treatment of traumatic retro-peritoneal hematoma	not relevant to any question
Almuwallad, A., E. Cole, J. Ross, Z. Perkins and R. Davenport	2021	The Impact of Pre-Hospital TXA on Mortality among Bleeding Trauma Patients: A Systematic Review and Meta-Analysis	Study type/Outcome
Alsawadi, A.	2012	The clinical effectiveness of permissive hypotension in blunt abdominal trauma with hemorrhagic shock but without head or spine injuries or burns: a systematic review	Population
Anderson, T. N., H. E. Hinson, E. N. Dewey, E. A. Rick, M. A. Schreiber and S. E. Rowell	2020	Early Tranexamic Acid Administration After Traumatic Brain Injury Is Associated With Reduced Syndecan-1 and Angiopoietin-2 in Patients With Traumatic Intracranial Hemorrhage	Population
Auten, J. D., N. L. Lunceford, J. L. Horton, M. R. Galarneau, R. M. Galindo, C. D. Shepps, T. J. Zieber and C. B. Dewing	2015	The safety of early fresh, whole blood transfusion among severely battle injured at US Marine Corps forward surgical care facilities in Afghanistan	not relevant to any question

Study	Year	Title	Reason for exclusion
Avery, P., S. Morton, H. Tucker, L. Green, A. Weaver and R. Davenport	2020	Whole blood transfusion versus component therapy in adult trauma patients with acute major haemorrhage	Study type/Outcome
Baksaas-Aasen, K., S. Van Dieren, K. Balvers, N. P. Juffermans, P. A. Naess, C. Rourke, S. Eaglestone, S. R. Ostrowski, J. Stensballe, S. Stanworth, M. Maegele, J. C. Goslings, P. I. Johansson, K. Brohi, C. Gaarder and T. I. collaborators	2019	Data-driven Development of ROTEM and TEG Algorithms for the Management of Trauma Hemorrhage: A Prospective Observational Multicenter Study	Population
Barrett, C. D., N. Vigneshwar, H. B. Moore, A. Ghasabyan, Ch, J. ler, E. E. Moore and M. B. Yaffe	2020	Tranexamic acid is associated with reduced complement activation in trauma patients with hemorrhagic shock and hyperfibrinolysis on thromboelastography	Study type/Outcome
Berndtson, A. E., T. W. Costantini, J. Lane, K. Box and R. Coimbra	2016	If some is good, more is better: An enoxaparin dosing strategy to improve pharmacologic venous thromboembolism prophylaxis	Study type/Outcome
Bohonek, M., D. Kutac, L. ova, M. Koranova, E. Sladkova, E. Staskova, M. Voldrich and T. Tyll	2019	The use of cryopreserved platelets in the treatment of polytraumatic patients and patients with massive bleeding	Population
Bohonek, M., D. Kutác, L. ová, M. Koránová, E. Sládková, E. Stašková, M. Voldrich and T. Tyll	2016	Frozen platelets in clinical praxis: Comparative study of native platelets	Sprache
Boon, Y., W. S. Kuan, Y. H. Chan, I. Ibrahim and M. T. Chua	2021	Agreement between arterial and venous blood gases in trauma resuscitation in emergency department (AGREE)	Population
Borgman, M. A., M. Zaar, J. K. Aden, Z. J. Schlader, D. Gagnon, E. Rivas, J. Kern, N. J. Koons, V. A. Convertino, A. P. Cap, Cr and C. all	2019	Hemostatic responses to exercise, dehydration, and simulated bleeding in heat-stressed humans	Population
Boudreau, R. M., Deshp, K. K. e, G. M. Day, W. R. Hinckley, N. Harger, T. A. Pritts, A. T. Makley and M. D. Goodman	2019	Prehospital Tranexamic Acid Administration During Aeromedical Transport After Injury	Study type/Outcome
Boutin, A., L. Moore, F. Lauzier, M. Chasse, S. English, R. Zarychanski, L. McIntyre, D. Griesdale, D. A. Fergusson and A. F. Turgeon	2017	Transfusion of red blood cells in patients with traumatic brain injuries admitted to Canadian trauma health centres: a multicentre cohort study	Population
Bradburn, E. H., K. M. Ho, M. E. Morgan, L. D'Andrea, T. M. Vernon and F. B. Rogers	2021	Massive Transfusion Protocol and Subsequent Development of Venous Thromboembolism: Statewide Analysis	Population
Brilej, D., D. Stropnik, R. Lefering and R. Komadina	2017	Algorithm for activation of coagulation support treatment in multiple injured patients--cohort study	not relevant to any question
Brohi, K. and S. Eaglestone	2017	Traumatic coagulopathy and massive transfusion: improving outcomes and saving blood	Study type/Outcome
Brown, J. B., M. D. Neal, F. X. Guyette, A. B. Peitzman, T. R. Billiar, B. S. Zuckerbraun and J. L. Sperry	2015	Design of the Study of Tranexamic Acid during Air Medical Prehospital Transport (STAAMP) Trial: Addressing the Knowledge Gaps	Study type/Outcome

Study	Year	Title	Reason for exclusion
Bugaev, N., J. J. Como, G. Golani, J. J. Freeman, J. S. Sawhney, C. J. Vatsaas, B. K. Yorkgitis, L. A. Kreiner, N. M. Garcia, H. A. Aziz, P. A. Pappas, E. J. Mahoney, Z. W. Brown and G. Kasotakis	2020	Thromboelastography and rotational thromboelastometry in bleeding patients with coagulopathy: Practice management guideline from the Eastern Association for the Surgery of Trauma	Study type/Outcome
Byars, D. V., S. N. Tsuchitani, E. Erwin, B. Anglemyer and J. Eastman	2011	Evaluation of success rate and access time for an adult sternal intraosseous device deployed in the prehospital setting	Population
Callcut, R. A., M. W. Cripps, M. F. Nelson, A. S. Conroy, B. B. Robinson and M. J. Cohen	2016	The Massive Transfusion Score as a decision aid for resuscitation: Learning when to turn the massive transfusion protocol on and off	Study type/Outcome
Cannon, J. W., M. A. Khan, A. S. Raja, M. J. Cohen, J. J. Como, B. A. Cotton, J. J. Dubose, E. E. Fox, K. Inaba, C. J. Rodriguez, J. B. Holcomb and J. C. Duchesne	2017	Damage control resuscitation in patients with severe traumatic hemorrhage: A practice management guideline from the Eastern Association for the Surgery of Trauma	Study type/Outcome
Carothers, C., A. Giancarelli, J. Ibrahim and B. Hobbs	2018	Activated prothrombin complex concentrate for warfarin reversal in traumatic intracranial hemorrhage	Study type/Outcome
Chakroun-Walha, O., A. Samet, M. Jerbi, A. Nasri, A. Talbi, H. Kanoun, B. Souissi, K. Chtara, M. Bouaziz, H. Ksibi and N. Rekik	2019	Benefits of the tranexamic acid in head trauma with no extracranial bleeding: a prospective follow-up of 180 patients	Population
Chang, R., E. E. Fox, T. J. Greene, M. D. Swartz, S. M. DeSantis, D. M. Stein, E. M. Bulger, S. M. Melton, M. D. Goodman, M. A. Schreiber, M. D. Zielinski, T. O'Keeffe, K. Inaba, J. S. Tomasek, J. M. Podbielski, S. Appana, M. Yi, P. I. Johansson, H. H. He	2018	Abnormalities of laboratory coagulation tests versus clinically evident coagulopathic bleeding: results from the prehospital resuscitation on helicopters study (PROHS)	Study type/Outcome
Chapman, M. P., E. E. Moore, T. L. Chin, A. Ghasabyan, Ch, J. ler, J. Stringham, E. Gonzalez, H. B. Moore, A. Banerjee, C. C. Silliman and A. Sauaia	2015	Combat: Initial Experience with a Randomized Clinical Trial of Plasma-Based Resuscitation in the Field for Traumatic Hemorrhagic Shock	Multiple publication without additional information
Choi, S., M. H. Rahbar, J. Ning, D. J. Del Junco, E. Rahbar, C. Hong, J. Piao, E. E. Fox and J. B. Holcomb	2016	Recurrent event frailty models reduced time-varying and other biases in evaluating transfusion protocols for traumatic hemorrhage	Study type/Outcome
Chow, J. H., B. Fedeles, J. E. Richards, K. A. Tanaka, J. J. Morrison, P. Rock, T. M. Scalea, M. A. Mazzeffi and T. R.-T. Investigators	2020	Thromboelastography Reaction-Time Thresholds for Optimal Prediction of Coagulation Factor Deficiency in Trauma	Study type/Outcome
Coats, T. J. and M. Morsy	2020	Biological mechanisms and individual variation in fibrinolysis after major trauma	Intervention
Coccolini, F., G. Pizzilli, D. Corbella, M. Sartelli, V. Agnoletti, V. Agostini, G. L. Baiocchi, L. Ansaloni and F. Catena	2019	Pre-hospital plasma in haemorrhagic shock management: current opinion and meta-analysis of randomized trials	not relevant to any question
Cole, E., R. Davenport, K. Willett and K. Brohi	2015	Tranexamic acid use in severely injured civilian patients and the effects on outcomes: a prospective cohort study	not relevant to any question

Study	Year	Title	Reason for exclusion
Connelly, C. R., P. Y. Van, K. D. Hart, S. G. Louis, K. A. Fair, A. S. Erickson, E. A. Rick, E. C. Simeon, E. M. Bulger, S. Arbabi, J. B. Holcomb, L. J. Moore and M. A. Schreiber	2016	Thrombelastography-Based Dosing of Enoxaparin for Thromboprophylaxis in Trauma and Surgical Patients: A Randomized Clinical Trial	Population
Consunji, R., A. Elseed, A. El-Menyar, B. Sathian, S. Rizoli, H. Al-Thani and R. Peralta	2020	The effect of massive transfusion protocol implementation on the survival of trauma patients: a systematic review and meta-analysis	Population
Corbett, J. M., K. M. Ho and S. Honeybul	2019	Prognostic significance of abnormal hematological parameters in severe traumatic brain injury requiring decompressive craniectomy	Study type/Outcome
Cornero, S. G., M. Maegele, R. Lefering, C. Abbati, S. Gupta, F. Sammartano, S. Cimbanassi and O. Chiara	2020	Predictive Factors for Massive Transfusion in Trauma: A Novel Clinical Score from an Italian Trauma Center and German Trauma Registry	Study type/Outcome
Cruciani, M., M. Franchini, C. Mengoli, G. Marano, I. Pati, F. Masiello, E. Veropalumbo, S. Pupella, S. Vaglio, V. Agostini and G. M. Liumbruno	2021	The use of whole blood in traumatic bleeding: a systematic review	Multiple publication without additional information
Curry, N., C. Rourke, R. Davenport, S. Beer, L. Pankhurst, A. Deary, H. Thomas, C. Llewelyn, L. Green, H. Doughty, G. Nordmann, K. Brohi and S. Stanworth	2015	Early cryoprecipitate for major haemorrhage in trauma: a randomised controlled feasibility trial	not relevant to any question
Da Luz, L. T., B. Nascimento, A. K. Shankarakutty, S. Rizoli and N. K. Adhikari	2014	Effect of thromboelastography (TEG R) and rotational thromboelastometry (ROTEM R) on diagnosis of coagulopathy, transfusion guidance and mortality in trauma: descriptive systematic review	Study type/Outcome
da Luz, L. T., P. S. Shah, R. Strauss, A. A. Mohammed, P. P. D'Empaire, H. Tien, A. B. Nathens and B. Nascimento	2019	Does the evidence support the importance of high transfusion ratios of plasma and platelets to red blood cells in improving outcomes in severely injured patients: a systematic review and meta-analyses	Multiple publication without additional information
de Crescenzo, C., F. Gorouhi, E. S. Salcedo and J. M. Galante	2017	Prehospital hypertonic fluid resuscitation for trauma patients: A systematic review and meta-analysis	Multiple publication without additional information
Dias, J. D., C. G. Lopez-Espina, J. Ippolito, L. H. Hsiao, F. Zaman, A. A. Muresan, S. G. Thomas, M. Walsh, A. J. Jones, A. Grisoli, B. C. Thurston, R. Artang, K. P. Bilden, J. Hartmann and H. E. Achneck	2019	Rapid point-of-care detection and classification of direct-acting oral anticoagulants with the TEG 6s: Implications for trauma and acute care surgery	Population
Ditillo, M., K. Hanna, L. Castanon, M. Zeeshan, N. Kulvatunyou, A. Tang, J. Sakran, L. Gries and B. Joseph	2020	The role of cryoprecipitate in massively transfused patients: Results from the Trauma Quality Improvement Program database may change your mind	not relevant to any question

Study	Year	Title	Reason for exclusion
Dubendorfer, C., A. T. Billeter, B. Seifert, M. Keel and M. Turina	2013	Serial lactate and admission SOFA scores in trauma: an analysis of predictive value in 724 patients with and without traumatic brain injury	Study type/Outcome
Duchesne, J., A. Smith, S. Lawicki, J. Hunt, A. Houghton, S. Taghavi, R. Schroll, O. Jackson-Weaver, C. Guidry and D. Tatum	2020	Single Institution Trial Comparing Whole Blood vs Balanced Component Therapy: 50 Years Later	not relevant to any question
Dunham, C. M., R. J. Malik, G. S. Huang, C. M. Kohli, B. P. Brocker and K. T. Ugokwe	2018	Hypertonic saline administration and complex traumatic brain injury outcomes: a retrospective study	Study type/Outcome
Ebrahimi, P., J. Mozafari, R. B. Ilkhchi, M. G. Hanafi and M. Mousavinejad	2019	Intravenous Tranexamic Acid for Subdural and Epidural Intracranial Hemorrhage: Randomized, Double-Blind, Placebo-Controlled Trial	Population
Ellenberger, C., N. Garofano, G. Barcelos, J. Diaper, G. Pavlovic and M. Licker	2017	Assessment of Haemostasis in patients undergoing emergent neurosurgery by rotational Elastometry and standard coagulation tests: a prospective observational study	Population
El-Menyar, A., B. Sathian, M. Asim, R. Latifi and H. Al-Thani	2018	Efficacy of prehospital administration of tranexamic acid in trauma patients: A meta-analysis of the randomized controlled trials	Study type/Outcome
Endo, A., A. Shiraishi, Y. Otomo, S. Kushimoto, D. Saitoh, M. Hayakawa, H. Ogura, K. Murata, A. Hagiwara, J. Sasaki, T. Matsuoka, T. Uejima, N. Morimura, H. Ishikura, M. Takeda, N. Kaneko, H. Kato, D. Kudo, T. Kanemura, T. Shibusawa, Y. Hagiwara, S. Furug	2016	Development of Novel Criteria of the "Lethal Triad" as an Indicator of Decision Making in Current Trauma Care: A Retrospective Multicenter Observational Study in Japan	not relevant to any question
Fadeyi, E. A., A. K. Saha, T. Naal, H. Martin, E. Fenu, J. H. Simmons, M. R. Jones and G. J. Pomper	2020	A comparison between leukocyte reduced low titer whole blood vs non-leukocyte reduced low titer whole blood for massive transfusion activation	not relevant to any question
Faraoni, D. and P. D. Van Linden	2014	A systematic review of antifibrinolytics and massive injury	Multiple publication without additional information
Fei, A., Q. Lin, J. Liu, F. Wang, H. Wang and S. Pan	2015	The relationship between coagulation abnormality and mortality in ICU patients: a prospective, observational study	Population
Fischer, M., P. Lackner, R. Beer, R. Helbok, B. Pfausler, D. Schneider, E. Schmutzhard and G. Broessner	2015	Cooling Activity is Associated with Neurological Outcome in Patients with Severe Cerebrovascular Disease Undergoing Endovascular Temperature Control	Population
Fletcher-S, A. ersjoo, E. P. Thelin, M. Maegele, M. Svensson, Bell and B. M. er	2020	Time Course of Hemostatic Disruptions After Traumatic Brain Injury: A Systematic Review of the Literature	Population

Study	Year	Title	Reason for exclusion
Fragou, M., A. Gravvanis, V. Dimitriou, A. Papalois, G. Kouraklis, A. Karabinis, T. Saranteas, J. Poularas, J. Papanikolaou, P. Davlouros, N. Labropoulos and D. Karakitsos	2011	Real-time ultrasound-guided subclavian vein cannulation versus the landmark method in critical care patients: a prospective randomized study	Population
Franz, N. D., Machado-Ar, D. a, J. T. Miller and N. Farina	2020	Impact of Obesity on Tranexamic Acid Efficacy in Adult Patients With Major Bleeding	Study type/Outcome
Fu, H. P., Y. X. Zhang, Y. F. Wei, Z. Q. Wang, G. X. Wang and X. L. Li	2018	Correlation analysis between both the platelet-to-lymphocyte ratio and maximum amplitude of thrombus and death in severe traumatic patients	Sprache
Furmaga, W., S. Cohn, T. J. Prihoda, M. T. Muir, V. Mikhailov, J. McCarthy and Y. Arar	2015	Novel markers predict death and organ failure following hemorrhagic shock	not relevant to any question
Gao, Y. K., Gui, C. J., Xin, W. Q., Hu, D., Yang, X. Y.	2019	Assessment of mild hypothermia combined with edaravone for the treatment of severe craniocerebral injury	Intervention
Giancarelli, A., K. L. Birrer, R. F. Alban, B. P. Hobbs and X. Liu-DeRyke	2016	Hypocalcemia in trauma patients receiving massive transfusion	Study type/Outcome
Godfrey, B. W., A. Martin, P. J. Chestovich, G. H. Lee, N. K. Ingalls and V. Saldanha	2017	Patients with multiple traumatic amputations: An analysis of operation enduring freedom joint theatre trauma registry data	Intervention
Gonzalez-Guerrero, C., T. Lozano-Andreu, M. Roch-Santed, L. Rivera-Sanchez, Br, D. ariz-Nunez, L. Pasto-Cardona, J. C. Juarez-Gimenez and J. B. Montoro-Ronsano	2017	Evaluation of the efficiency under current use of human fibrinogen concentrate in trauma patients with life-threatening hemorrhagic disorders	Intervention
Gozal, Y. M., C. P. Carroll, B. M. Krueger, J. Khoury and N. O. Andaluz	2017	Point-of-care testing in the acute management of traumatic brain injury: Identifying the coagulopathic patient	Study type/Outcome
Grba-Bujević, M., I. Bošan-Kilibarda and N. Strikić	2012	The use of hypertonic-hyperoncotic solution for hypovolemic shock in trauma patients in prehospital setting	Study type/Outcome
Gunning, A. C., R. V. Maier, D. de Rooij, L. P. H. Leenen and F. Hietbrink	2021	Venous thromboembolism (VTE) prophylaxis in severely injured patients: an international comparative assessment	Study type/Outcome
Guo, S. B., Y. X. Chen and X. Z. Yu	2017	Clinical Characteristics and Current Interventions in Shock Patients in Chinese Emergency Departments: A Multicenter Prospective Cohort Study	Intervention
Haas, T., K. Gorlinger, A. Grassetto, V. Agostini, P. Simioni, G. Nardi and M. Ranucci	2014	Thromboelastometry for guiding bleeding management of the critically ill patient: a systematic review of the literature	Study type/Outcome
Hanna, K., L. Bible, M. Chehab, S. Asmar, M. Douglas, M. Ditillo, L. Castanon, A. Tang and B. Joseph	2020	Nationwide analysis of whole blood hemostatic resuscitation in civilian trauma	not relevant to any question
Hartholt, K. A., E. M. M. van Lieshout, W. C. Thies, P. Patka and I. B. Schipper	2010	Intraosseous devices: a randomized controlled trial comparing three intraosseous devices	not relevant to any question

Study	Year	Title	Reason for exclusion
Haverkamp, F. J. C., G. G. Giesbrecht and E. Tan	2018	The prehospital management of hypothermia - An up-to-date overview	Study type/Outcome
Hazelton, J. P., J. W. Cannon, C. Zatorski, J. S. Roman, S. A. Moore, A. J. Young, M. Subramanian, J. F. Guzman, F. Fogt, A. Moran, J. Gaughan, M. J. Seamon and J. Porter	2019	Cold-stored whole blood: A better method of trauma resuscitation?	not relevant to any question
Heidari, K., M. Taghizadeh, S. Mahmoudi, H. Panahi, E. Ghaffari Shad and S. Asadollahi	2017	FAST for blunt abdominal trauma: Correlation between positive findings and admission acid-base measurement	Population
Heming, N., S. Elatrous, S. Jaber, A. S. Dumenil, J. Cousson, X. Forceville, A. Kimmoun, J. L. Trouillet, J. Fichet, N. Anguel, M. Darmon, C. Martin, S. Chevret, D. Annane and C. Investigators	2017	Haemodynamic response to crystalloids or colloids in shock: an exploratory subgroup analysis of a randomised controlled trial	Population
Hoedemaekers, C. W., M. Ezzahti, A. Gerritsen and J. G. van der Hoeven	2007	Comparison of cooling methods to induce and maintain normo- and hypothermia in intensive care unit patients: a prospective intervention study	Population
Holcomb, J. B., D. P. Donathan, B. A. Cotton, D. J. Del Junco, G. Brown, T. V. Wenckstern, J. M. Podbielski, E. A. Camp, R. Hobbs, Y. Bai, M. Brito, E. Hartwell, J. R. Duke and C. E. Wade	2015	Prehospital Transfusion of Plasma and Red Blood Cells in Trauma Patients	Population
Huang, G. S. and C. M. Dunham	2017	Mortality outcomes in trauma patients undergoing prehospital red blood cell transfusion: a systematic literature review	Study type/Outcome
Huebner, B. R., W. C. Dorlac and C. Cribari	2017	Tranexamic Acid Use in Prehospital Uncontrolled Hemorrhage	Population
Human, T., A. M. Cook, B. Anger, K. Bledsoe, A. Castle, D. Deen, H. Gibbs, C. Lesch, N. Liang, K. McAllen, C. Morrison, D. Parker, Jr., A. S. Rowe, D. Rhoney, K. Sangha, E. Santayana, S. Taylor, E. Tesoro and G. Brophy	2017	Treatment of Hyponatremia in Patients with Acute Neurological Injury	Population
Hunt, H., S. Stanworth, N. Curry, T. Woolley, C. Cooper, O. Ukoumunne, Z. Zhelev and C. Hyde	2015	Thromboelastography (TEG) and rotational thromboelastometry (ROTEM) for trauma induced coagulopathy in adult trauma patients with bleeding	Study type/Outcome
Imran, J. B., T. D. Madni, A. T. Clark, P. Rizk, E. Huang, C. T. Minshall, L. R. Taveras, H. B. Cunningham, A. L. Eastman, J. P. Koshy, C. D. Kacir and M. W. Cripps	2018	Inability to predict subprophylactic anti-factor Xa levels in trauma patients receiving early low-molecular-weight heparin	Study type/Outcome
Ishii, K., T. Kinoshita, K. Kiridume, A. Watanabe, K. Yamakawa, S. Nakao, S. Fujimi and T. Matsuoka	2019	Impact of initial coagulation and fibrinolytic markers on mortality in patients with severe blunt trauma: a multicentre retrospective observational study	Study type/Outcome
Jachetti, A., R. B. Massenat, N. Edema, S. C. Woolley, G. Benedetti, R. Van Den Bergh and M. Trelles	2019	Introduction of a standardised protocol, including systematic use of tranexamic acid, for management of severe adult	Study type/Outcome

Study	Year	Title	Reason for exclusion
		trauma patients in a low-resource setting: the MSF experience from Port-au-Prince, Haiti	
Javali, R. H., P. Ravindra, A. Patil, M. Srinivasarangan, H. Mundada, S. B. Adarsh and S. Nisarg	2017	A Clinical Study on the Initial Assessment of Arterial Lactate and Base Deficit as Predictors of Outcome in Trauma Patients	Population
Javier, P. T. C., G. G. Celia, B. R. Virginia, M. T. Javier, L. A. Antonio and M. R. J. Bruno	2019	Use of fibrinogen concentrate in surgical and bleeding trauma patients	Study type/Outcome
Jehan, F., T. O'Keeffe, M. Khan, A. Chi, A. Tang, N. Kulvatunyou, L. Gries and B. Joseph	2017	Early thromboprophylaxis with low-molecular-weight heparin is safe in patients with pelvic fracture managed nonoperatively	Population
Jensen, K. O., L. Held, A. Kraus, Hildebr, F. , P. Mommsen, L. Mica, G. A. Wanner, P. Steiger, R. M. Moos, H. P. Simmen and K. Sprengel	2016	The impact of mild induced hypothermia on the rate of transfusion and the mortality in severely injured patients: a retrospective multi-centre study	Study type/Outcome
Johnston, L. R., C. J. Rodriguez, E. A. Elster and M. J. Bradley	2018	Evaluation of Military Use of Tranexamic Acid and Associated Thromboembolic Events	Study type/Outcome
Jokar, A., K. Ahmadi, T. Salehi, M. Sharif-Alhoseini and V. Rahimi-Movaghar	2017	The effect of tranexamic acid in traumatic brain injury: A randomized controlled trial	Population
Joseph, B., H. Aziz, M. Snell, V. Pandit, D. Hays, N. Kulvatunyou, A. Tang, T. O'Keeffe, J. Wynne, R. S. Friese and P. Rhee	2014	The physiological effects of hyperosmolar resuscitation: 5% vs 3% hypertonic saline	Study type/Outcome
Joseph, B., V. it, C. Harrison, D. Lubin, N. Kulvatunyou, B. Zangbar, A. Tang, T. O'Keeffe, D. J. Green, L. Gries, R. S. Friese and P. Rhee	2015	Early thromboembolic prophylaxis in patients with blunt solid abdominal organ injuries undergoing nonoperative management: is it safe?	Study type/Outcome
Jouffroy, R. and B. Vivien	2020	Prehospital Plasma Transfusion and Survival in Trauma Patients With Hemorrhagic Shock	Study type/Outcome
Kang, W. S., I. S. Shin, J. S. Pyo, S. Ahn, S. Chung, Y. J. Ki, J. Seok, C. Y. Park and S. Lee	2019	Prognostic Accuracy of Massive Transfusion, Critical Administration Threshold, and Resuscitation Intensity in Assessing Mortality in Traumatic Patients with Severe Hemorrhage: a Meta-Analysis	Study type/Outcome
Kaserer, A., M. Casutt, K. Sprengel, B. Seifert, D. R. Spahn and P. Stein	2018	Comparison of two different coagulation algorithms on the use of allogenic blood products and coagulation factors in severely injured trauma patients: a retrospective, multicentre, observational study	Study type/Outcome
Kasotakis, G., N. Starr, E. Nelson, B. Sarkar, P. A. Burke, D. G. Remick, R. G. Tompkins, Inflammation and I. Host Response to Injury	2019	Platelet transfusion increases risk for acute respiratory distress syndrome in non-massively transfused blunt trauma patients	not relevant to any question
Ker, K., I. Roberts, H. Shakur and T. J. Coats	2015	Antifibrinolytic drugs for acute traumatic injury	Population

Study	Year	Title	Reason for exclusion
Khan, S., R. Davenport, I. Raza, S. Glasgow, H. D. De'Ath, P. I. Johansson, N. Curry, S. Stanworth, C. Gaarder and K. Brohi	2015	Damage control resuscitation using blood component therapy in standard doses has a limited effect on coagulopathy during trauma hemorrhage	Study type/Outcome
Kim, J. H., A. Nagy, A. Putzu, A. Belletti, G. Biondi-Zoccai, V. V. Likhvantsev, A. G. Yavorovskiy and G. oni	2020	Therapeutic Hypothermia in Critically Ill Patients: A Systematic Review and Meta-Analysis of High Quality Randomized Trials	Population
Kornblith, L. Z., A. J. Robles, A. S. Conroy, B. J. Redick, B. M. Howard, C. M. Hendrickson, S. Moore, M. F. Nelson, F. Moazed, R. A. Callcut, C. S. Calfee and M. J. Cohen	2019	Predictors of postinjury acute respiratory distress syndrome: Lung injury persists in the era of hemostatic resuscitation	Study type/Outcome
Kreutziger, J., A. Rafetseder, S. Mathis, V. Wenzel, R. El Attal and S. Schmid	2015	Admission blood glucose predicted haemorrhagic shock in multiple trauma patients	Study type/Outcome
Kutcher, M. E., B. M. Howard, J. L. Sperry, A. E. Hubbard, A. L. Decker, J. Cuschieri, J. P. Minei, E. E. Moore, B. H. Brownstein, R. V. Maier and M. J. Cohen	2015	Evolving beyond the vicious triad: Differential mediation of traumatic coagulopathy by injury, shock, and resuscitation	Intervention
Kwan, I., F. Bunn, P. Chinnock and I. Roberts	2014	Timing and volume of fluid administration for patients with bleeding	Population
Laursen, T. H., M. A. S. Meyer, A. S. P. Meyer, T. Gaarder, P. A. Naess, J. Stensballe, S. R. Ostrowski and P. I. Johansson	2018	Thrombelastography early amplitudes in bleeding and coagulopathic trauma patients: Results from a multicenter study	Population
Lefering, R., D. Zielske, B. Bouillon, C. Hauser and H. Levy	2013	Lactic acidosis is associated with multiple organ failure and need for ventilator support in patients with severe hemorrhage from trauma	Intervention
Leidel, B. A., C. Kirchhoff, V. Bogner, J. Stegmaier, W. Mutschler, K.-G. Kanz and V. Braunstein	2009	Is the intraosseous access route fast and efficacious compared to conventional central venous catheterization in adult patients under resuscitation in the emergency department? A prospective observational pilot study	Population
Leidel, B. A., C. Kirchhoff, V. Braunstein, V. Bogner, P. Biberthaler and K.-G. Kanz	2010	Comparison of two intraosseous access devices in adult patients under resuscitation in the emergency department: A prospective, randomized study	Population
Lewis, C. J., P. Li, L. Stewart, A. C. Weintrob, M. L. Carson, C. K. Murray, D. R. Tribble and J. D. Ross	2016	Tranexamic acid in life-threatening military injury and the associated risk of infective complications	Population
Lewis, S. R., M. W. Pritchard, D. J. Evans, A. R. Butler, P. Alderson, A. F. Smith and I. Roberts	2018	Colloids versus crystalloids for fluid resuscitation in critically ill people	Population
Lim, G., K. Harper-Kirksey, R. Parekh and A. F. Manini	2018	Efficacy of a massive transfusion protocol for hemorrhagic trauma resuscitation	Study type/Outcome

Study	Year	Title	Reason for exclusion
Lin, W., X. Lin, Y. Zhuang, X. Pan, C. Wu, S. Zhang, L. Zhang, J. Lin, S. Shi and S. Shi	2019	Significance of Early Postoperative Arterial Lactic Acid, Inferior Vena Cava Variability, and Central Venous Pressure in Hypovolemic Shock	Study type/Outcome
Liu, Y. H., Z. D. Shang, C. Chen, N. Lu, Q. F. Liu, M. Liu and J. Yan	2015	'Cool and quiet' therapy for malignant hyperthermia following severe traumatic brain injury: A preliminary clinical approach	Study type/Outcome
Lombardo, S., D. Millar, G. J. Jurkovich, R. Coimbra and R. Nirula	2018	Factor VIIa administration in traumatic brain injury: an AAST-MITC propensity score analysis	Intervention
Luehr, E., G. Grone, M. Pathak, C. Austin and S. Thompson	2017	Administration of tranexamic acid in trauma patients under stricter inclusion criteria increases the treatment window for stabilization from 24 to 48 hours-a retrospective review	Study type/Outcome
Lui, C. T., O. F. Wong, K. L. Tsui, C. W. Kam, S. M. Li, M. Cheng and K. K. G. Leung	2018	Predictive model integrating dynamic parameters for massive blood transfusion in major trauma patients: The Dynamic MBT score	Population
Mackenzie, C. F., Y. Wang, P. F. Hu, S. Y. Chen, H. H. Chen, G. Hagegeorge, L. G. Stansbury, S. Shackelford and O. S. Group	2014	Automated prediction of early blood transfusion and mortality in trauma patients	Intervention
Madden, L. K., M. Hill, T. L. May, T. Human, M. M. Guanci, J. Jacobi, M. V. Moreda and N. Badjatia	2017	The Implementation of Targeted Temperature Management: An Evidence-Based Guideline from the Neurocritical Care Society	Study type/Outcome
Mahmood, A., K. Needham, H. Shakur-Still, T. Harris, S. F. Jamaluddin, D. Davies, A. Belli, F. L. Mohamed, C. Leech, H. M. Lotfi, P. Moss, F. Lecky, P. Hopkins, D. Wong, A. Boyle, M. Wilson, M. Darwent and I. Roberts	2020	Effect of tranexamic acid on intracranial haemorrhage and infarction in patients with traumatic brain injury: a pre-planned substudy in a sample of CRASH-3 trial patients	Population
Malkin, M., A. Nevo, S. I. Brundage and M. Schreiber	2021	Effectiveness and safety of whole blood compared to balanced blood components in resuscitation of hemorrhaging trauma patients - A systematic review	Study type/Outcome
Mangram, A., O. F. Oguntodu, Dz, J. K. u, A. K. Hollingworth, S. Hall, C. Cung, J. Rodriguez, I. Yusupov and J. F. Barletta	2016	Is there a difference in efficacy, safety, and cost-effectiveness between 3-factor and 4-factor prothrombin complex concentrates among trauma patients on oral anticoagulants?	Population
Marsden, M. E. R., A. Rossetto, C. A. B. Duffield, T. G. D. Woolley, W. P. Buxton, S. Steynberg, R. Bagga and N. R. M. Tai	2019	Prehospital tranexamic acid shortens the interval to administration by half in Major Trauma Networks: a service evaluation	Population
Masoumi, K., A. Forouzan, A. A. Darian and A. Rafaty Navaii	2016	Comparison of the Effectiveness of Hydroxyethyl Starch (Voluven) Solution With Normal Saline in Hemorrhagic Shock Treatment in Trauma	Study type/Outcome

Study	Year	Title	Reason for exclusion
Matsushima, K., E. Benjamin and D. Demetriades	2015	Prothrombin complex concentrate in trauma patients	Study type/Outcome
McCully, B. H., C. R. Connelly, K. A. Fair, J. B. Holcomb, E. E. Fox, C. E. Wade, E. M. Bulger, M. A. Schreiber and P. S. Group	2017	Onset of Coagulation Function Recovery Is Delayed in Severely Injured Trauma Patients with Venous Thromboembolism	Study type/Outcome
McMillen, J. C., C. M. Lawson and A. Shaun Rowe	2013	Futility Assessment of Recombinant Factor VII Activated for the Treatment of Hemorrhagic Shock Requiring Massive Transfusion	Study type/Outcome
McQuilten, Z. K., G. Crighton, S. Brunskill, J. K. Morison, T. H. Richter, N. Waters, M. F. Murphy and E. M. Wood	2018	Optimal Dose, Timing and Ratio of Blood Products in Massive Transfusion: Results from a Systematic Review	Population
McQuilten, Z. K., G. Crighton, S. Engelbrecht, R. Gotmaker, S. J. Brunskill, M. F. Murphy and E. M. Wood	2015	Transfusion interventions in critical bleeding requiring massive transfusion: a systematic review	Multiple publication without additional information
Meizoso, J. P., C. A. Karcutskie, J. J. Ray, N. Namias, C. I. Schulman and K. G. Proctor	2017	Persistent Fibrinolysis Shutdown Is Associated with Increased Mortality in Severely Injured Trauma Patients	Intervention
Mengoli, C., M. Franchini, G. Marano, S. Pupella, S. Vaglio, M. Marietta and G. M. Liumbruno	2017	The use of fibrinogen concentrate for the management of trauma-related bleeding: a systematic review and meta-analysis	Study type/Outcome
Mesghali, E., S. Fitter, K. Bahjri and K. Moussavi	2019	Safety of Peripheral Line Administration of 3% Hypertonic Saline and Mannitol in the Emergency Department	Study type/Outcome
Meyer, A. S., M. A. Meyer, A. M. Sorensen, L. S. Rasmussen, M. B. Hansen, J. B. Holcomb, B. A. Cotton, C. E. Wade, S. R. Ostrowski and P. I. Johansson	2014	Thrombelastography and rotational thromboelastometry early amplitudes in 182 trauma patients with clinical suspicion of severe injury	Study type/Outcome
Mica, L., H. Simmen, C. M. Werner, M. Plecko, C. Keller, S. H. Wirth and K. Sprengel	2016	Fresh frozen plasma is permissive for systemic inflammatory response syndrome, infection, and sepsis in multiple-injured patients	Study type/Outcome
Miyata, K., H. Ohnishi, K. Maekawa, T. Mikami, Y. Akiyama, S. Iihoshi, M. Wanibuchi, N. Mikuni, S. Uemura, K. Tanno, E. Narimatsu and Y. Asai	2016	Therapeutic temperature modulation in severe or moderate traumatic brain injury: a propensity score analysis of data from the Nationwide Japan Neurotrauma Data Bank	Intervention
Mizushima, Y., S. Nakao, K. Idoguchi and T. Matsuoka	2017	Fluid resuscitation of trauma patients: How much fluid is enough to determine the patient's response?	Intervention
Mojallal, F., M. Nikooieh, M. Hajimaghsoudi, M. Baqherabadi, M. Jafari, A. Esmaeili, N. M. Karimi and E. Zarepur	2020	The effect of intravenous tranexamic acid on preventing the progress of cerebral hemorrhage in patients with brain traumatic injuries compared to placebo: A randomized clinical trial	Population

Study	Year	Title	Reason for exclusion
Mok, G., R. Hoang, M. W. Khan, D. Pannell, H. Peng, H. Tien, A. Nathens, J. Callum, K. Karkouti, A. Beckett and L. T. da Luz	2021	Freeze-dried plasma for major trauma - Systematic review and meta-analysis	Study type/Outcome
Monsef Kasmaei, V., A. Javadi and S. A. Naseri Alavi	2019	Effects of tranexamic acid on reducing blood loss in pelvic trauma: A randomised double-blind placebo controlled study	Population
Moore, H. B., E. E. Moore, E. Gonzalez, M. P. Chapman, T. L. Chin, C. C. Silliman, A. Banerjee and A. Sauaia	2014	Hyperfibrinolysis, physiologic fibrinolysis, and fibrinolysis shutdown: the spectrum of postinjury fibrinolysis and relevance to antifibrinolytic therapy	Study type/Outcome
Moore, H. B., E. E. Moore, M. P. Chapman, K. C. Hansen, M. J. Cohen, F. M. Pieracci, Ch, J. ler and A. Sauaia	2019	Does Tranexamic Acid Improve Clot Strength in Severely Injured Patients Who Have Elevated Fibrin Degradation Products and Low Fibrinolytic Activity, Measured by Thrombelastography?	Population
Mousavinejad, M., J. Mozafari, R. B. Ilkhchi, M. G. Hanafi and P. Ebrahimi	2020	Intravenous Tranexamic Acid for Brain Contusion with Intraparenchymal Hemorrhage: Randomized, Double-Blind, Placebo-Controlled Trial	Population
Myers, S. P., M. R. Dyer, A. Hassoune, J. B. Brown, J. L. Sperry, M. P. Meyer, M. R. Rosengart and M. D. Neal	2020	Correlation of Thromboelastography with Apparent Rivaroxaban Concentration: Has Point-of-Care Testing Improved?	Population
Nadler, R., S. Gendler, A. Benov, R. Strugo, A. Abramovich and E. Glassberg	2014	Tranexamic acid at the point of injury: the Israeli combined civilian and military experience	Population
Naumann, D. N., J. Hazeldine, D. J. Davies, J. Bishop, M. J. Midwinter, A. Belli, P. Harrison and J. M. Lord	2018	Endotheliopathy of Trauma is an on-Scene Phenomenon, and is Associated with Multiple Organ Dysfunction Syndrome: A Prospective Observational Study	Study type/Outcome
Nederpelt, C. J., M. El Hechi, J. Parks, J. Fawley, A. E. Mendoza, N. Saillant, D. R. King, P. J. Fagenholz, G. C. Velmahos and H. M. A. Kaafarani	2020	The dose-dependent relationship between blood transfusions and infections after trauma: A population-based study	not relevant to any question
Neeki, M. M., F. Dong, J. Toy, J. Salameh, M. Rabiei, J. Powell, R. Vara, K. Inaba, D. Wong, M. E. Comunale, A. Lowe, Ch, D. wani, J. Quispe and R. Borger	2020	Safety and Efficacy of Hospital Utilization of Tranexamic Acid in Civilian Adult Trauma Resuscitation	not relevant to any question
Neeki, M. M., F. Dong, J. Toy, R. Vaezazizi, J. Powell, N. Jabourian, A. Jabourian, D. Wong, R. Vara, K. Seiler, T. W. Pennington, J. Powell, C. Yoshida-McMath, S. Kissel, K. Schulz-Costello, J. Mistry, M. S. Surrusco, K. R. O'Bosky, D. Van Stralen, D. Lu	2017	Efficacy and Safety of Tranexamic Acid in Prehospital Traumatic Hemorrhagic Shock: Outcomes of the Cal-PAT Study	Study type/Outcome
Nguyen, M., R. Pirracchio, L. Z. Kornblith, R. Callcut, E. E. Fox, C. E. Wade, M. Schreiber, J. B. Holcomb, J. Coyle, M. Cohen and A. Hubbard	2020	Dynamic impact of transfusion ratios on outcomes in severely injured patients: Targeted machine learning analysis of the	Study type/Outcome

Study	Year	Title	Reason for exclusion
		Pragmatic, Randomized Optimal Platelet and Plasma Ratios randomized clinical trial	
Nistor, M., W. Behringer, M. Schmidt and R. Schiffner	2017	A Systematic Review of Neuroprotective Strategies during Hypovolemia and Hemorrhagic Shock	Population
Novak, D. J., Y. Bai, R. K. Cooke, M. B. Marques, M. J. Fontaine, J. L. Gottschall, P. M. Carey, R. M. Scanlan, E. W. Fiebig, I. A. Shulman, J. M. Nelson, S. Flax, V. Duncan, J. A. Daniel-Johnson, J. L. Callum, J. B. Holcomb, E. E. Fox, S. Baraniuk, B. C.	2015	Making thawed universal donor plasma available rapidly for massively bleeding trauma patients: experience from the Pragmatic, Randomized Optimal Platelets and Plasma Ratios (PROPPR) trial	Study type/Outcome
Oberladstatter, D., W. Voelckel, C. Schlimp, J. Zipperle, B. Ziegler, O. Grottke and H. Schochl	2021	A prospective observational study of the rapid detection of clinically-relevant plasma direct oral anticoagulant levels following acute traumatic injury	Population
Ogura, T., Y. Nakamura, M. Nakano, Y. Izawa, M. Nakamura, K. Fujizuka, M. Suzukawa and A. T. Lefor	2014	Predicting the need for massive transfusion in trauma patients: the Traumatic Bleeding Severity Score	Intervention
Olaussen, A., J. Bade-Boon, M. C. Fitzgerald and B. Mitra	2018	Management of injured patients who were Jehovah's Witnesses, where blood transfusion may not be an option: a retrospective review	Population
Otsuka, H., N. Sakoda, A. Uehata, T. Sato, K. Sakurai, H. Aoki, T. Yamagiwa, S. Iizuka and S. Inokuchi	2020	Indications for early plasma transfusion and its optimal use following trauma	Study type/Outcome
Owattanapanich, N., K. Chittawatanarat, T. Benyakorn and J. Sirikun	2018	Risks and benefits of hypotensive resuscitation in patients with traumatic hemorrhagic shock: a meta-analysis	Study type/Outcome
Ozakin, E., N. O. Yazlamaz, F. B. Kaya, E. M. Karakilic and M. Bilgin	2020	Perfusion Index Measurement in Predicting Hypovolemic Shock in Trauma Patients	Study type/Outcome
Payen, J. F., M. Berthet, C. Genty, P. Declety, D. Garrigue-Huet, N. Morel, P. Bouzat, B. Riou, J. L. Bosson and i. Novoseven Trauma	2016	Reduced mortality by meeting guideline criteria before using recombinant activated factor VII in severe trauma patients with massive bleeding	Study type/Outcome
Perel, P., T. Clayton, D. G. Altman, P. Croft, I. Douglas, H. Hemingway, A. Hingorani, K. I. Morley, R. Riley, A. Timmis, D. Van der Windt, I. Roberts and P. Partnership	2014	Red blood cell transfusion and mortality in trauma patients: risk-stratified analysis of an observational study	not relevant to any question
Plurad, D. S., W. Chiu, A. S. Raja, S. M. Galvagno, U. Khan, D. Y. Kim, S. A. Tisherman, J. Ward, M. E. Hamill, V. Bennett, B. Williams and B. Robinson	2018	Monitoring modalities and assessment of fluid status: A practice management guideline from the Eastern Association for the Surgery of Trauma	not relevant to any question
Pommerening, M. J., M. D. Goodman, D. L. Farley, J. C. Cardenas, J. Podbielski, N. Matijevic, C. E. Wade, J. B. Holcomb and B. A. Cotton	2014	Early diagnosis of clinically significant hyperfibrinolysis using thrombelastography velocity curves	Study type/Outcome

Study	Year	Title	Reason for exclusion
Presneill, J., D. Gantner, A. Nichol, C. McArthur, A. Forbes, J. Kasza, T. Trapani, L. Murray, S. Bernard, P. Cameron, G. Capellier, O. Huet, L. Newby, S. Rashford, J. V. Rosenfeld, T. Smith, M. Stephenson, D. Varma, S. Vallance, T. Walker, S. Webb, D. Ja	2018	Statistical analysis plan for the POLAR-RCT: The Prophylactic hypOthermia trial to Lessen trAumatic bRain injury-Randomised Controlled Trial	Study type/Outcome
Puranik, G. N., T. Y. P. Verma and G. A. it	2018	The Study of Coagulation Parameters in Polytrauma Patients and Their Effects on Outcome	Study type/Outcome
Rhee, P., K. Inaba, V. it, M. Khalil, S. Siboni, G. Vercruysse, N. Kulvatunyou, A. Tang, A. Asif, T. O'Keeffe and B. Joseph	2015	Early autologous fresh whole blood transfusion leads to less allogeneic transfusions and is safe	Study type/Outcome
Rijnhout, T. W. H., K. E. Wever, R. Marinus, N. Hoogerwerf, L. M. G. Geeraedts, Jr. and E. Tan	2019	Is prehospital blood transfusion effective and safe in haemorrhagic trauma patients? A systematic review and meta-analysis	Study type/Outcome
Ritchie, D. T., F. G. A. Philbrook, S. Leadbitter, K. N. Kokwe, E. Meehan, M. McGeady and M. Beaton	2020	Empirical transfusion strategies for major hemorrhage in trauma patients: A systematic review	Multiple publication without additional information
Roberts, D. J., N. Bobrovitz, D. A. Zygun, A. W. Kirkpatrick, C. G. Ball, P. D. Faris, H. T. Stelfox and G. for the Indications for Trauma Damage Control Surgery International Study	2021	Evidence for use of damage control surgery and damage control interventions in civilian trauma patients: a systematic review	Intervention
Ross, S. W., A. B. Christmas, P. E. Fischer, H. Holway, A. L. Walters, R. Seymour, M. A. Gibbs, B. T. Heniford and R. F. Sing	2015	Impact of common crystalloid solutions on resuscitation markers following Class I hemorrhage: A randomized control trial	Population
Rowell, S. E., E. N. Meier, B. McKnight, D. Kannas, S. May, K. Sheehan, E. M. Bulger, A. H. Idris, J. Christenson, L. J. Morrison, R. J. Frascone, P. L. Bosarge, M. R. Colella, J. Johannigman, B. A. Cotton, J. Callum, J. McMullan, D. J. Dries, B. Tibbs, N	2020	Effect of Out-of-Hospital Tranexamic Acid vs Placebo on 6-Month Functional Neurologic Outcomes in Patients With Moderate or Severe Traumatic Brain Injury	Population
Safari, H., P. Farrahi, S. Rasras, Mar, H. J. i and M. Zeinali	2020	Effect of Intravenous Tranexamic Acid on Intracerebral Brain Hemorrhage in Traumatic Brain Injury	Population
Safiejko, K., J. Smereka, K. J. Filipiak, A. Szarpak, M. Dabrowski, J. R. Ladny, M. J. Jaguszewski and L. Szarpak	2020	Effectiveness and safety of hypotension fluid resuscitation in traumatic hemorrhagic shock: a systematic review and meta-analysis of randomized controlled trials	Population
Safiejko, K., J. Smereka, M. Pruc, J. R. Ladny, M. J. Jaguszewski, K. J. Filipiak, R. Yakubtsevich and L. Szarpak	2020	Efficacy and safety of hypertonic saline solutions fluid resuscitation on hypovolemic shock: A systematic review and meta-analysis of randomized controlled trials	Population
Schindler, P., A. Helfen, M. Wildgruber, W. Heindel, C. Schulke and M. Masthoff	2019	Intraosseous contrast administration for emergency computed tomography: A case-control study	Study type/Outcome

Study	Year	Title	Reason for exclusion
Schoeneberg, C., D. Schmitz, S. Schoeneberg, B. Hussmann and S. Lendemans	2015	Gender-specific differences in therapy and laboratory parameters and validation of mortality predictors in severely injured patients--results of a German level 1 trauma center	Study type/Outcome
Shand, S. , K. Curtis, M. Dinh and B. Burns	2019	What is the impact of prehospital blood product administration for patients with catastrophic haemorrhage: an integrative review	Study type/Outcome
Shih, A. W., S. Al Khan, A. Y. Wang, P. Dawe, P. Y. Young, A. Greene, M. Hudoba and E. Vu	2019	Systematic reviews of scores and predictors to trigger activation of massive transfusion protocols	Study type/Outcome
Singla, A., S. Kaur, N. Kaur and C. S. Gill	2016	Arterial ammonia levels: Prognostic marker in traumatic hemorrhage	Population
Smith, J. W., P. J. Matheson, G. A. Franklin, B. G. Harbrecht, J. D. Richardson and R. N. Garrison	2017	Randomized Controlled Trial Evaluating the Efficacy of Peritoneal Resuscitation in the Management of Trauma Patients Undergoing Damage Control Surgery	Intervention
Sorgjerd, R., G. A. Sunde and J. K. Heltne	2019	Comparison of two different intraosseous access methods in a physician-staffed helicopter emergency medical service - a quality assurance study	Population
Spano, P. J., 2nd, S. Shaikh, D. Boneva, S. Hai, M. McKenney and A. Elkbuli	2020	Anticoagulant chemoprophylaxis in patients with traumatic brain injuries: A systematic review	Study type/Outcome
Stansfield, R., D. Morris and E. Jesulola	2020	The Use of Tranexamic Acid (TXA) for the Management of Hemorrhage in Trauma Patients in the Prehospital Environment: Literature Review and Descriptive Analysis of Principal Themes	Study type/Outcome
Stein, P., J. D. Studt, R. Albrecht, S. Muller, D. von Ow, S. Fischer, B. Seifert, S. Mariotti, D. R. Spahn and O. M. Theusinger	2018	The Impact of Prehospital Tranexamic Acid on Blood Coagulation in Trauma Patients	Study type/Outcome
Stettler, G. R., E. E. Moore, G. R. Nunns, Ch, J. ler, E. Peltz, C. C. Silliman, A. Banerjee and A. Sauaia	2018	Rotational thromboelastometry thresholds for patients at risk for massive transfusion	Study type/Outcome
Sunde, G. A., B. E. Heradstveit, B. H. Vikenes and J. K. Heltne	2010	Emergency intraosseous access in a helicopter emergency medical service: a retrospective study	Population
Tauber, H., N. Innerhofer, D. von Langen, M. Strohle, D. Fries, M. Mittermayr, T. Hell, E. Oswald and P. Innerhofer	2020	Dynamics of Platelet Counts in Major Trauma: The Impact of Haemostatic Resuscitation and Effects of Platelet Transfusion-A Sub-Study of the Randomized Controlled RETIC Trial	not relevant to any question
Thorn, S., R. Lefering, M. Maegele, R. L. Gruen and B. Mitra	2021	Early prediction of acute traumatic coagulopathy: a validation of the COAST score using the German Trauma Registry	Intervention

Study	Year	Title	Reason for exclusion
Tisherman, S. A., H. B. Alam, P. M. Rhee, T. M. Scalea, T. Drabek, R. M. Forsythe and P. M. Kochanek	2017	Development of the emergency preservation and resuscitation for cardiac arrest from trauma clinical trial	Study type/Outcome
Tisherman, S. A., R. H. Schmicker, K. J. Brasel, E. M. Bulger, J. D. Kerby, J. P. Minei, J. L. Powell, D. A. Reiff, S. B. Rizoli and M. A. Schreiber	2015	Detailed description of all deaths in both the shock and traumatic brain injury hypertonic saline trials of the Resuscitation Outcomes Consortium	Study type/Outcome
Tonglet, M. L., J. M. Minon, L. Seidel, J. L. Poplavsky and M. Vergnion	2014	Prehospital identification of trauma patients with early acute coagulopathy and massive bleeding: results of a prospective non-interventional clinical trial evaluating the Trauma Induced Coagulopathy Clinical Score (TICCS)	Intervention
Tracy, B. M., J. R. Dunne, C. M. O'Neal and E. Clayton	2016	Venous thromboembolism prophylaxis in neurosurgical trauma patients	Population
Tran, A., J. Yates, A. Lau, J. Lampron and M. Matar	2018	Permissive hypotension versus conventional resuscitation strategies in adult trauma patients with hemorrhagic shock: A systematic review and meta-analysis of randomized controlled trials	Multiple publication without additional information
Tran, A., M. Matar, J. Lampron, E. Steyerberg, M. Taljaard and C. Vaillancourt	2018	Early identification of patients requiring massive transfusion, embolization or hemostatic surgery for traumatic hemorrhage: A systematic review and meta-analysis	Population
Valle, E. J., C. J. Allen, R. M. Van Haren, J. M. Jouria, H. Li, A. S. Livingstone, N. Namias, C. I. Schulman and K. G. Proctor	2014	Do all trauma patients benefit from tranexamic acid?	Study type/Outcome
van den Brink, D. P., M. R. Wirtz, A. S. Neto, H. Schochl, V. Viersen, J. Binnekade and N. P. Juffermans	2020	Effectiveness of prothrombin complex concentrate for the treatment of bleeding: A systematic review and meta-analysis	Study type/Outcome
Van Haren, R. M., E. J. Valle, C. M. Thorson, J. M. Jouria, A. M. Busko, G. A. Guarch, N. Namias, A. S. Livingstone and K. G. Proctor	2014	Hypercoagulability and other risk factors in trauma intensive care unit patients with venous thromboembolism	not relevant to any question
van Turenhout, E. C., S. M. Bossers, S. A. Loer, G. F. Giannakopoulos, L. A. Schwarte and P. Schober	2020	Pre-hospital transfusion of red blood cells. Part 2: A systematic review of treatment effects on outcomes	Population
van Wessem, K. J. P. and L. P. H. Leenen	2017	Thromboelastography does not provide additional information to guide resuscitation in the severely injured	Study type/Outcome
Vassallo, J., S. Horne and J. E. Smith	2014	Intraosseous access in the military operational setting	not relevant to any question
Vasudeva, M., J. K. Mathew, M. C. Fitzgerald, Z. Cheung and B. Mitra	2020	Hypocalcaemia and traumatic coagulopathy: an observational analysis	Study type/Outcome
Veigas, P. V., J. Callum, S. Rizoli, B. Nascimento and L. T. da Luz	2016	A systematic review on the rotational thrombelastometry (ROTEM R) values for the diagnosis of coagulopathy, prediction	Study type/Outcome

Study	Year	Title	Reason for exclusion
		and guidance of blood transfusion and prediction of mortality in trauma patients	
Walker, C. K., E. A. mann, T. J. Horyna and M. A. Gales	2017	Increased Enoxaparin Dosing for Venous Thromboembolism Prophylaxis in General Trauma Patients	Study type/Outcome
Warren, J., A. Moazzez, V. Chong, B. Putnam, A. Neville, G. Singer, M. Deane and D. Y. Kim	2019	Narrowed pulse pressure predicts massive transfusion and emergent operative intervention following penetrating trauma	Study type/Outcome
Weber, B. J., Kjell and C. B.	2012	The use of tranexamic acid for trauma patients?	Multiple publication without additional information
Whiting, P., M. Al, M. Westwood, I. C. Ramos, S. Ryder, N. Armstrong, K. Misso, J. Ross, J. Severens and J. Kleijnen	2015	Viscoelastic point-of-care testing to assist with the diagnosis, management and monitoring of haemostasis: a systematic review and cost-effectiveness analysis	Multiple publication without additional information
Winkelmann, M., J. D. Clausen, P. Graeff, C. Schroter, C. Zeckey, S. Weber-Spickschen and P. Mommsen	2019	Impact of Accidental Hypothermia on Pulmonary Complications in Multiply Injured Patients With Blunt Chest Trauma - A Matched-pair Analysis	Study type/Outcome
Wirtz, M. R., D. V. Schalkers, J. C. Goslings and N. P. Juffermans	2020	The impact of blood product ratio and procoagulant therapy on the development of thromboembolic events in severely injured hemorrhaging trauma patients	Study type/Outcome
Woernley, T. C., B. Maida, J. Melville, J. Marchena and N. Demian	2019	The Effect of Deep Vein Thrombosis Prophylaxis on Bleeding in Periorbital Surgery in Trauma Patients	Study type/Outcome
Wu, M. C., T. Y. Liao, E. M. Lee, Y. S. Chen, W. T. Hsu, M. G. Lee, P. Y. Tsou, S. C. Chen and C. C. Lee	2017	Administration of Hypertonic Solutions for Hemorrhagic Shock: A Systematic Review and Meta-analysis of Clinical Trials	Population
Yamamoto, K., A. Yamaguchi, M. Sawano, M. Matsuda, M. Anan, K. Inokuchi and S. Sugiyama	2016	Pre-emptive administration of fibrinogen concentrate contributes to improved prognosis in patients with severe trauma	Study type/Outcome
Yanamadala, V., B. P. Walcott, P. E. Fecci, P. Rozman, J. I. Kumar, B. V. Nahed and B. Swearingen	2014	Reversal of warfarin associated coagulopathy with 4-factor prothrombin complex concentrate in traumatic brain injury and intracranial hemorrhage	Population
Yang, X., Y. Chen, J. Li, L. Chen, H. Ren, Y. Liu and X. Zhang	2019	Hypertonic saline maintains coagulofibrinolytic homeostasis following moderate-to-severe traumatic brain injury by regulating monocyte phenotype via expression of lncRNAs	Population
Zeeshan, M., M. Khan, T. O'Keeffe, N. Pollack, M. Hamidi, N. Kulvatunyou, J. V. Sakran, L. Gries and B. Joseph	2018	Optimal timing of initiation of thromboprophylaxis in spine trauma managed operatively: A nationwide propensity-matched analysis of trauma quality improvement program	Population

Study	Year	Title	Reason for exclusion
Zehtabchi, S., S. G. Abdel Baki, L. Falzon and D. K. Nishijima	2014	Tranexamic acid for traumatic brain injury: a systematic review and meta-analysis	Population
Zhang, L. M., R. Li, X. C. Zhao, Q. Zhang and X. L. Luo	2017	Increased Transfusion of Fresh Frozen Plasma is Associated with Mortality or Worse Functional Outcomes After Severe Traumatic Brain Injury: A Retrospective Study	Study type/Outcome
Zhao, G., W. Wu, Q. M. Feng and J. Sun	2017	Evaluation of the clinical effect of small-volume resuscitation on uncontrolled hemorrhagic shock in emergency	Study type/Outcome
Ziegler, B., W. Voelckel, J. Zipperle, O. Grottke and H. Schochl	2019	Comparison between the new fully automated viscoelastic coagulation analysers TEG 6s and ROTEM Sigma in trauma patients: A prospective observational study	Study type/Outcome

Table S4. Evidence Tables

Volumen replacement therapy

Study: Reference, aim, design, setting	Participants: selection criteria, characteristics	N Participants; Intervention (IG) vs. Control group (CG)	Main outcomes	Assessment: LoE, risk of bias; Conclusions
**Heuer (2015)**<br>"Prehospital fluid management of abdominal organ trauma patients—a matched pair analysis." *Langenbeck's Archives of Surgery* 2015; 400(3): 371-379.<br>**Study design**<br>Comparative registry study<br>(TraumaRegister DGU®)<br>**Aim of the study**<br>"We conducted a retrospective matched pair analysis to assess the influence of prehospital fluid replacement volume on the clinical course of patients with solid abdominal organ trauma."<br>**Setting**<br>Germany and Austria, 1993-2009	**Inclusion criteria**<br>• Primary admission to a trauma center (no transfers)<br>• Injury Severity Score (ISS) ≥16<br>• Age ≥16 years<br>• Infusion of at least one unit of pRBCs<br>• Systolic blood pressure ≥20 mmHg at the accident site<br>• Data available for the administered prehospital fluid replacement volume, hemoglobin concentration upon hospital admission, and blood pressure at the accident site and upon hospital admission<br>**Exclusion criteria**<br>• prehospital fluid replacement volume between >1000–1500 were not included<br>**Characteristics**<br><u>Age [y], mean ± SD</u><br>IG: 34.25 (14.96) vs. CG: 36.37 (15.41), p=0.35<br><u>Male, n (%)</u><br>IG: 59 (86.8) vs. CG: 59 (86.8), p=1.0<br><u>Glasgow coma scale (GCS) mean ± SD</u>	**Participants**<br>N=136 patients<br>**Study groups**<br>IG: 0-1000 mL prehospital fluid replacement volume (N=68)<br>CG: ≥1500 mL prehospital fluid replacement volume (N=68)<br>**Co-interventions**<br>Prehospital fluid replacement: crystalloids and colloids<br>**Matching criteria**<br>• The pattern of injury for the following abdominal organs: liver, spleen, kidney, and pancreas, where matching criteria were AIS severity ≥3points<br>• The date of the injury (to account for changes in treatment over time): (I) 2002–2005, (II) 2006–2009, and (III) 2010–2012<br>• Systolic blood pressure at the accident site, which had to be at least 20mmHg: (I) 20–60mmHg, (II) 61–90mmHg, and (III) ≥91 mmHg<br>• Age: (I) 16–54, (II) 55–69, and (III) ≥70 years<br>• Intubation (yes/no)	**Outcomes after matching**<br><u>Died in hospital: n (%)</u><br>IG: 8 (11.8) vs. CG: 13 (19.1), p=0.089<br><u>Died within the first 24 h: n (%)</u><br>IG: 6 (8.8) vs. CG: 12 (17.6), p=0.129<br><u>ICU stay (days): mean ± SD</u><br>IG: 15.13 (22.32) vs. CG: 13.90 (14.32), p=0.91<br><u>Intubation at accident site: n (%)</u><br>IG: 28 (41.2) vs. 28 (41.2), p=1.0<br><u>Days intubated: mean ± SD</u><br>IG: 6.94 (8.79) vs. CG: 8.37 (10.38), p=0.5<br><u>Organ failure (%)</u><br>IG: 56.5 vs. CG: 67.4, p=0.283<br><u>Multiple organ failure</u><br>reported data unclear (no statistically significant difference between groups)<br><u>RISC (revised injury severity classification) prognosis: n (%)</u><br>IG: 10.44 (17.72) vs. CG: 13.85 (18.69), p=0.089<br><u>Days of hospitalization: mean ± SD</u><br>IG: 31.37 (28.19) vs. CG: 31.79 (27.71), p=0.99	**Level of evidence**<br>2b<br>**Risk of bias**<br>Selection bias: +<br>Performance bias: ?<br>Attrition bias: +<br>Detection bias: +<br>**Authors' conclusion**<br>"… aggressive volume replacement may lead by trend to increased mortality and could be related to early traumatic coagulopathy. The results of this study show that permissive hypotension and limited volume replacement during rescue have a positive impact on patients suffering from trauma and severe bleeding."<br>**Reviewers' conclusion**<br>The results of the study need to be interpreted with caution due to small group sizes and unclear

Study: Reference, aim, design, setting	Participants: selection criteria, characteristics	N Participants; Intervention (IG) vs. Control group (CG)	Main outcomes	Assessment: LoE, risk of bias; Conclusions
	IG: 12.46 (3.76) vs. CG 12.32 (3.67), p=0.68<br><br>Injury severity score (ISS)<br>IG: 34.97 (12.36) vs CG: 34.65 (11.17), p=0.99<br><br>Blunt abdominal trauma (%)<br>IG: 86.8 vs. CG: 90.9, p=0.45<br><br>Fluid volume replaced prehospital [ml], mean ± SD<br>IG: 818.38 (244.93), CG: 2101.84 (809.46)	• Method of rescue transport (air vs. ground transportation) Time from injury to hospital ±30 min (i.e., the difference in the time from injury to hospital in matched patients did not exceed 30 min)		performance bias. (CAVE: overlapping population with Leenen 2014)
**Hussmann (2019)**<br>"Enhanced prehospital volume therapy does not lead to improved outcomes in severely injured patients with severe traumatic brain injury ". *BMC Emergency Medicine* 2019, 19(13): 1-9<br><br>**Study design**<br>Comparative registry study<br>(TraumaRegister DGU®)<br><br>**Aim of the study**<br>The aim of this study was to investigate the influence of prehospital volume therapy on the clinical course of severely	**Inclusion criteria**<br>• patients from Germany and Austria; all of the patients were attended by a physician before hospital admission<br>• Primary admission to the hospital (no transfers)<br>• Injury Severity Score (ISS) ≥16<br>• Age ≥16 years<br>• Abbreviated Injury Scale (AIS) head ≥3<br>• Infusion of at least one unit of packed red blood cells (pRBCs)<br>• Data available for prehospital administered fluid volume, hemoglobin concentration on hospital admission, and blood pressure at the accident site<br>• and at the time of hospital admission<br><br>**Characteristics**<br>Age strata [y] (% in each group):	**Participants**<br>N=338 patients<br><br>**Study groups**<br>IG: "Low-volume"- group: ≤1000 mL (N=169)<br>CG: "High-volume"-group: ≥1501 mL (N=169)<br>prehospital administered fluid volume (crystalloids plus colloids)<br><br>**Matching criteria**<br>• AIS head 3, 4 and 5 inclusive 6<br>• Pattern of injury for the following three body regions: thorax, abdomen, and extremities, including the pelvis, where the matching criteria were AIS severity ≥3 points or <3 points; the AIS head score had to be greater than that in the other body regions<br>• To account for treatment changes that may have been established over the years, the date of injury was divided into	**Outcomes after matching**<br><br>Emergency surgery (%§):<br>IG: 6.2 vs. CG: 6.8 (p=1.0)<br><br>ICU stay (days): mean ± SD<br>IG: 16.2 (16.3) vs. CG: 14.5 (14.4) (p=0.4)<br><br>Days intubated, mean ± SD<br>IG: 11.9 (14.4) vs. CG: 11.4 (12.3) (p=0.97)<br><br>Organ failure (%§):<br>IG: 77.1 vs. CG: 83.8 (p=0.21)<br><br>Multi-organ failure (%§):<br>IG: 61.1 vs. CG: 67.7 (p=0.3)<br><br>Sepsis (%§):<br>IG: 13.5 vs. CG: 9.3 (p=0.33)<br><br>Died in hospital (%§):<br>IG: 45.6 vs. CG: 45.6 (p=1.0)	**Level of evidence**<br>2b<br><br>**Risk of bias**<br>Selection bias: ?<br>Performance bias: ?<br>Attrition bias: +<br>Detection bias: +<br><br>**Authors' conclusion**<br>"The present study does not support aggressive volume replacement after trauma and bleeding in patients with severe TBI. There were no improvements of outcome or mortality due to increased prehospital volume administration. On

Study: Reference, aim, design, setting	Participants: selection criteria, characteristics	N Participants; Intervention (IG) vs. Control group (CG)	Main outcomes	Assessment: LoE, risk of bias; Conclusions
injured patients with severe traumatic brain injury (TBI).<br><br>**Setting**<br><br>Germany and Austria, 2002-2012	16-54: 76.9<br>55-69: 8.3<br>≥70: 14.8<br><br>Male gender (% in each group)<br><br>82.2<br><br>Glasgow Coma Scale (mean ±SD)<br><br>IG: 6.6 ± 4.2<br>CG:5.8 ± 3.8 (p=0.11)<br><br>Glasgow Coma Scale ≤8 (%[§])<br><br>IG: 70.1<br>CG: 78.0 (p=0.10)<br><br>Injury Severity Score (ISS), mean ± SD<br><br>IG: 41.4 ± 13.7<br>CG: 42.3 ± 13.6 (p=0.37)<br><br>New Injury Severity Score (NISS), mean ± SD<br><br>IG: 51.1 ± 14.8<br>CG:51.9 ± 14.4 (p=0.64)<br><br>BP at accident site [mmHg], mean ± SD<br><br>IG: 121 ± 30<br>CG: 116 ± 26 (p=0.09)<br><br>[§] N=number of patients with event not reported	three groups: (1) 2002-2005, (2) 2006-2009, (3) 2010-2012.<br>• Systolic blood pressure at the accident site had to be at least 20 mmHg and was subdivided into three groups with the following values: (1) 20-60 mmHg, (2) 61-90 mmHg and (3) ≥91 mmHg<br>• Age categories were divided into three subgroups: (1) 16-54 years, (2) 55-69 years and (3) ≥70 years.<br>• Intubation (yes/no)<br>• Method of rescue transport (air vs. ground transport)<br>• Time from injury to hospital ±30 min (differences in the time from injury to hospital admission in matched patients did not exceed 10 min)<br>• Gender (male/female).<br><br>**Fluid administration, group specific data**<br><br>Fluid volume, prehospital [mL], mean ± SD<br><br>IG: 808 ± 293.5<br>CG: 2098 ± 818<br><br>Fluid volume, ED [mL], mean ± SD<br><br>IG: 3536 ± 2615<br>CG: 3116 ± 2232, p=0.12	Glasgow Outcome Scale (%[§]):<br><br>dead: IG: 46.1 vs. CG: 47.0 (p=0.7)<br>apallic: IG: 9.0 vs. 7.9 (p=0.7)<br>strongly handicapped: IG: 17.4 vs. CG: 22.6 (p=0.7)<br>mildly handicapped: IG: 17.4 vs. CG: 12.8 (p=0.7)<br>recovered well: IG: 10.2 vs. CG: 9.8 (p=0.7)<br><br>[§] N=number of patients with event not reported	the contrary, coagulation was worsened."<br><br>**Reviewers' conclusion**<br><br>The results of the study need to be interpreted with caution due to small group sizes and unclear performance bias.
**Hußmann (2015)**<br><br>"Prehospital Volume Therapy as an Independent Risk Factor after Trauma". *Biomed*	**Inclusion criteria**<br><br>• Primary admission to the hospital (no transfers).<br>• Injury Severity Score (ISS) ≥16.<br>• Age ≥16 years.	**Participants**<br><br>N=7641 patients<br><br>**Study groups**	**Overall mortality, OR (95% CI)[$]**<br><br>Full patient set<br><br>IG1: Reference<br>IG2: 0.91 (0.73–1.14)	**Level of evidence**<br><br>2b<br><br>**Risk of bias**

Study: Reference, aim, design, setting	Participants: selection criteria, characteristics	N Participants; Intervention (IG) vs. Control group (CG)	Main outcomes	Assessment: LoE, risk of bias; Conclusions
Res Int 2015; 2015: 354367.<br><br>**Study design**<br><br>Comparative registry study<br><br>(TraumaRegister DGU®)<br><br>**Aim of the study**<br><br>"The hypothesis of this study was that extensive prehospital volume replacement has a negative impact on patient mortality and represents an independent risk factor."<br><br>**Setting**<br><br>Germany and Austria, 2002-2010	• Data available for prehospital and hospital volume therapy, GCS, hemoglobin concentration, base excess, one coagulation parameter (e.g., prothrombin time), blood pressure at the accident site, blunt trauma, therapeutic measures (resuscitation, intubation, insertion of chest tube), and prehospital time.<br><br>**Characteristics**<br><br><u>Male (%)</u><br>IG1: 69.9<br>IG2: 72.1<br>IG3: 71.4<br>IG4: 74.2<br>IG5: 76.9, p<0.001<br><br><u>Age [y], mean ± SD</u><br>IG1: 52.6 ± 20.3<br>IG2: 46.3 ± 19.7<br>IG3: 43.4 ± 19.1<br>IG4: 40.1 ± 18.1<br>IG5: 39.9 ± 17.3, p<0.001<br><br><u>ISS, mean ± SD</u><br>IG1: 26.8 ± 10.9<br>IG2: 28.6 ± 12<br>IG3: 30.1 ± 12.2<br>IG4: 31.5 ± 13.3<br>IG5: 33.2 ± 13.4, p<0.001	IG1: 0–500 mL of prehospital volume therapy (N=1597)<br><br>IG2: 501–1000 mL (N=2047)<br><br>IG3: 1001–1500 mL (N=1530)<br><br>IG4: 1501–2000 mL (N=1161)<br><br>IG5: ≥2001 mL (N=1306)	IG3: 0.91 (0.71–1.12)<br>IG4: 1.10 (0.79–1.35)<br>IG5: 1.34 (1.02–1.73)<br><br><u>Patients *without* severe TBI</u><br>IG1: Reference<br>IG2: 1.44 (0.89–2.35)<br>IG3: 1.77 (1.08–2.92)<br>IG4: 2.24 (1.32–3.80)<br>IG5: 2.71 (1.62–4.52)<br><br><u>Patients *with* severe TBI</u><br>IG1: Reference<br>IG2: 0.79 (0.61–1.04)<br>IG3: 0.71 (0.52–0.94)<br>IG4: 0.82 (0.56–1.07)<br>IG5: 1.12 (0.72–1.39)<br><br>$ Stepwise multivariate regression analysis; included variables: prehospital volume replacement, in-hospital volume replacement, age, Revised Trauma Score, blood pressure at the accident site, ISS, New- ISS, AIS (head, thorax, abdomen, and extremities, including pelvis), blunt trauma, penetrating trauma, resuscitation at the accident site, time from accident to hospital admission, prehospital intubation, prehospital chest tube, base excess at admission, hemoglobin concentration at admission, cause of accident, prothrombin time in hospital, and prehospital catecholamines.	Selection bias: –<br><br>Performance bias: –<br><br>Attrition bias: +<br><br>Detection bias: +<br><br>**Authors' conclusion**<br><br>"Prehospital volume therapy in patients without severe traumatic brain injury represents an independent risk factor for mortality. In such cases, respiratory and circulatory conditions should be stabilized and permissive hypotension should be accepted, and patient transfer should not be delayed. In patients with severe traumatic brain injury, modest prehospital volume therapy can have protective effects."<br><br>**Reviewers' conclusion**<br><br>There is a substantial risk of selection and performance bias (due to imbalance of risk factors and co-interventions), partially mitigated by the chosen analysis.
**Leenen (2014)**	**Inclusion criteria**	**Participants**	<u>Days in hospital, mean ± SD</u><br>IG: 27.6 ± 27.4 vs. CG 30.4 ± 29.1, p=0.368	**Level of evidence**

Study: Reference, aim, design, setting	Participants: selection criteria, characteristics	N Participants; Intervention (IG) vs. Control group (CG)	Main outcomes	Assessment: LoE, risk of bias; Conclusions
"Limited volume resuscitation in hypotensive elderly multiple trauma is safe and prevents early clinical dilutive coagulopathy–A matched pair analysis from TraumaRegister DGU®." *Injury* 2014, 45: S59-S63.<br><br>**Study design**<br><br>Comparative registry study<br><br>(TraumaRegister DGU®)<br><br>**Aim of the study**<br><br>"The aim of the study was to examine whether preclinical administration of restrictive volume therapy in the elderly patient can be safe."<br><br>**Setting**<br><br>Hospitals primarily located in Germany (90%), data from hospitals of other countries (Austria, Belgium, China, Finland, Luxembourg, Slovenia, Switzerland, The Netherlands, and the United Arab Emirates); timeframe n.r.	• Primary admission to the hospital (no transfers).<br>• Injury Severity Score (ISS) ≥16.<br>• Age ≥60 years.<br>• Abbreviated Injury Scale Head ≤3.<br>• Systolic blood pressure at the accident site between 60 and 100 mmHg.<br>• Data available for prehospitally administered fluid volume, haemoglobin concentration on hospital admission and blood pressure at the accident site and upon hospital admission.<br><br>**Characteristics**<br><br>Age [y], mean ± SD<br><br>IG: 70.9 ± 7.7 vs. CG 70.5 ± 7.8, p=0.173<br><br>Gender male n (%)<br><br>IG: 118 (67) vs. CG 118 (67)<br><br>ISS, mean ± SD<br><br>IG: 27.9 ± 9.4 vs. CG: 28.2 ± 9.9, p=0.395<br><br>GCS, mean ± SD<br><br>IG: 12.2 ± 3.8 vs. IG: 12.4 ± 3.6, p=0.254<br><br>GCS <8, n (%)<br><br>IG: 28 (15.9) vs. CG: 28 (15.9)<br><br>Prehospital fluid volume [ml], mean ± SD<br><br>IG: 808.6 ± 241.1, CG: 1871.8 ± 570.7	N=352 patients after matching<br><br>**Study groups**<br><br>IG: 0-1000 ml prehospitally administered fluid volume [crystalloids plus colloids] (N=176)<br><br>CG: >1000 ml prehospitally administered fluid volume [crystalloids plus colloids] (N=176)<br><br>**Matching criteria**<br><br>• Pattern of injury for the following five body regions: head, thorax, abdomen, face, and extremities, including the pelvis, where matching criteria were Abbreviated Injury Scale (AIS)<br>• severity ≥3 points.<br>• Total ISS groups were matched with the following range: (1) 16–24; (2) 25–34; (3) 35–49; (4) ≥50.<br>• Systolic blood pressure at the accident site had to be between 60 and 100 mmHg and was subdivided into two groups that matched the following values: (1) 60–89 mmHg and (2) 90–100 mmHg.<br>• Ground or air transport.<br>• Gender.<br>• Intubation (yes/no)<br>• Glasgow Coma Scale (GCS).<br>• Percentage (%) of patient with GCS <8.<br>• Number of preclinical procedures (intubation, venous access, sedation, reanimation, vasopressor support).	ICU days, mean ± SD<br><br>IG: 13.2 ± 14.3 vs.C: 16.2 ± 19.2, p=0.096<br><br>Days intubated, mean ± SD<br><br>IG: 8.7 ± 11.8 vs. CG: 11.0 ± 15.9, p=0.119<br><br>Mortality, n (%)<br><br>IG: 52 ± 29.5 vs. CG: 54 ± 30.7, p=0.890<br><br>Incidence of MOF, %<br><br>IG: 41.2 vs. CG: 35.9, p=0.708	2b<br><br>**Risk of bias**<br><br>Selection bias: ?<br><br>Performance bias: ?<br><br>Attrition bias: +<br><br>Detection bias: +<br><br>**Authors' conclusion**<br><br>"In spite of some limitations such as low number of matched pairs, we draw the cautious conclusion that a restrictive preclinical volume therapy is safe and also indicated in elderly patients."<br><br>**Reviewers' conclusion**<br><br>In agreement with the authors' conclusion the results of the study need to be interpreted with caution. (CAVE: overlapping population with Heuer 2015)

Study: Reference, aim, design, setting	Participants: selection criteria, characteristics	N Participants; Intervention (IG) vs. Control group (CG)	Main outcomes	Assessment: LoE, risk of bias; Conclusions
**Schreiber (2015)**<br>“A controlled resuscitation strategy is feasible and safe in hypotensive trauma patients: results of a prospective randomized pilot trial”. *The Journal of Trauma and Acute Care Surgery*; 78(4):687-95; discussion 95-7<br>**Study design**<br>Randomised controlled trial<br>**Aim of the study**<br>to assess the feasibility and safety of controlled resuscitation (CR) for the early resuscitation of patients with traumatic shock due to blunt or penetrating mechanisms.<br>**Setting**<br>USA & Canada, 2012-2013	**Inclusion criteria**<br>• Blunt or penetrating trauma<br>• ≥15 years or estimated ≥50 kg if age was unknown<br>• out-of hospital SBP ≤90 mmHg<br>• absence of evidence of a severe head injury or a Glasgow Coma Scale score >8<br>**Exclusion criteria**<br>• receipt of >250 cc of fluid prior to randomization<br>• out-of-hospital cardiopulmonary resuscitation by EMS<br>• drowning or asphyxia due to hanging<br>• burns >20% total body surface area<br>• time of call received at dispatch to study intervention >4 hours<br>• prisoner status<br>• evidence of pregnancy<br>• ground level falls (added during the study)<br>• bilateral paralysis (added during the study)<br>CAVE: no other inclusion criteria than SBP ≤90 mmHg for shock, therefore the majority of the population has an ISS<15<br>**Characteristics**<br><u>Age, years, mean ± SD</u><br>IG: 41.9 ± 20.2<br>CG: 41.8 ± 19.2	**Participants**<br>N=292 patients<br>**Study groups**<br>IG: **c**ontrolled **r**esuscitation (N=97)<br>2x 250 cc bags of normal saline and 500 cc bottle of water, patients received 250 cc bolus of fluid only if their SBP was <70mmHg or they had no palpable radial pulse. These patients received additional 250 cc boluses to maintain a SBP of 70 mmHg or a palpable pulse as needed. If the patient had a SBP 70 mmHg and/or a palpable pulse, fluid was administered only to keep the vein open<br>CG: **s**tandard **r**esuscitation (N=95)<br>1000 cc bag of normal saline; 2 liters of fluid as an initial bolus. Following the initial bolus, additional fluid was given as needed to maintain a SBP of 110 mmHg<br>Study period: out-of-hospital enrollment until two hours into the hospital stay or until hemorrhage control was achieved	**Primary feasibility endpoint**<br><u>Early crystalloid volume, adjusted difference of the means (95% CI)[1]</u><br>**Overall:** *CG vs. IG* 0.92 (0.54 to 1.31)<br>**ISS >15:** 0 *CG vs. IG*.64 (−0.35 to 1.64)<br>**Blunt trauma**: *CG vs. IG* 0.84 (0.48 to 1.21)<br>**Penetrating trauma**: *CG vs. IG* 1.27 (0.35 to 2.20)<br>[1]adjusting variables: regional site, age, penetrating mechanism, ISS<br>**Safety endpoints**<br><u>24-h mortality n/N (%)</u><br>IG: 5/96 (5.2) vs. CG: 14/95 (14.7)<br>Adj. OR (95% CI)[2]: 0.39 (0.12-1.25)<br>[2]adjusting variables: age, penetrating mechanism, ISS<br><u>Systolic blood pressure [mmHg] at admission: mean ± SD</u><br>IG: 98.7 ± 32.5 vs. CG: 105.0 ± 34.1<br><u>Heart rate [beats/m] at admission: mean ± SD</u><br>IG: 92.9 ± 26.1 vs. CG: 86.9 ± 24.7<br><u>Glasgow Coma Scale at admission: mean ± SD</u><br>IG: 13.1 ± 3.7 vs. CG: 13.0 ± 3.8<br><u>Hemoglobin [g/dL] at admission: mean ± SD</u><br>IG: 12.3 ± 2.3 vs. CG: 12.6 ± 2.1	**Level of evidence**<br>2b ↓<br>**Risk of bias**<br>Selection bias: +<br>Performance bias: –<br>Attrition bias: +<br>Detection bias: ?<br>**Authors’ conclusion**<br>“Controlled resuscitation is feasible and safe for the initial resuscitation of hypotensive trauma patients. (…) Despite the smaller volume of fluid delivered, there were no clinically relevant differences in admission vital signs, GCS, hematologic labs or base deficit.”<br>**Reviewers’ conclusion**<br>The study was powered for the primary feasibility endpoint, early crystalloid volume (ECV), but had little power to detect all but large differences in survival. There is a risk for performance bias since the

↓ indicates that the level of evidence was lowered due to concerns related to the study design, as detailed in the Reviewers’conclusion

Study: Reference, aim, design, setting	Participants: selection criteria, characteristics	N Participants; Intervention (IG) vs. Control group (CG)	Main outcomes	Assessment: LoE, risk of bias; Conclusions
	Male sex, n (%)<br>IG: 72 (75.0)<br>CG: 74 (77.9)<br>ISS, median (IQR)<br>IG: 9.5 (1.8-19.2)<br>CG: 9 (2-24.2)<br>ISS>15, n (%)<br>IG: 33 (34.4)<br>CG: 32 (34.0)<br>Initial SBP, median (IQR), mmHg<br>IG: 81.5 (72-92)<br>CG: 84 (74.5-94)		Prothrombin time [s] at admission: mean ± SD<br>IG: 14.0 ± 2.9 vs. CG: 14.4 ± 2.7<br>Partial thromboplastin time [s] at admission: mean ± SD<br>IG: 27.4 ± 8.5 vs. CG: 32.0 ± 25.0<br>INR at admission: mean ± SD<br>IG: 1.16 ± 0.25 vs. CG: 1.18 ± 0.26<br>Platelets [10^9/L] at admission: mean ± SD<br>IG: 239.9 ± 82.4 vs. CG: 219.5 ± 61.8<br>Base deficit [mmol/L] at admission: mean ± SD<br>IG: 6.2 ± 5.7 vs. 6.4 ± 5.2<br>ICU free days at 28 days: mean ± SD<br>IG: 23.6 ± 9.8 vs. CG: 23.0 ± 10.7<br>Days out of hospital at 28 days: mean ± SD<br>IG: 18.5 ± 10.5 vs. 18.6 ± 10.3	care teams were not blinded to the interventions once patients were randomized. A large proportion of patients discontinued the intervention in both groups (20%).

Infusion solutions

Study: Reference, aim, design, setting	Participants: selection criteria, characteristics	N Participants; Intervention (IG) vs. Control group (CG)	Main outcomes	Assessment: LoE, risk of bias; Conclusions
**Rowell (2016)**<br>"The impact of pre-hospital administration of lactated Ringer's solution versus normal saline in patients with traumatic brain injury". *Journal of*	**Inclusion criteria**<br>• patients who required the highest level activation at one of 10 level 1 trauma centers<br>• subsequently received one or more units of red blood cells (RBC) within 6 hours of hospital admission<br>**Exclusion criteria**	**Participants**<br>N=308 patients with TBI<br>N=483 patients without TBI<br>**Study groups**<br>IG: lactated Ringer's (**AIS_{head} ≥3** N=52, **AIS_{head} <3** N=65)	Overall mortality at 30 days: n (%), adjusted<br>**AIS_{head} ≥3** IG vs. CG: HR 1.78, CI 1.04–3.04, p=0.035<br>**AIS_{head} <3** IG vs. CG: HR 1.49, CI 0.757–2.95, p=0.247<br>Overall mortality at 30 days: (%), unadjusted	**Level of evidence**<br>2b<br>**Risk of bias**<br>Selection bias: –<br>Performance bias: –<br>Attrition bias: +

Study: Reference, aim, design, setting	Participants: selection criteria, characteristics	N Participants; Intervention (IG) vs. Control group (CG)	Main outcomes	Assessment: LoE, risk of bias; Conclusions
Neurotrauma; 33(11): 1054-1059.<br>**Study design**<br>secondary analysis of a prospective cohort study (PROMMTT)<br>**Aim of the study**<br>The purpose of this study was to compare the effects of pre-hospital administration of lactated Ringer's (LR) and normal saline (NS) on outcomes in patients with and without significant traumatic brain injury (TBI).<br>**Setting**<br>USA, date n.r.	• age <16 years<br>• transfer from another hospital<br>• pregnancy<br>• >20% burn injury<br>• inhalation injury<br>• incarceration<br>• death within 30 minutes of hospital admission<br>• received any other type of fluid or blood products and those who received both LR and NS<br>• Patients receiving a small volume of pre-hospital fluid (<200 mL) and those with minor injuries (ISS <9)<br>**Characteristics**<br><u>Injury Severity Score, median</u><br>**AIS_{head} ≥3** IG: 42 (32-57) vs. CG 34 (26-43), p<0.001<br>**AIS_{head} <3** IG: 26 (17–35) vs. CG 19 (13–29), p<0.001<br><u>Abbreviated Injury Scale head, median</u><br>**AIS_{head} ≥3** IG: 5 (4-5) vs. CG 4 (3-5), p<0.001<br>**AIS_{head} <3** IG: 0 (0–1) vs. CG 0 (0–0), p<0.001<br><u>Age [y], mean ± SD</u><br>**AIS_{head} ≥3** IG: 45.2 ± 20.8 vs. IG 42.8 ± 18.4, p=0.453<br>**AIS_{head} <3** IG: 41.1 ± 20.1 vs. CG 38.8 ± 17.8, p=0.492<br><u>Male n (%)</u>	CG: normal saline (**AIS_{head} ≥3** N=256, **AIS_{head} <3** N=418)	**AIS_{head} ≥3** IG: 50 vs. CG: 28<br>**AIS_{head} <3** IG: 25 vs. CG: 11<br>**Secondary outcomes for patients with AIS_{head} ≥3[§]**<br><u>Systolic blood pressure [mmHg][*]</u><br>**AIS_{head} ≥3** IG: 120 ± 32 vs. CG: 107 ± 29, p=0.264<br>**AIS_{head} <3** IG: 118 ± 28 vs. CG: 104 ± 28, p=0.271<br><u>Heart rate[*]</u><br>**AIS_{head} ≥3** IG: 105 ± 30 vs. CG: 103 ± 28, p=0.827<br>**AIS_{head} <3** IG: 112 ± 26 vs. CG: 107 ± 2827, p=0.392<br><u>INR</u><br>**AIS_{head} ≥3** IG: 1.9 ± 1.7 vs. CG: 1.4 ± 1.1, p=0.559<br>**AIS_{head} <3** IG: 1.6 ± 0.7 vs. CG: 1.4 ± 1.3, p=0.976<br>Base deficit<br>**AIS_{head} ≥3** IG: 6.5 ± 5.0 vs. CG: 7.3 ± 5.7, p=0.406<br>**AIS_{head} <3** IG: 7.7 ± 6.1 vs. CG: 7.0 ± 5.4, p=0.401<br><u>pH</u><br>**AIS_{head} ≥3** IG: 7.26 ± 0.14 vs. CG: 7.24 ± 0.14, p=0.142<br>**AIS_{head} <3** IG: 7.24 ± 0.17 vs. CG: 7.25 ± 0.13, p=0.921<br><u>Hemoglobin (g/dL)</u><br>**AIS_{head} ≥3** IG: 10.5 ± 2.6 vs. CG: 11.7 ± 2.1, p=0.113<br>**AIS_{head} <3** IG: 10.5 ± 2.8 vs. CG: 11.6 ± 2.2, p=0.998<br><u>Lactate (mEq/L)</u><br>**AIS_{head} ≥3** IG: 5.7 ± 3.5 vs. CG: 4.5 ± 2.8, p=0.753<br>**AIS_{head} <3** IG: 4.8 ± 3.0 vs. CG: 7.1 ± 4.9, p=0.100	Detection bias: ?<br>**Authors' conclusion**<br>"Administration of pre-hospital LR was associated with increased adjusted mortality compared with NS in patients with significant TBI. These findings justify the need for a randomized clinical trial comparing pre-hospital administration of LR and NS in patients with TBI."<br>**Reviewers' conclusion**<br>The intervention and control groups were not balanced with respect to several important baseline characteristics and co-interventions. However, the analyses were adjusted for this imbalance.

Study: Reference, aim, design, setting	Participants: selection criteria, characteristics	N Participants; Intervention (IG) vs. Control group (CG)	Main outcomes	Assessment: LoE, risk of bias; Conclusions
	**AIS_{head} ≥3** IG: 34 (65.4) vs. CG 184 (71.9), p=0.348<br>**AIS_{head} <3** IG: 73.8 vs. CG 77.3, p=0.543<br><br><u>Mechanism blunt (%)</u><br>**AIS_{head} ≥3** IG: 44 (84.6) vs. CG: 215 (84.0), p=0.509<br>**AIS_{head} <3** IG: 56.9 vs. CG 56.5, p=0.944<br><br><u>Mechanism penetrating n (%)</u><br>**AIS_{head} ≥3** IG: 8 (15.4) vs. CG: 41 (16.0)<br>**AIS_{head} <3** IG: 43.1 vs. CG 43.5, p –<br><br><u>Prehospital intubation n (%)</u><br>**AIS_{head} ≥3** IG: 56 (92.3) vs. CG: 146 (57), p<0.001<br>**AIS_{head} <3** IG: 70.8 vs. CG 20.7, p<0.001<br><u>Prehospital transport time (min), mean ± SD</u><br>**AIS_{head} ≥3** IG: 57 ± 21 vs. CG: 69±62, p=0.599<br>**AIS_{head} <3** IG: 57 ± 34 vs. CG 51 ± 29, p=0.283<br><br><u>Prehospital fluid volume (ml), mean ± SD</u><br>**AIS_{head} ≥3** IG: 1540 ± 1030 vs. CG: 970 ± 770, p<0.001<br>**AIS_{head} <3** IG: 1520 ± 1280 vs. CG 890 ± 650, p<0.001		<u>6-h fluid requirement (L)</u><br>**AIS_{head} ≥3** IG: 5.4 ± 2.9 vs. CG: 3.8 ± 3.1, p=0.256<br>**AIS_{head} <3** IG: 6.7 ± 4.1 vs. CG: 4.1 ± 3.3, p=0.368<br><br><u>6-h RBC requirement (units)</u><br>**AIS_{head} ≥3** IG: 4.5 ± 3.7 vs. CG: 5.8 ± 7.0, p=0.161<br>**AIS_{head} <3** IG: 8.3 ± 8.7 vs. CG: 7.3 ± 9.7, p=0.287<br><br>[§] mean ± SD ? (not reported)<br><br>[*]SBP and HR: Initial value measured on arrival to the emergency department	

Hypertonic solutions

Study: Reference, aim, design, setting	Participants: selection criteria, characteristics	N Participants; Intervention (IG) vs. Control group (CG)	Main outcomes	Assessment: LoE, risk of bias; Conclusions
**Delano (2015)**<br>"Prehospital resuscitation of traumatic hemorrhagic shock with hypertonic solutions worsens hypocoagulation and hyperfibrinolysis." *Shock* 44(1): 25.<br>**Study design**<br>*a priori* subgroup analysis of a randomized controlled, trial (Bulger 2011)<br>**Aim of the study**<br>"This study explores the impact of resuscitation with various hypertonic solutions on early coagulopathy after trauma. As part of a larger prospective clinical trial evaluating prehospital resuscitation of severely injured trauma patients in hypovolemic shock, the aim of this ancillary laboratory study was to determine the impact of a single-bolus (250 mL) infusion of hypertonic fluids on the risk of acute traumatic coagulopathy."	**Inclusion criteria**<br>• patients in hypovolemic shock enrolled in two centers (Toronto and Seattle)<br>• had additional laboratory tests done during the initial 24 h of hospitalization.<br>• hypovolemic shock defined as out-of-hospital systolic blood pressure (SBP) of 70 mmHg or less or SBP 71 to 90 mmHg with a heart rate of 108 beats/min or more.<br>**Exclusion criteria**<br>• pregnancy,<br>• younger than 15 years,<br>• more than 2,000 mL of intravenous fluids or blood before enrollment,<br>• hypothermia (<28°C),<br>• drowning,<br>• asphyxia,<br>• burns,<br>• isolated penetrating head injury,<br>• time of call received by dispatch to study intervention longer than 4 h,<br>• known prisoners,<br>• and transfer from another hospital<br>**Characteristics**<br><u>Age, years mean ± SD</u><br>IG1: 43.2 (22.8)<br>IG2: 42.9 (18.5)<br>CG: 42 (21)	**Participants**<br>N=34 patients<br>**Study groups**<br>IG1: out-of-hospital single bolus of 250 mL of 7.5% hypertonic saline (HS) (N=9)<br>IG2: out-of-hospital single bolus of 250 mL of 7.5% hypertonic saline/6% Dextran 70 (HSD) (N=8)<br>CG: out-of-hospital single bolus of 250 mL of standard 0.9% normal saline(NS) (N=17)<br>**Co-interventions**<br>Additional fluids were allowed after study fluid, as guided by local protocols	<u>Admission SBP, mmHg mean ± SD</u><br>IG1: 141 (30.7) vs. CG 112.1 (26.8), p<0.05<br>IG2: 126 (24.6) vs. CG 112.1 (26.8), not significant<br>**Admission biochemistry and coagulation**<br><u>Base deficit, mEq/L, mean (SEM)</u><br>IG1: −9.2 (5.6) vs. CG: −9.4 (6.7), not significant<br>IG2: −13.1 (12.2) vs. CG: −9.4 (6.7), not significant<br><u>Lactate, mmol/l, mean (SEM)</u><br>IG1: 5.3 (3.5) vs. CG: 5.3 (4.3), not significant<br>IG2: 4.8 (1.1) vs. CG: 5.3 (4.3), not significant<br><u>International normalized ratio (INR), mean (SEM)</u><br>IG1: 1.3 (0.3) vs. CG: 1.2 (0.1), not significant<br>IG2:1.6 (1.2) vs. CG: 1.2 (0.1), not significant<br><u>Coagulopathic, n (%)</u><br>IG1: 5 (55.6) vs. CG: 8 (47.1), not significant<br>IG2: 5 (62.5) vs. CG: 8 (47.1), not significant<br><u>INR ≥1.3, n (%)</u><br>IG1: 3 (33.3) vs. CG: 7 (41.2), not significant<br>IG2: 4 (50.0) vs. CG: 7 (41.2), not significant<br>**Coagulation marker at admission**<br><u>Thrombin-antithrombin complex (TAT) [1–4 ng/mL], mean (SEM)</u><br>IG1: 27.2 (1.9) vs. IG2: 14.6 (2.1), not significant<br>IG1: 27.2 (1.9) vs. CG: 39.6 (3.1), not significant<br>IG2: 14.6 (2.1 vs. CG: 39.6 (3.1), p<0.05	**Level of evidence**<br>2b↓<br>**Risk of bias**<br>Selection bias: ?<br>Performance bias: ?<br>Attrition bias: +<br>Detection bias: +<br>**Authors' conclusion**<br>"In conclusion, hypertonic solutions, particularly when combined with Dextran, seem to worsen the hypocoagulability and hyperfibrinolysis that occur after hemorrhagic shock in the cohort of trauma patients evaluated in our study. Although HS and HSD increased the SBP above that of NS, they may not have corrected the shock because acidosis persisted despite the BP normalization."<br>**Reviewers' conclusion**<br>Small group sizes may limit the interpretation of the results. Surrogate outcomes are reported, but

Study: Reference, aim, design, setting	Participants: selection criteria, characteristics	N Participants; Intervention (IG) vs. Control group (CG)	Main outcomes	Assessment: LoE, risk of bias; Conclusions
**Setting**<br>USA, Canada	Male sex, n (%)<br>IG1: 8 (88.9)<br>IG2: 5 (62.5)<br>CG:12 (70.6)<br>ISS, mean ± SD<br>IG1: 24.5 (16.8)<br>IG2: 21.3 (9)<br>CG: 21.2 (10.6)		All groups vs. age-matched healthy controls $p<0.05$<br>Tissue factor (TF) [ND], mean (SEM)<br>IG1: 109.6 (14.2) vs. IG2: 70.3 (10.4), not significant<br>IG1: 109.6 (14.2) vs. CG: 192.6 (29), not significant<br>IG2: 70.3 (10.4) vs. CG: 192.6 (29), $p<0.05$<br>IG2 vs. age-matched healthy controls $p<0.05$<br>Tissue factor pathway inhibitor [7.5–41.2 ng/mL], mean (SEM)<br>IG1: 24.6 (3.1) vs. IG2: 28.8 (3.7), not significant<br>IG1: 24.6 (3.1) vs. CG: 22.1 (2.5), not significant<br>IG2: 28.8 (3.7) vs. CG: 22.1 (2.5), not significant<br>IG2 vs. age-matched healthy controls $p<0.05$<br>Thrombomodulin [2.7–5.4 ng/mL], mean (SEM)<br>IG1: 4.8 (0.6) vs. IG2: 5.2 (0.9), not significant<br>IG1: 4.8 (0.6) vs. CG: 6.5 (1.2) , not significant<br>IG2: 5.2 (0.9) vs. CG: 6.5 (1.2), not significant<br>**Fibrinolysis marker at admission**<br>Tissue plasminogen activator (tPA) [3–12 ng/mL], mean (SEM)<br>IG1: 19.7 (2.5) vs. IG2: 25.2 (3.1), not significant<br>IG1: 19.7 (2.5) vs. CG: 15.4 (1.9), not significant<br>IG2: 25.2 (3.1) vs. CG: 15.4 (1.9), $p<0.05$<br>All groups vs. age-matched healthy controls $p<0.05$<br>Plasminogen activator inhibitor type 1 (PAI-1) [4–40 ng/mL] mean (SEM)	no patient-relevant outcomes.

Study: Reference, aim, design, setting	Participants: selection criteria, characteristics	N Participants; Intervention (IG) vs. Control group (CG)	Main outcomes	Assessment: LoE, risk of bias; Conclusions
			IG1: 38.7 (4.1) vs. IG2: 19.6 (3.4), not significant<br>IG1: 38.7 (4.1) vs. CG: 46.3 (6.5), not significant<br>IG2: 19.6 (3.4) vs. CG: 46.3 (6.5), p<0.05<br><br>All groups vs. age-matched healthy controls p<0.05<br><br><u>Thrombin-activatable fibrinolysis inhibitor (TAFI) [40%–250%], mean (SEM)</u><br>IG1: 95.2 (10.2) vs. IG2: 80.5 (11.4), not significant<br>IG1: 95.2 (10.2) vs. CG: 104.6 (6.3), not significant<br>IG2: 80.5 (11.4) vs. CG: 104.6 (6.3), p<0.05<br><br>IG2 vs. age-matched healthy controls p<0.05<br><br><u>D-dimer [0–400 ng/mL], mean (SEM)</u><br>IG1: 8.023 (1.205) vs. IG2: 10.786 (1.359), p<0.05<br>IG1: 8.023 (1.205) vs. CG: 6.375 (997), p<0.05<br>IG2: 10.786 (.,359) vs. CG: 6,375 (997), p<0.05<br><br>All groups vs. age-matched healthy controls p<0.05	
**Han (2015)**<br>"Comparison of 3% And 7.5% Hypertonic Saline in Resuscitation after Traumatic Hypovolemic Shock". *Shock* 43(3), 244-249<br>**Study design**<br>Randomised controlled trial<br>**Aim of the study**<br>The aim of this study was to evaluate the	**Inclusion criteria**<br>• trauma victims<br>• prehospital systolic blood pressure (SBP) of ≤70 mmHg<br>• or SBP 70 to 90 mmHg and heart rate (HR) ≥108 beats/min<br>• aged 15 years or older<br>**Exclusion criteria**<br>• younger than 15 years<br>• injury during previous 4 h<br>• hypothermia (<28°C)<br>• administration of dopamine or other vasoactive agents	**Patients**<br>N=246 patients<br>**Study groups**<br>IG1: 3% HSS (Hypertonic saline solution) (N=82)<br><br>IG2: 7.5% HSS (Hypertonic saline solution) (N=80)<br><br>CG: LRS (standard fluid, Lactated Ringer's solution) (N=84)	<u>Mortality, n (%)</u><br>Total deaths: 37 (15.4)<br><br>Deaths within first 24 h: 30 (81.1)<br><br>24-h survival in IG1 and IG2 better than CG, but no statistically significant difference<br>**Postinfusion complications, n (%)**<br><u>Tachycardia</u><br>IG1: 5 (6.1)<br>IG2: 22 (27.5) (vs. IG1 and CG, p<0.05)<br>CG: 4 (4.8)<br><br><u>Coagulopathy</u>	**Level of evidence**<br>1b<br>**Risk of bias**<br>Selection bias: +<br><br>Performance bias: +<br><br>Attrition bias: ?<br><br>Detection bias: ?<br>**Authors' conclusion**<br>"In summary, administration of 3% HSS offered hemodynamic

Study: Reference, aim, design, setting	Participants: selection criteria, characteristics	N Participants; Intervention (IG) vs. Control group (CG)	Main outcomes	Assessment: LoE, risk of bias; Conclusions
resuscitative effects and safety of 3% Hypertonic saline solution (HSS) and to compare the risks of complications caused by HSS and standard fluid treatments.<br><br>**Setting**<br><br>China, 2008-2012	• administration of more than 2,000 mL of crystalloid before the study fluid<br>• ongoing cardiopulmonary resuscitation<br>• severe cardio-respiratory dysfunction<br>• known or suspected pregnancy<br>• traumatic brain injury (TBI)<br>• death within 1 h after intervention<br><br>**Characteristics**<br><br><u>Age, years (mean ± SD)</u><br>IG1: 45 ± 0.5<br>IG2: 48 ± 3.1<br>CG: 43 ± 9.5<br><br><u>Sex (male, n (%))</u><br>IG1: 61 (74.4)<br>IG2: 65 (81.3)<br>CG: 63 (75.0)<br><br><u>ISS (mean ± SD)</u><br>IG1: 18.5 ± 2.5<br>IG2: 15.6 ± 3.1<br>CG: 16.5 ± 3.4<br><br><u>Shock index (mean ± SD)</u><br>IG1: 1.5 ± 0.2<br>IG2: 1.6 ± 0.3<br>CG: 1.5 ± 0.2<br><br><u>Preinfusion MAP, mmHg (mean ± SD)</u><br>IG1: 49 ± 6.6<br>IG2: 51 ± 9.7<br>CG: 52 ± 4.7<br><br><u>Infusion volume, 1 h, L (mean ± SD)</u>		IG1: 0<br>IG2: 2 (2.5)<br>CG: 9 (10.7) (vs. IG1 and IG2, $p<0.001$)<br><br><u>Acute renal failure</u><br>IG1: 0<br>IG2: 0<br>CG: 5 (6.0) (vs. IG1 and IG2, $p<0.001$)<br><br><u>Pulmonary edema</u><br>IG1: 0<br>IG2: 0<br>CG: 4 (4.8) vs. IG1 and IG2, $p<0.001$)<br><br><u>Heart failure</u><br>IG1: 1 (1.2)<br>IG2: 1 (1.3)<br>CG: 2 (2.4)<br><br><u>Transient Hypotension</u><br>IG1: 0<br>IG2: 4 (5.0) (vs. IG1 and CG $p<0.05$)<br>CG: 0<br><br><u>ARDS</u><br>IG1: 1 (1.2)<br>IG2: 1 (1.3)<br>CG: 3 (3.6)<br><br><u>MODS</u><br>IG1: 2 (2.4)<br>IG2: 1 (1.3)<br>CG: 3 (3.6)	benefits equivalent to those of 7.5% HSS infusion with lower degrees of hypernatremia and hyperchloremia and lower risks of cardiac dysrhythmia and transient hypotension. In addition, higher incidences of pulmonary edema, renal failure, and coagulopathy occurred in the LRS group.”<br><br>**Reviewers’ conclusion**<br><br>There might be a risk of attrition bias as information regarding length of follow-up regarding adverse events and the availability of outcome data are lacking.

Study: Reference, aim, design, setting	Participants: selection criteria, characteristics	N Participants; Intervention (IG) vs. Control group (CG)	Main outcomes	Assessment: LoE, risk of bias; Conclusions
	IG1: 1.1 ± 0.2<br>IG2: 1.0 ± 0.2<br>CG: 2.1 ± 0.3 (CG vs. IG1 and IG2, p<0.05)			

Prehospital transfusion

Study: Reference, aim, design, setting	Participants: selection criteria, characteristics	N Participants; Intervention (IG) vs. Control group (CG)	Main outcomes	Assessment: LoE, risk of bias; Conclusions
**Brown (2015)**<br>"Pretrauma Center Red Blood Cell Transfusion Is Associated With Reduced Mortality and Coagulopathy in Severely Injured Patients With Blunt Trauma". *Annals of Surgery* 261(5): 997–1005.<br>**Study design**<br>Secondary analysis of a prospective cohort study<br>(Host Response to Injury Collaborative)<br>**Aim of the study**<br>The aim of the study was to evaluate the association of PTC RBC (Pretrauma Center Red Blood Cell) transfusion with mortality and trauma-induced coagulopathy (TIC) in	**Inclusion criteria**<br>• Blunt injured patients in shock<br>• Arriving at the study trauma center within 2 hours of injury<br>**Characteristics (matched cohort)**<br><u>Age (median, IQR)</u><br>IG: 36 (28 - 52)<br>CG: 37 (24 - 55) (p=0.63)<br><u>Sex (male, %)</u><br>IG: 60<br>CG: 72 (p=0.28)<br><u>ISS (median, IQR)</u><br>IG: 34 (18 - 43)<br>CG: 30 (23 - 43) (p=0.81)<br><u>Initial haemoglobin [g/dL], median, IQR)</u><br>IG: 11.0 (8.8 - 13)<br>CG: 11.1 (9.3 - 12.6) (p=0.90)<br><u>PTC hypotension§ (%)</u><br>IG: 71<br>CG: 53 (p=0.14)<br><u>Admission hypotension§ (%)</u>	**Participants**<br>N=113 patients, 3:1 matching<br>**Study groups**<br>IG: PTC RBC (N=35)<br>transfusion of RBCs at any time before the subject's arrival at the study trauma center<br>CG: No PTC RBC (N=78)	<u>Mortality:</u><br>24-hour mortality<br>PTC RBC is independently associated with a 98% reduction in odds of 24-hour mortality: OR = 0.02; 95% CI, 0.01 - 0.69 (p=0.04)<br><u>30-day mortality:</u><br>PTC RBC is independently associated with a 88% reduction in the risk of 30-day-mortality:<br>HR = 0.12; 95% CI, 0.03 - 0.61 (p=0.01)<br><u>Trauma-Induced Coagulopathy (TIC):</u><br>PTC RBC is independently associated with a 99% reduction in odds of TIC: OR = 0.01; 95% CI, 0.01 - 0.95 (p=0.05)<br><br>CAVE: Results regarding the matched cohort only	**Level of evidence**<br>3b↓<br>**Risk of bias**<br>Selection bias: ?<br>Performance bias: ?<br>Attrition bias: +<br>Detection bias: +<br>**Authors' conclusion**<br>"PTC RBC administration was associated with a lower risk of 24-hour mortality, 30-day mortality, and TIC in severely injured patients with blunt trauma, warranting further prospective study."<br>**Reviewers' conclusion**<br>The analysis has some limitations as the original cohort study was not designed to address the specific question. There

Study: Reference, aim, design, setting	Participants: selection criteria, characteristics	N Participants; Intervention (IG) vs. Control group (CG)	Main outcomes	Assessment: LoE, risk of bias; Conclusions
severely injured patients with blunt trauma<br><br>**Setting**<br><br>United States, 2003-2010	IG: 60<br>CG: 74 (p=0.02)<br><br><u>24-h RBC trauma center [units], median (IQR)</u><br>IG: 14.0 (7.0 - 21.7)<br>CG: 8.3 (3.4 - 18.5) (p=0.03)<br><br>§ N=number of patients with event not reported			might be a performance bias as pretrauma center care was not standardized.
**Gruen (2020)**<br><br>"Association of Prehospital Plasma With Survival in Patients With Traumatic Brain Injury A Secondary Analysis of the PAMPer Cluster Randomized Clinical Trial". *JAMA Network Open* 3(10), 1-15.<br><br>**Study design**<br><br>Predefined subgroup analysis of a randomised controlled trial<br><br>(PAMPer trial)<br><br>**Aim of the study**<br><br>The aim of the study was to characterize the survival benefit associated with prehospital plasma among patients with traumatic brain injury (TBI) using data derived	**Inclusion criteria**<br><br>• Same as in original PAMPer trial (Sperry, 2018, see below)<br>• For subgroup analysis:<br>• Patients with TBI (assessed imaging results and defined TBI as brain injury documented by CT scan. TBI was defined as any finding consistent with TBI as defined by a radiologist at initial head CT)<br><br>**Exclusion criteria**<br><br>• Same as in original PAMPer trial (Sperry, 2018)<br><br>**Characteristics**<br><br><u>Age [y], median (IQR)</u><br>IG: 42.50 (25.25 - 60.25)<br>CG: 44.00 (25.00 - 59.25) (p=0.80)<br><br><u>Sex (male), n (%)</u><br>IG: 50 (67.6)<br>CG: 75 (81.5) (p=0.06)	**Participants**<br><br>N=166 TBI patients (predefined subgroup)<br><br>**Study groups**<br><br>IG: Plasma (2 units of thawed plasma followed by standard care fluid resuscitation) (N=74)<br><br>CG: Standard Care (crystalloid or crystalloid and packed red blood cells) (N=92)	<u>Mortality:</u><br>30-d mortality (n (%))<br><br>IG: 26 (35.1) vs. CG: 51 (55.4), p=0.01<br><br><u>24-hour mortality (n (%))</u><br>IG: 12 (16.2) vs. CG: 33 (35.9), p=0.008<br>Analysis of 30-d survival, Hazard Ratio for patients with TBI (HR, 95% CI)*<br><br>IG vs. CG: HR 0.55 (0.33-0.94) (p=0.03)<br><br>**Others**<br><br><u>Multiple organ failure, n (%)</u><br>IG: 57 (77.0) vs. CG: 52 (56.5), p=0.009<br><br><u>Intensive care unit length of stay [d], median (IQR)</u><br>IG: 8 (3 - 15) vs. CG: 6 (1 - 12), p=0.04<br><br><u>Hospital length of stay [d], median (IQR)</u><br>IG: 13 (6 - 20) vs. CG: 8 (1 - 19), p=0.05<br><br><u>Ventilator duration [d], median (IQR)</u>	**Level of evidence**<br><br>1b<br><br>**Risk of bias**<br><br>Selection bias: +<br><br>Performance bias: –<br><br>Attrition bias: +<br><br>Detection bias: +<br><br>**Authors' conclusion**<br><br>"Early administration of prehospital plasma to patients with TBI is associated with improved survival, particularly among those with polytrauma and risk of hemorrhagic shock. Future studies are needed to confirm the clinical benefits of early plasma resuscitation. Our results are exploratory, but the prehospital setting may be a critical time to intervene

Study: Reference, aim, design, setting	Participants: selection criteria, characteristics	N Participants; Intervention (IG) vs. Control group (CG)	Main outcomes	Assessment: LoE, risk of bias; Conclusions
from a recently completed prehospital plasma clinical trial.<br><br>**Setting**<br><br>United States, 2014 – 2017 (Same as in original PAMPer trial (Sperry, 2018))	<u>ISS, median (IQR)</u><br>IG: 29 (20 - 41)<br>CG: 29 (22 - 37) (p=0.92)<br><br><u>Prehospital GCS <8, n (%)</u><br>IG: 52 (70.3)<br>CG: 71 (79.8)<br><br><u>Prehospital GCS 8-12, n (%)</u><br>IG: 7 (9.5)<br>CG: 6 (6.7)<br><br><u>Prehospital GCS 13-15, n (%)</u><br>IG: 15 (20.3)<br>CG: 12 (13.5)<br><br><u>Blunt injury, (n (%)</u><br>IG: 74 (100.0)<br>CG: 92 (100.0)<br><br><u>Crystalloid fluid, mL (median (IQR))</u><br>IG: 700.00 (0.00 - 1287.50)<br>CG: 1000.00 (0.00 - 1500.00) (p=0.20)<br><br><u>INR (median, IQR)</u><br>IG: 1.20 (1.10 - 1.40)<br>CG: 1.40 (1.20 - 1.80) (p=0.001)		IG: 5 (2 - 11) CG: 4 (1 - 9), p=0.12<br><br>* After adjustment for multiple confounders and assessment of the degree of brain injury with clinical variables and biomarker	in the care of patients with TBI."<br><br>**Reviewers' conclusion**<br><br>There is a risk of performance bias due to lack of blinding of study staff and variability in prehospital and hospital care.
**Guyette (2021)**<br><br>"Prehospital Blood Product and Crystalloid Resuscitation in the Severely Injured Patient A Secondary Analysis of the Prehospital Air Medical	**Inclusion criteria**<br><br>• Same as in original PAMPer trial (Sperry, 2018, see below)<br><br>**Exclusion criteria**<br><br>• Patients transferred from referring facilities from the originally reported trial population (excluded, as they	**Participants**<br><br>N=407 patients<br><br>**Study groups**<br><br>PRBC+Plasma (N=38, 10%)<br><br>Plasma (N=147, 36%)<br><br>PRBC only (N=83, 20%)	<u>30-day Mortality, n (%), unadjusted (p=0.05)</u><br>PRBC+Plasma: 10 (26)<br>Plasma: 31 (23)<br>PRBC only: 30 (36)<br>Crystalloid only: 47 (37)<br><br>**Cause of death, unadjusted (overall p=0.18)**<br><br><u>Hemorrhage, n (%)</u>	**Level of evidence**<br><br>2b<br><br>**Risk of bias**<br><br>Selection bias: ?<br><br>Performance bias: –<br><br>Attrition bias: +

Study: Reference, aim, design, setting	Participants: selection criteria, characteristics	N Participants; Intervention (IG) vs. Control group (CG)	Main outcomes	Assessment: LoE, risk of bias; Conclusions
Plasma Trial". *Annals of Surgery* 273(2), 358-364.<br><br>**Study design**<br>Comparative registry study:<br>secondary analysis of a randomised controlled trial (PAMPer trial)<br><br>**Aim of the study**<br>The aim of the study was to determine whether prehospital blood products reduce 30-day mortality in patients at risk for hemorrhagic shock compared with crystalloid only resuscitation.<br><br>**Setting**<br>United States, 2014 – 2017 (Same as in original PAMPer trial (Sperry, 2018))	could receive both crystalloid and blood products at the outside facility before HEMS transport and potentially bias the outcomes of the prehospital resuscitation groups)<br><br>**Characteristics**<br><br><u>Age (median, IQR)</u><br>PRBC (prehospital red blood cells)+Plasma: 45 (32 - 58)<br>Plasma: 43 (30 - 55)<br>PRBC only: 40 (25 - 55)<br>Crystalloid only: 47 (28 - 65), p=0.25<br><br><u>Sex (male), n (%)</u><br>PRBC+Plasma: 26 (68.4)<br>Plasma: 108 (73.5)<br>PRBC only: 64 (77.1)<br>Crystalloid only: 100 (71.9), p=0.75<br><br><u>Prehospital SBP [mmHg], median (IQR)</u><br>PRBC+Plasma: 69 (61 - 82)<br>Plasma: 72.5 (63 - 81)<br>PRBC only: 69 (60 - 80)<br>Crystalloid only: 72 (63 - 81), p=0.79<br><br><u>Prehospital GCS (median, IQR)</u><br>PRBC+Plasma: 12.5 (3 - 15)<br>Plasma: 12 (3 - 15)<br>PRBC only: 13 (3 - 15)<br>Crystalloid only: 7 (3 - 15), p=0.56<br><br><u>ISS (median, IQR)</u><br>PRBC+Plasma: 24 (17 - 34)<br>Plasma: 22 (14 - 34)	Crystalloid only (N=139, 34%)	PRBC+Plasma: 4 (40)<br>Plasma: 9 (29)<br>PRBC only: 15 (50)<br>Crystalloid only: 10 (21)<br><br><u>Traumatic Brain Injury, n (%)</u><br>PRBC+Plasma: 2 (20)<br>Plasma: 8 (26)<br>PRBC only: 9 (30)<br>Crystalloid only: 17 (36)<br><br><u>Cox proportional Hazard Regression Treatment Effect Estimates (HR (95% CI)*</u><br>PRBC+Plasma: 0.38 (0.26 – 0.55) (p<0.001)<br>Plasma: 0.57 (0.36 – 0.91) (p=0.017)<br>PRBC: 0.68 (0.49 – 0.95) (p=0.025)<br>Crystalloid only: Reference<br><br>* Adjusted for age, injury, ISS, severe prehospital hypotension (SBP <70 mm Hg), prehospital time, prehospital crystalloid volume, emergent procedure within 24hours of admission, INR, 24-hour total PRBC and plasma transfusion requirements, development of multiple organ failure, nosocomial infection, AIS for head, chest, and abdomen.	Detection bias: +<br><br>**Authors' conclusion**<br>"Patients receiving prehospital PRBC+plasma had the greatest mortality benefit. Crystalloid only had the worst survival. Patients with hemorrhagic shock should receive prehospital blood products when available, preferably PRBC+plasma. Prehospital whole blood may be ideal in this population."<br><br>**Reviewers' conclusion**<br>There is a risk of performance bias due to lack of blinding of study staff and variability in prehospital and hospital care.<br>Further, as the study was not designed to address the specific question, the results should be interpreted with caution.

Study: Reference, aim, design, setting	Participants: selection criteria, characteristics	N Participants; Intervention (IG) vs. Control group (CG)	Main outcomes	Assessment: LoE, risk of bias; Conclusions
	PRBC only: 22 (16 - 29)<br>Crystalloid only: 21 (12 - 33), p=0.57			
**Henriksen (2016)**<br>"Pre-hospital transfusion of plasma in hemorrhaging trauma patients independently improves hemostatic competence and acidosis". *Scandinavian Journal of Trauma, Resuscitation and Emergency Medicine* 24(145), 1-6.<br>**Study design**<br>Prospective cohort study<br>**Aim of the study**<br>The aim of the study was to investigate the association between pre-hospital administered RBCs and plasma and hemostatic function as evaluated by whole blood thrombelastography (TEG) on arrival at the trauma center as compared to that of patients not receiving pre-hospital blood transfusions.<br>**Setting**<br>United States, 2012-2013	**Inclusion criteria**<br>• adult trauma patients (≥16 years)<br>• met criteria for full trauma team activation<br>• received blood before arrival at ED or after hospital admittance within 6 h of ED arrival<br>**Exclusion criteria**<br>• pregnancy<br>• prisoner<br>• burned body surface area >20%<br>• enrolled in another study<br>**Characteristics**<br><u>Age [y], median (IQR)</u><br>IG: 34 (23 - 53)<br>CG: 39 (26 - 53), p=0.259<br><u>Sex male, n (%)</u><br>IG: 62 (82.7)<br>CG: 146 (80.2), p=0.650<br><u>ISS, median (IQR)</u><br>IG: 29 (17 - 41)<br>CG: 26 (17 - 34), p=0.106<br><u>GCS, median (IQR)</u><br>IG: 3 (3 - 15)<br>CG: 12 (3 - 15), p=0.022<br><u>Penetrating injury, n (%)</u>	**Participants**<br>N=257 patients<br>**Study groups**<br>IG: pre-hospital plasma and/or RBCs (PH) (N=75, 29%)<br>CG: in-hospital RBCs, plasma and platelets (IH) (N=182, 71%)	**Mortality**<br><u>6-h mortality, n (%)</u><br>IG: 10 (13.3) vs. CG: 15 (8.2), p=0.210<br><u>24-h mortality, n (%)</u><br>IG: 12 (16) vs. CG: 19 (10.4), p=0.213<br><u>In-hospital mortality, n (%)</u><br>IG: 20 (26.7) vs. CG: 38 (20.9), p=0.313<br>**Rapid thombelastography (rTEG) at hospital admission (median)**<br><u>MA</u><br>IG vs. CG: 62 vs. 64 (p=0.020)<br><u>G-value</u><br>IG vs. CG: 8.1 vs. 8.69 (p=0.009)<br><u>Multivariate linear regression with adjusting for pH, hemoglobin, platelet count, SBP, and in IG patients PH-RBC and PH-Plasma:</u><br>higher pH and higher platelet count were independent predictors of rTEG MA in CG patients and higher pH, higher platelet count and PH-Plasma were associated with higher rTEG MA in IG patients (all p<0.05, data not shown).	**Level of evidence**<br>3b↓<br>**Risk of bias**<br>Selection bias: –<br>Performance bias: ?<br>Attrition bias: ?<br>Detection bias: ?<br>**Authors' conclusion**<br>"Overall, these data imply that early administration of plasma may have beneficial effects on improving hemostasis, which may potentially translate into improved patient survival in future studies with larger sample sizes."<br>**Reviewers' conclusion**<br>There is a risk of selection bias as, for example, the IG had a higher incidence of penetrating injuries and lower GCS score than CG.

Study: Reference, aim, design, setting	Participants: selection criteria, characteristics	N Participants; Intervention (IG) vs. Control group (CG)	Main outcomes	Assessment: LoE, risk of bias; Conclusions
	IG: 34 (45.3)<br>CG: 46 (25.3), p=0.002<br><br>SBP [mmHg], median (IQR)<br>IG: 90 (77 - 113)<br>CG: 100 (80 - 125), p=0.044<br><br>Platelet count [x103/µL], median (IQR)<br>IG: 193 (152 - 223)<br>CG: 225 (174 - 257), p=0.001<br><br>pH, median (IQR)<br>IG: 7.21 (7.06 - 7.32)<br>CG: 7.27 (7.18 - 7.33), p=0.002<br><br>**Admission to 6 h total transfusions**<br><br>RBC [units], median (IQR)<br>IG: 8 (4 - 14)<br>CG: 3 (1 - 8), p<0.001<br><br>Plasma [units], median (IQR)<br>IG: 6 (3 - 12)<br>CG: 3(2 - 7), p<0.001<br><br>Platelets [packs], median (IQR)<br>IG: 6 (0 - 12)<br>CG: 0 (0 - 6), p<0.001			
**Holcomb (2017)**<br>"Multicenter observational prehospital resuscitation on helicopter study (PROHS)". *The Journal of Trauma and Acute Care Surgery* 83(1): S83-S91.	**Inclusion criteria**<br>• Estimated to be ≥15 years or ≥50 kg<br>• Traumatic injury<br>• Transported by helicopter directly from the scene of injury to one of nine Level I trauma centers<br>• Highest risk population including at least one of the following criteria	**Participants**<br>N=109 patients (1,058 prior to matching)<br><br>**Study groups**<br>IG: prehospital transfusion (plasma and/or red blood cells) (5 helicopters with blood available) (N=43; N=142 prior to matching)	Mortality 3h: n (%):<br>IG: 4 (9.3) vs. CG: 8 (12.1)<br>adjusted OR (95% CI) 0.74 (0.24–2.26) p=0.60<br><br>Mortality 24h:<br>IG: 5 (11.6) vs. 10 (15.2)<br>Adj OR (95% CI): 0.74 (0.25–2.17), p=0.58	**Level of evidence**<br>3b↓<br><br>**Risk of bias**<br>Selection bias: +<br>Performance bias: ?

Study: Reference, aim, design, setting	Participants: selection criteria, characteristics	N Participants; Intervention (IG) vs. Control group (CG)	Main outcomes	Assessment: LoE, risk of bias; Conclusions
**Study design**<br>Multicenter, prospective cohort study<br>**Aim of the study**<br>The hypothesis of this study was that patients with severe traumatic injuries evacuated to level 1 trauma centers on air ambulances who received prehospital red blood cells and/or plasma would have lower in-hospital mortality compared to patients transferred by air ambulance who received only crystalloid.<br>**Setting**<br>USA, 2015	• heart rate ≥120 bpm<br>• systolic blood pressure ≤90mmHg<br>• penetrating truncal injury<br>• tourniquet applied<br>• pelvic binder applied<br>• intubated prehospital<br>• received blood products during transport.<br>**Exclusion criteria**<br>• prisoners<br>• any transfers between hospitals<br>**Characteristics (after matching)**<br><u>Age, years, median (IQR)</u><br>IG: 48 (27-62)<br>CG: 39 (26-56)<br><u>Male, n (%)</u><br>IG: 29 (67.4)<br>CG: 48 (72.7)<br><u>ISS, median (IQR)</u><br>IG: 24 (10-34)<br>CG: 22 (10-34)<br><u>Systolic (mmHg) Blood Pressure, median (IQR)</u><br>IG: 110 (88-133)<br>CG: 105 (88-128)	CG: crystalloid resuscitation (4 helicopters without blood available) (N=66; N=473 prior to matching)<br>**Matching criteria**<br>Matching according to the following criteria (2:1 ratio of CG vs. IG):<br>• age<br>• gender<br>• race (white, black, other)<br>• injury severity score (ISS)<br>• prehospital vital signs (systolic blood pressure, diastolic blood pressure, pulse)<br>• whether or not patients satisfied more than one of the highest risk criteria (yes/no)<br>• presence of any penetrating injury (yes/no)<br>• use of a prehospital lifesaving intervention (yes/no)<br>• time from the air team call to arrival to the ED (in minutes)<br>• whether the bleeding source was identified prehospital (yes/no)<br>site volume (the total number of trauma patients arriving via helicopter)	<u>Mortality 30 days:</u><br>IG: 8 (18.6) vs. 14 (21.2)<br>Adj OR (95% CI): 0.85 (0.32–2.28), p=0.75	Attrition bias: –<br>Detection bias: +<br>**Authors' conclusion**<br>"In the primary analysis, prehospital blood product use was not significantly associated with 3 hour, 24 hour or 30 day mortality. However, the unexpected and significant differences in injury severity score, GCS and SBP resulted in lower power and therefore, the results are inconclusive."<br>**Reviewers' conclusion**<br>Power was low. More than 2/3 of cases were not available for analysis after matching because of systematic differences before matching. Therefore, the results need to be interpreted with caution.
**Holcomb (2015)**<br>"Transfusion of Plasma, Platelets, and Red Blood Cells in a 1:1:1 vs a 1:1:2 Ratio and Mortality in	**Inclusion criteria**<br>• Highest trauma level activation<br>• Estimated age of 15 years or older or weight of 50 kg or greater if age unknown	**Participants**<br>N=680 patients<br>**Study groups**	**Mortality (primary endpoint)**<br><u>24-h Mortality, n (%)</u><br>IG: 43 (12.7)<br>CG: 58 (17.0)	**Level of evidence**<br>1b<br>**Risk of bias**

Study: Reference, aim, design, setting	Participants: selection criteria, characteristics	N Participants; Intervention (IG) vs. Control group (CG)	Main outcomes	Assessment: LoE, risk of bias; Conclusions
Patients With Severe Trauma - The PROPPR Randomized Clinical Trial". *Journal of the American Medical Association* 313(5):471-482<br><br>**Study design**<br>Randomised controlled trial<br><br>**Aim of the study**<br>The aim of the study was to address the effectiveness and safety of a 1:1:1 transfusion ratio compared with a 1:1:2 transfusion ratio in patients with trauma who were predicted to receive a massive transfusion.<br><br>**Setting**<br>12 Level 1 trauma centers, North America, 2012-2013	• Received directly from the injury scene<br>• Initiated transfusion of at least 1 U of blood component within the first hour of arrival or during prehospital transport<br>• Predicted to receive a massive transfusion by exceeding the threshold score of either the Assessment of Blood Consumption score of 2 or greater or based on the attending trauma physician's judgment<br><br>**Exclusion criteria**<br>• Received lifesaving intervention from an outside hospital or health care facility<br>• Had devastating injuries and expected to die within 1 hour of admission<br>• Directly admitted from a correctional facility<br>• Required thoracotomy prior to receiving randomized blood products in the emergency department<br>• Younger than 15 years or weighed less than 50 kg if age unknown<br>• Known pregnancy<br>• burns covering greater than 20% total body surface area<br>• Suspected inhalation injury<br>• Received greater than 5 consecutive minutes of cardiopulmonary resuscitation (with chest compressions) prior to arriving at the hospital or within the emergency department<br>• Known do-not-resuscitate order prior to randomization	IG: Transfusion ratio 1:1:1 (N=338)<br><br>(Plasma, Platelets, Red Blood Cells (RBCs)<br>CG: Transfusion ratio 1:1:2 (N=342)<br><br>(Plasma, Platelets, Red Blood Cells (RBCs)	Difference, % (95% CI): IG vs. CG −4.2% (−9.6 - 1.1)<br><br>Adjusted RR (95% CI): IG vs. CG 0.75 (0.52 - 1.08)<br><br><u>30-d Mortality, n (%)</u><br>IG: 75 (22.4)<br>CG: 89 (26.1)<br><br>Difference (% (95% CI)): IG vs. CG −3.7 (−10.2 - 2.7)<br><br>Adjusted RR (95% CI): IG vs. CG 0.86 (0.65 - 1.12)<br><br>**Others**<br><u>Achieved hemostasis, n (%)</u><br>IG: 291 (86.1) vs. CG: 267 (78.1), p=0.006<br><br><u>Hospital-free days (median (IQR)</u><br>IG: 1 (0 – 17) vs. CG: 0 (0 – 16), p=0.83<br><br><u>Ventilator-free days (Median, IQR)</u><br>IG: 8 (0 – 16) vs. CG: 7 (0 – 14), p=0.14<br><br><u>ICU-free days (Median, IQR)</u><br>IG: 5 (0 – 11) vs. CG: 4 (0 – 10), p=0.10<br><br>**Cause of death, n (%)**<br><u>Exsanguination</u><br>24-h: IG: 31 (9.2) vs. CG: 50 (14.6), p=0.03<br>30-day: IG: 36 (10.7) vs. CG: 50 (14.7)<br><br><u>Traumatic Brain Injury</u><br>24-h: IG: 11 (3.3) vs. CG: 12 (3.5)<br>30-day: IG: 27 (8.1) vs. CG: 35 (10.3)	Selection bias: +<br><br>Performance bias: ?<br><br>Attrition bias: +<br><br>Detection bias: +<br><br>**Authors' conclusion**<br>"Among patients with severe trauma and major bleeding, early administration of plasma, platelets, and RBCs in a 1:1:1 ratio compared with a 1:1:2 ratio did not result in significant differences in mortality at 24 hours or 30 days. However, more patients in the 1:1:1 group achieved hemostasis and fewer experienced death due to exsanguination by 24 hours. Even though there was an increased use of plasma and platelets transfused in the 1:1:1 group, no other safety differences were identified between the 2 groups."<br><br>**Reviewers' conclusion**<br>There might be a risk of performance bias as could not be blinded and the care apart from the intervention was not standardized.

Study: Reference, aim, design, setting	Participants: selection criteria, characteristics	N Participants; Intervention (IG) vs. Control group (CG)	Main outcomes	Assessment: LoE, risk of bias; Conclusions
	• Enrolled in a concurrent, ongoing, interventional, randomized clinical trial<br>• Activated the opt-out process for the PROPPR trial (usually by wearing a bracelet given out at a community consent presentation)<br>• More than 3 U of red blood cells given before randomization<br><br>**Characteristics**<br><br><u>Age, years (median, IQR)</u><br>IG: 34.5 (25 – 51)<br>CG: 34 (24 – 50)<br><br><u>Sex (male, n (%))</u><br>IG: 263 (77.8)<br>CG: 283 (82.7)<br><br><u>GCS (median, IQR)</u><br>IG: 14 (3 – 15)<br>CG: 14 (3 – 15)<br><br><u>ISS (median, IQR)</u><br>IG: 26.5 (17 – 41)<br>CG: 26 (17 – 38)<br><br><u>Systolic blood pressure, mmHg (median, IQR)</u><br>IG: 102 (81 – 126)<br>CG: 102 (80 – 125)<br><br><u>INR (median, IQR)</u><br>IG: 1.3 (1.2 – 1.5)<br>CG: 1.3 (1.2 – 1.5)<br><br><u>Massive Transfusion (n (%))</u>		No differences in complications (such as acute respiratory distress syndrome, multiple organ failure, sepsis) at 30 days.	

Study: Reference, aim, design, setting	Participants: selection criteria, characteristics	N Participants; Intervention (IG) vs. Control group (CG)	Main outcomes	Assessment: LoE, risk of bias; Conclusions
	IG: 153 (45.3)<br>CG: 160 (46.8)			
**Moore (2018)**<br>"Plasma-first resuscitation to treat haemorrhagic shock during emergency ground transportation in an urban area: a randomised trial". *Lancet* 392(10144): 283-291.<br>**Study design**<br>Randomised controlled trial<br>(COMBAT trial)<br>**Aim of the study**<br>We tested the hypothesis that mortality would be lower among patients who received plasma before arrival at a level 1 trauma facility than among those who received standard care with normal saline.<br>**Setting**<br>USA, 2014-2017	**Inclusion criteria**<br>• injured adults (age >18 years)<br>• systolic blood pressure (SBP)≤70 mm Hg<br>• or SBP 71–90 mm Hg and heart rate 108 beats per min thought to be due to acute blood loss.<br>**Exclusion criteria**<br>• prisoner status<br>• known pregnancy<br>• isolated gunshot to the head<br>• asystole or cardiopulmonary resuscitation before randomization<br>• known objection to blood products<br>• opt-out bracelets or necklaces<br>• family objection to the patient's enrolment.<br>**Characteristics**<br><u>Age, years, median (IQR)</u><br>IG: 33.0 (25.0-51.0)<br>CG: 32.5 (25.5-42.0)<br><u>Male sex, n (%)</u><br>IG: 52 (80)<br>CG: 51 (85)<br><u>NISS, median (IQR)</u><br>IG: 27.0 (10.0–41.0)<br>CG: 27.0 (11.5–36.0)<br><u>INR (at scene of injury): median (IQR)</u>	**Participants**<br>Randomized N=144 patients<br>As treated N=125 patients<br>**Study groups**<br>IG: prehospital 2 U AB plasma (universal donor, ~250 mL each) in prepacked coolers (randomized N=75; as treated N=65)<br>CG: standard of care (normal saline 0.9%, volume based on haemodynamic need) (randomised N=69; as treated N=60)	**Primary endpoint**<br><u>Mortality (28 days), n (%)</u><br>As treated:<br>IG: 10 (15) vs. CG: 6 (10), p=0.37<br>Relative risk (95% CI): 1.54 (0.60 - 3.98)<br>ITT:<br>IG: 12 (16) vs. CG: 6 (9), p=0.19<br><u>Mortality (24h) (as treated): n (%)</u><br>IG: 8 (12) vs. CG: 6 (10), p=0.68<br>Relative risk (95% CI): 1.23 (0.45 – 3.34)<br><u>Multiple organ failure (28 days) (Denver score >3) (as treated): n (%)</u><br>IG: 4 (6) vs. CG: 1 (2), p=0.37<br>Relative risk (95% CI): 3.69 (0.42 – 32.11)<br>**Secondary endpoints (as treated)**<br><u>Composite outcome (multiple organ failure or death) at 28 days, n (%)</u><br>IG: 14 (21) vs. CG: 7 (12), p=0.14<br>Relative risk (95% CI): 1.85 (0.80 - 4.26)<br><u>Base deficit on arrival (mEq/L): median (IQR)</u><br>IG: 9.0 (5.5 – 13.0) vs. CG: 8.8 (6.0 – 13-0), p=0.8<br>Median difference (95% CI): 0 (−2.70 – 2.00)	**Level of evidence**<br>2b↓<br>**Risk of bias**<br>Selection bias: +<br>Performance bias: –<br>Attrition bias: +<br>Detection bias: +<br>**Authors' conclusion**<br>"Our findings indicate that plasma does not improve outcomes after injury when given within 30 min during rapid ground transportation to mature, level 1 trauma centres."<br>**Reviewers' conclusion**<br>The study has wide confidence intervals and was powered to detect a 19% difference in mortality. There may be a performance bias because masking of the care team was not possible and because patients in both groups did not receive similar volumes of plasma and placebo.

Study: Reference, aim, design, setting	Participants: selection criteria, characteristics	N Participants; Intervention (IG) vs. Control group (CG)	Main outcomes	Assessment: LoE, risk of bias; Conclusions
	IG: 1.1 (1.0-1.2)<br>CG: 1.1 (1.0-1.1)		Lactic acid concentration on arrival (mg/dL): median (IQR)<br>IG: 5.5 (3.9 – 8-5) vs. CG: 4.9 (3.2 – 7.0), p=0.3<br>Median difference (95% CI): 0.60 (−0.60 – 1.80)<br>INR on arrival at hospital, median (IQR)<br>IG: 1.27 (1.11 – 1.40) vs. CG: 1.15 (1.08 – 1.29), p=0.1<br>Median difference (95% CI): 0.60 (−0.01 – 0.14)	LoE downgraded due to post-hoc analysis.
**Pusateri (2020)**<br>"Association of Prehospital Plasma Transfusion With Survival in Trauma Patients With Hemorrhagic Shock When Transport Times Are Longer Than 20 Minutes: A Post Hoc Analysis of the PAMPer and COMBAT Clinical Trials". *JAMA Surgery* 155(2):e195085<br>**Study design**<br>Post-hoc subgroup analysis of data from 2 RCTs (PAMPer & COMBAT)<br>**Aim of the study**<br>Examine the combined data set to address the post hoc hypothesis that the benefits of prehospital administration of plasma	**For inclusion and exclusion criteria see PAMPer (Sperry 2018) & COMBAT trial (Moore 2018)**<br>**Characteristics**<br>Age, years, median (IQR)<br>Total: 42 (27-52)<br>IG: 43 (29-56)<br>CG: 42 (26-57)<br>Male sex, n (%)<br>Total: 467 (84.6)<br>IG: 216 (72.7)<br>CG: 251 (76.3)<br>ISS, median (IQR)<br>Total: 22 (12-34)<br>IG: 22 (12-34)<br>CG: 22 (12-33)	**Participants**<br>N=626 patients<br>(N=125 COMBAT, N=501 PAMPer)<br>**Study groups**<br>IG: prehospital plasma (N=297)<br>CG: standard care (crystalloid) (N=329)	**Influence of prehospital transport time**<br>(subgroup analysis of PAMPer & COMBAT)<br>Mortality (28 days, 1ary outcome): HR (95% CI)§<br>≤20 min transport time<br>IG vs. CG: 1.71 (0.70-4.16), p=0.24<br>>20 min transport time<br>IG vs. CG: 0.56 (0.40-0.80) p=0.001<br>Mortality (24 h): Hazard ratio (95% CI)§<br>≤20 min transport time<br>IG vs. CG: 1.89 (0.65-5.40), p=0.25<br>>20 min transport time<br>IG vs. CG: 0.53 (0.34-0.82) p=0.004<br>§Patients with event not reported per group, analyses adjusted for age and ISS	**Level of evidence**<br>2b↓<br>**Risk of bias**<br>Selection bias: +<br>Performance bias: –<br>Attrition bias: +<br>Detection bias: +<br>**Authors' conclusion**<br>"These data suggest that prehospital plasma is associated with a survival benefit when transport times are longer than 20 minutes and that the benefit-risk ratio is favorable for use of prehospital plasma."<br>**Reviewers' conclusion**<br>This is post-hoc subgroup analysis of harmonized data

Study: Reference, aim, design, setting	Participants: selection criteria, characteristics	N Participants; Intervention (IG) vs. Control group (CG)	Main outcomes	Assessment: LoE, risk of bias; Conclusions
are influenced by prehospital transport time.<br><br>**Setting**<br><br>USA, 2014-2019				from the PAMPer and COMBAT trial. There may be a performance bias because masking of the care team was not possible and because patients in both groups did not receive similar volumes of plasma and placebo.<br><br>LoE downgraded due to post-hoc analysis.
**Reitz (2020)**<br><br>"Prehospital plasma in injured patients is associated with survival principally in blunt injury: Results from two randomized prehospital plasma trials". *The Journal of Trauma and Acute Care Surgery*. 88(1):33-41<br><br>**Study design**<br><br>Post-hoc subgroup analysis of data from 2 randomised controlled trials (PAMPer & COMBAT)<br><br>**Aim of the study**<br><br>Our overall objective was to characterize prehospital plasma outcomes across mechanism of injury using	**For inclusion and exclusion criteria see PAMPer (Sperry 2018) & COMBAT trial (Moore 2018)**<br><br>**Characteristics**<br><br><u>Age, years, median (IQR)</u><br>Blunt trauma: 45 (28-61)<br><br>Penetrating trauma: 35 (26-49) (p<0.001)<br><br><u>Male sex, n (%)</u><br>Blunt trauma: 326 (70.1)<br><br>Penetrating trauma: 141 (87.6) (p<0.001)<br><br><u>ISS, median (IQR)</u><br>Total: 22 (12-34)<br><br>Blunt trauma: 24 (17-34)<br><br>Penetrating trauma: 14 (6-25) (p<0.001)<br><br><u>GCS: median (IQR)</u><br>Total: 6 (3-15)<br><br>Blunt trauma: 3 (3-15)	**Participants**<br><br>N=626 patients (N=501 PAMPer, N=125 COMBAT)<br><br>**Study groups**<br><br>IG: prehospital plasma (N=not reported)<br><br>CG: standard care (crystalloid) (N=not reported)<br><br>**Subgroup analysis mechanism of injury**<br><br><u>Blunt</u>: n=465, 75%, (including 10 suffering from blunt and penetrating trauma) (n=406 PAMPer, n=59 COMBAT)<br><br><u>Penetrating</u>: n=161, 25% (n=95 PAMPer, n=66 COMBAT)	**Mechanism of injury**<br>(subgroup analysis of PAMPer & COMBAT)<br><br><u>28-day mortality (primary endpoint): n (%)</u><br>*Blunt trauma*<br><br>IG: 50 (23.5) vs CG: 86 (34.1), p=0.012<br><br>Multivariate Cox-hazard regression HR (95% CI):<br><br>HR: 0.68 (0.472-0.965), p=0.031<br><br>*Penetrating trauma*<br><br>IG: 12 (14.3) vs. CG. 8 (10.4), p=0.454<br><br>Multivariate Cox-hazard regression HR (95% CI):<br><br>HR: 1.16 (0.430 – 3.103), p=0.775<br><br><u>24h mortality: n (%)</u><br>*Blunt trauma*<br><br>IG: 29 (15.2) vs. CG: 58 (25.8), p=0.010<br><br>Multivariate Cox-hazard regression HR (95% CI):<br><br>HR: 0.59 (0.370-0.947), p=0.029	**Level of evidence**<br><br>2b↓<br><br>**Risk of bias**<br><br>Selection bias: +<br><br>Performance bias: –<br><br>Attrition bias: +<br><br>Detection bias: +<br><br>**Authors' conclusion**<br><br>"A survival benefit associated with prehospital plasma at 24 hours and 28 days exists primarily in blunt injured patients with no benefit shown in penetrating trauma patients"<br><br>**Reviewers' conclusion**<br><br>This is post-hoc subgroup analysis of harmonized data

Study: Reference, aim, design, setting	Participants: selection criteria, characteristics	N Participants; Intervention (IG) vs. Control group (CG)	Main outcomes	Assessment: LoE, risk of bias; Conclusions
harmonized data obtained from these two recently completed prehospital plasma clinical trials. We hypothesized that the safety and beneficial effects of prehospital plasma would be consistent across blunt and penetrating mechanism of injury.<br>**Setting**<br>USA, 2014-2019	Penetrating trauma: 14 (3-15) (p=0.004)		*Penetrating trauma*<br>IG: 8 (10.4) vs. CG: 11 (13.23), p=0.595<br>Multivariate Cox-hazard regression HR (95% CI):<br>HR: 1.16 (0.430 – 3.103), p=0.775	from the PAMPer and COMBAT trial. There may be a performance bias because masking of the care team was not possible and because patients in both groups did not receive similar volumes of plasma and placebo.<br>LoE downgraded due to post-hoc analysis.
**Robinson (2018)**<br>“Risk Factors for the Development Of Acute Respiratory Distress Syndrome Following Hemorrhage“. *Shock* 50(3): 258-264.<br>**Study desgin**<br>secondary analysis of the PROPPR randomised controlled trial<br>**Aim of the study**<br>The aim of this secondary analysis was to clarify pulmonary outcomes in high-risk patients following severe injury with active hemorrhage after exposure to damage	**Inclusion criteria**<br>• Same as in PROPPR trial<br>**Exclusion criteria**<br>• Same as in PROPPR trial, additionally:<br>• patients with survival ≤24 hours<br>• lack of intensive care unit admission<br>• lack of recorded PaO2 to FiO2 (P/F) ratio during days 1-7<br>• naturally or synthetically derived colloids (e.g. albumin, hetastarch)<br>**Characteristics**<br>Age (median, IQR)<br>IG: 34.5 (25 - 49) (p=0.65)<br>CG: 33 (24 - 50)<br>Sex (male), n (%)<br>IG: 179 (77.8) (p=0.37)<br>CG: 182 (81.3)	**Participants**<br>N=454 patients, subset of the original 680 patients<br>**Study groups**<br>IG: Transfusion ratio 1:1:1 (N=230)<br>(Plasma, Platelets, Red Blood Cells (RBCs)<br>CG: Transfusion ratio 1:1:2 (N=224)<br>(Plasma, Platelets, Red Blood Cells (RBCs)	**Pulmonary outcomes**<br>ARDS, n (%)<br>IG: 34 (14.8), p=0.35<br>CG: 41 (18.3)<br>Hospital day ARDS occurred, during hospital day 1-7, median (IQR)<br>IG: 3.5 (1 - 6), p=0.06<br>CG: 2 (1 - 4)<br>Ventilator days, median (IQR)<br>IG: 5 (2 - 13), p=0.98<br>CG: 4.5 (2 - 14)<br>Ventilator-free days in 30, median (IQR<br>IG: 24 (9 - 27), p=0.90<br>CG: 24 (9.5 - 28)<br>**Mortality**<br>7-day mortality, n (%)	**Level of evidence**<br>2b↓<br>**Risk of bias**<br>Same as in PROPPR trial<br>**Authors’ conclusion**<br>“Acute crystalloid exposure, but not blood products, emerges as a modifiable risk factor for the prevention of ARDS following hemorrhage. Relatively small volumes of crystalloid fluids given during the acute period of resuscitation appear to be associated with the development of lung injury.”<br>**Reviewers’ conclusion**

Study: Reference, aim, design, setting	Participants: selection criteria, characteristics	N Participants; Intervention (IG) vs. Control group (CG)	Main outcomes	Assessment: LoE, risk of bias; Conclusions
control resuscitation practices<br><br>**Setting**<br><br>North America, same as PROPPR trial	<u>ISS (median, IQR)</u><br><br>IG: 29 (19 - 41) (p=0.31)<br>CG: 28 (18 - 38)<br><br><u>ED GCS (median, IQR)</u><br><br>IG: 14 (3 - 15) (p=0.32)<br>CG: 14 (3 - 15)<br><br><u>ED SBP<90 (mmHg, n (%))</u><br><br>IG: 76 (33.3) (p=0.53)<br>CG: 66 (30.6)<br><br><u>Massive Transfusion (n (%))</u><br><br>IG: 108 (47.0) (p=0.52)<br>CG: 112 (50.0)		IG: 18 (7.8), p=0.60<br>CG: 14 (6.3)<br><br><u>30-day mortality, n (%)</u><br><br>IG: 28 (12.3), p=0.91<br>CG: 28 (12.5)<br><br><u>Risk factors for ARDS (entire study group, multivariate analysis), OR (95% CI)</u><br><br>Blunt mechanism: 3.61 (1.53 - 8.51), p<0.01<br>Chest AIS: 1.40 (1.15 - 1.71), p<0.01<br>IVFs given (by 500 mL units) in hours 0-6: 1.09 (1.04 - 1.14), p<0.01	Post-hoc analysis of RCT data. The intervention and control groups were balanced with respect to baseline characteristics.
**Sperry (2018)**<br><br>"Prehospital Plasma during Air Medical Transport in Trauma Patients at Risk for Hemorrhagic Shock ", *The New England Journal of Medicine* 379(4),315-26.<br><br>**Study design**<br><br>cluster-randomised clinical trial (PAMPer trial)<br><br>**Aim of the study**<br><br>The aim of the study was to determine the efficacy and safety of prehospital plasma resuscitation as compared with standard-care resuscitation in	**Inclusion criteria**<br><br>• Patients transported from scene of injury to a participating trauma center or who were transferred from an outside referral emergency department to a participating trauma center<br>• at least one episode of hypotension (systolic blood pressure <90 mm Hg) and tachycardia (heart rate >108 bpm)<br>• OR any severe hypotension (systolic blood pressure <70 mm Hg), either before the arrival of air medical transport or any time before arrival at the trauma center<br><br>**Exclusion criteria**<br><br>• Aged >90 years or <18 years<br>• intravenous or intraosseous access could not be established	**Participants**<br><br>N=501 patients<br><br>**Study groups**<br><br>IG: prehospital thawed plasma (N=230)<br><br>2 units of either group AB or group A with a low anti-B antibody titer (<1:100) thawed plasma infused to completion<br><br>CG: Standard-care resuscitation (N=271)<br><br>infusion of a crystalloid solution as the primary resuscitative fluid<br><br>Note: 13 of 27 air medical bases also carried 2 units of infusion of **universal donor red cells** which were allowed to be administered to IG (after plasma) or placebo	**Mortality**<br><br><u>Primary outcome: 30-day mortality§: n (%)</u><br><br>IG: 53 (23.2),<br>CG: 89 (33.0)<br><br>Difference in % (95% CI): −9.8% (−18.6 to −1.0); p=0.03<br><br><u>Risk of death within 30 days, OR (95% CI)</u><br><br>IG vs. CG (adj.): 0.61 (0.40 - 0.91), p=0.02<br><br><u>24-hour mortality, n (%), % difference (95% CI)*</u><br><br>IG: 32 (13.9), −8.2% (−14.9 to −1.6), adj. p=0.55<br>CG: 60 (22.1)<br><br><u>In-hospital mortality, n (%), % difference (95% CI)*</u><br><br>IG: 51 (22.2), −10.3% (−18.0 to −2.6), p=0.33<br>CG: 88 (32.5)<br><br>**Others**	**Level of evidence**<br><br>1b<br><br>**Risk of bias**<br><br>Selection bias: +<br><br>Performance bias: –<br><br>Attrition bias: +<br><br>Detection bias: +<br><br>**Authors' conclusion**<br><br>"In injured patients at risk for hemorrhagic shock, the administration of thawed plasma during prehospital air medical transport was safe and resulted in lower 30-day mortality and a lower median prothrombin-

Study: Reference, aim, design, setting	Participants: selection criteria, characteristics	N Participants; Intervention (IG) vs. Control group (CG)	Main outcomes	Assessment: LoE, risk of bias; Conclusions
severely injured patients at risk for hemorrhagic shock.<br><br>**Setting**<br><br>United States, 2014-2017	• isolated fall from standing<br>• a documented cervical cord injury<br>• known to be a prisoner<br>• known pregnancy<br>• traumatic cardiac arrest lasting >5 minutes<br>• penetrating brain injury<br>• injury due to isolated drowning or hanging<br>• burns >20% of total body- surface area<br>• admitted as an inpatient at an outside referral hospital<br>• if patient or family member voiced an objection to participation in the trial at the scene of the injury<br>• wearing an "opt-out" bracelet, indicating to opt out of the PAMPer trial<br><br>**Characteristics**<br><br>Age (median, IQR)<br>IG: 44 (31–59)<br>CG: 46 (28–60)<br><br>Male sex, n (%)<br>IG: 164 (71.3)<br>CG: 200 (73.8)<br><br>GCS <8, n (%)<br>IG: 103 (44.8)<br>CG: 129 (47.6)<br><br>ISS (median, IQR)<br>IG: 22 (14–33)<br>CG: 21 (12–29)		Multiorgan failure, n (%)<br>IG: 145 (63.0), 5.4% (−3.1 to 14.1), p>0.99*<br>CG: 156 (57.6)<br><br>Acute lung injury–acute respiratory distress syndrome, n (%)*<br>IG: 48 (20.9), 2.4% (−4.8 to 9.4), p>0.99<br>CG: 50 (18.5)<br><br>initial prothrombin-time ratio; median (IQR)*<br>IG: 1.2 (1.1 - 1.4), p<0.001<br>CG: 1.3 (1.1 - 1.6)<br><br>*Significance levels were adjusted with the use of a Bonferroni correction to account for multiple comparisons.<br><br>§after multiple imputation	time ratio than standard-care resuscitation."<br><br>**Reviewers' conclusion**<br><br>There may be a risk of performance bias since the staff was not masked to the intervention because the trial intervention was a blood product, which requires full traceability.

Study: Reference, aim, design, setting	Participants: selection criteria, characteristics	N Participants; Intervention (IG) vs. Control group (CG)	Main outcomes	Assessment: LoE, risk of bias; Conclusions
	prehospital systolic blood pressure [mmHg], median (IQR)<br>IG: 71 (64–81)<br>CG: 69 (61–81)			

Tranexamic acid

Study: Reference, aim, design, setting	Participants: selection criteria, characteristics	N Participants; Intervention (IG) vs. Control group (CG)	Main outcomes	Assessment: LoE, risk of bias; Conclusions
**Bossers (2021)**<br>"Association between prehospital tranexamic acid administration and outcomes of severe traumatic brain injury." *JAMA Neurology* 78.3: 338-345.<br>**Study design**<br>Comparative registry study<br>(BRAIN-PROTECT)<br>**Aim of the study**<br>"In this cohort study, we aimed to assess whether prehospital administration of tranexamic acid is associated with mortality and functional outcomes in a group of patients with severe TBI."<br>**Setting**	**Inclusion criteria**<br>• patients with suspected severe traumatic brain injury (TBI) (based on a trauma mechanism or clinical findings suggestive of severe TBI and a prehospital GlasgowComa Scale [GCS] score of 8 or lower)<br>• treated by 1 of the 4 Dutch physician-staffed Helicopter Emergency Medical Services (HEMS)<br>**Exclusion criteria**<br>• not transported to a participating trauma center (no follow-up data were available)<br>• patients who were undergoing prehospital traumatic cardiopulmonary resuscitation (inherently very high mortality, regardless of treatment)<br>**Characteristics**<br>Age, years, median (IQR)<br>IG: 47 (25-66) vs. CG: 45 (22-65), p=0.03<br>(missing data: N=20 / 1827)	**Participants**<br>N=1827 patients<br>**Study groups**<br>IG: Patients who received tranexamic acid (N=693)*<br>CG: Patient who did not receive tranexamic acid (N=1134)<br><br>*in 680 patients, tranexamic dose was documented, 90% received a dose of 1g	**Primary outcomes**<br>30-day mortality n (%)<br>IG: 241 (37) vs. CG: 322 (30), p=0.005<br>(missing data: N=113/1827)<br>Confounder-adjusted logistic regression, original data set, OR (95% CI)<br>Full cohort: 1.18 (0.73-1.90), p=0.51<br>Confirmed TBI cohort: 1.27 (0.68-2.35) p=0.45<br>Isolated TBI cohort: 4.49 (1.57-12.87), p=0.005<br>Confounder-adjusted logistic regression, after multiple imputations, OR (95% CI)<br>Full cohort: 1.17 (0.84-1.65), p=0.35<br>Confirmed TBI cohort: 1.19 (0.92-1.53), p=0.19<br>Isolated TBI cohort: 2.05 (1.22-3.45), p=007<br>Sensitivity analyses<br>- Confounder-adjusted survival analysis, original data set, HR (95% CI):<br>Full cohort: 1.10 (0.92-1.31), p=0.30	**Level of evidence**<br>2b<br>**Risk of bias**<br>Selection bias: ?<br>Performance bias: ?<br>Attrition bias: +<br>Detection bias: +<br>**Authors' conclusion**<br>"This study found that prehospital tranexamic acid administration was associated with increased mortality in patients with isolated severe TBI, suggesting the judicious use of the drug when no evidence for extracranial hemorrhage is present."<br>**Reviewers' conclusion**<br>There might be a risk of selection bias as the groups

Study: Reference, aim, design, setting	Participants: selection criteria, characteristics	N Participants; Intervention (IG) vs. Control group (CG)	Main outcomes	Assessment: LoE, risk of bias; Conclusions
Netherlands, 2012-2018	<u>Male sex, n (%)</u><br>IG: 486 (70) vs. CG: 797 (70), p=0.94<br>(missing data: N=3 / 1827)<br><u>ISS, median (IQR)</u><br>IG: 27 (21-38) vs. CG: 26 (17-34), p<0.001<br>(missing data: N=208 / 1827)<br><u>GCS score, median (IQR)</u><br>IG: 4 (3-6) vs. CG: 5 (3-7), p<0.001<br><u>Confirmed TBI (Head AIS ≥3)</u><br>Overall: n=1375<br><u>Isolated TBI (Head AIS ≥3, with neck, spine, thorax, abdomen, extremities, and external AIS ≤2)</u><br>Overall: n=719		Confirmed TBI cohort: 1.10 (0.90-1.35), p=0.35<br>Isolated TBI cohort: 1.66 (1.08-2.54), p=0.02<br><u>- Confounder-adjusted survival analysis, after multiple imputations, HR (95% CI):</u><br>Full cohort: 1.01 (0.90-1.14), p=0.81<br>Confirmed TBI cohort: 1.00 (0.91-1.11), p=0.97<br>Isolated TBI cohort: 1.34 (1.16-1.55), p<0.001<br>**Secondary outcomes**<br><u>Hospital Length of Stay, median (IQR)</u><br>IG: 17 (7.5-35) vs. CG: 15 (5-31) p=0.007<br>(missing data: N=11 / 1827)<br><u>Adjusted analyses, original data set, incidence rate ratio (IRR) (95% CI)</u><br>Full cohort: 0.92 (0.81-1.04), p=0.17<br>Confirmed TBI cohort: 0.89 (0.77-1.03), p=0.11<br>Isolated TBI cohort: 0.71 (0.54-0.95), p=0.02<br><u>Adjusted analyses, after multiple imputations, IRR (95% CI)</u><br>Full cohort: 1.05 (0.92-1.20), p=0.44<br>Confirmed TBI cohort: 1.04 (0.88-1.22), p=0.65<br>Isolated TBI cohort: 0.88 (0.69-1.11), p=0.27<br><u>Glasgow Outcome Scale (GOS) score at discharge, n (%), p=0.001</u><br>(missing data: N=170 / 1827)<br>- Death, IG: 250 (39) vs. CG: 323 (32)<br>- Vegetative state, IG: 19 (3) vs. CG: 19 (2)	differ in baseline factors, such as age and injury severity, and due to missing data. However, several adjustments for confounders and sensitivity analyses were done, showing consistent results.

Study: Reference, aim, design, setting	Participants: selection criteria, characteristics	N Participants; Intervention (IG) vs. Control group (CG)	Main outcomes	Assessment: LoE, risk of bias; Conclusions
			- Severe disability, IG: 214 (34) vs. CG: 385 (38)<br>- Moderate disability, IG: 70 (11) vs. CG: 107 (10)<br>- Good recovery, IG: 80 (13) vs. CG: 190 (19)<br><u>Adjusted analyses, original data set, OR (95% CI)</u><br>Full cohort: 1.05 (0.81-1.37), p=0.71<br>Confirmed TBI cohort: 0.97 (0.72-1.30), p=0.83<br>Isolated TBI cohort: 0.68 (0.25-1.90), p=0.47<br><u>Adjusted analyses, after multiple imputations, OR (95% CI)</u><br>Full cohort: 0.97 (0.78-1.21), p=0.79<br>Confirmed TBI cohort: 0.86 (0.72-1.04), p=0.13<br>Isolated TBI cohort: 0.69 (0.42-1.14) 0 .15<br>**12-month Mortality**<br><u>Adjusted analyses, original data set, OR (95% CI)</u><br>Full cohort: 1.08 (0.66-1.76), p=0.76<br>Confirmed TBI cohort: 1.04 (0.60-1.79), p=0.89<br>Isolated TBI cohort: 3.31 (1.20-9.16), p=0.02<br><u>Adjusted analyses, after multiple imputations, OR (95% CI)</u><br>Full cohort: 0.99 (0.77-1.29), p=0.96<br>Confirmed TBI cohort: 0.99 (0.75-1.30), p=0.93<br>Isolated TBI cohort: 1.78 (1.13-2.80), p=0.01	
**Brenner (2020)**<br>"Understanding the neuroprotective effect of tranexamic acid: an	**Inclusion criteria**<br>• Adults with traumatic brain injury (TBI) ≤3 h of injury	**Participants**<br>N=7637 patients (after excluding patients with GCS score of 3 or bilateral unreactive	**Effect of tranexamic acid on early deaths (within 24 h)**<br><u>All patients, n (%), RR (95% CI)</u>	**Level of evidence**<br>2b↓<br>**Risk of bias**

Study: Reference, aim, design, setting	Participants: selection criteria, characteristics	N Participants; Intervention (IG) vs. Control group (CG)	Main outcomes	Assessment: LoE, risk of bias; Conclusions
exploratory analysis of the CRASH-3 randomised trial." *Critical Care* 24.1: 1-10.<br><br>**Study design**<br><br>Exploratory analysis of a randomised controlled trial (CRASH-3)<br><br>**Aim of the study**<br><br>To explore the mechanism of action of TXA in TBI, we examined the timing of its effect on death.<br><br>**Setting**<br><br>29 countries, 2012-2019	• GCS score ≤12 or intracranial bleeding on CT<br>• no significant extra-cranial bleeding<br><br>**Exclusion criteria**<br><br>• GCS score of 3<br>• Bilateral unreactive pupils<br><br>**Characteristics**<br><br>Patient characteristics for all patients with TBI within 3 h of injury: see CRASH-3	pupils); exclusion of 98 patients with missing outcome data<br><br>**Study groups**<br><br>IG: TXA<br><br>1 g of tranexamic acid infused over 10 min, started immediately after randomisation, followed by an intravenous infusion of 1 g over 8 h (four ampules of TXA 500 mg)<br><br>CG: 100 mL bag of 0.9% sodium chloride<br><br>(Number of patients per group not reported)	IG: 112 (2.9) vs. CG: 147 (3.9), 0.74 (0.58-0.94)<br><br><u>Severity n (%), RR (95% CI)</u><br>*Mild/moderate (GCS 9-15):*<br><br>IG: 25 (0.9) vs. CG: 37 (1.3), 0.66 (0.40-1.09)<br><br>*Severe (GCS 3-8):*<br><br>IG: 87 (8.5) vs. CG: 110 (11.3), 0.75 (0.58-0.98)<br><br><u>Country income, n (%), RR (95% CI)</u><br>*LMIC:*<br><br>IG: 98 (3.3) vs. CG: 126 (4.4), 0.75 (0.58–0.98)<br><br>*HIC:*<br><br>IG: 14 (1.5) vs. CG: 21 (2.4), 0.65 (0.33–1.26)<br><br>**Effect of tranexamic acid on deaths after 24 h**<br><br><u>All patients, n (%), RR (95% CI)</u><br>IG: 432 (11.5) vs. CG: 421 (11.7), 0.98 (0.69–1.12)<br><br><u>Severity n (%), RR (95% CI)</u><br>*Mild/moderate (GCS 9-15):*<br><br>IG: 163 (5.8) vs. CG: 186 (6.9), 0.85 (0.69–1.04)<br><br>*Severe (GCS 3-8):*<br><br>IG: 269 (28.7) vs. CG: 235 (27.2), 1.05 (0.91–1.22)<br><br><u>Country income, n (%), RR (95% CI)</u><br>*LMIC:*<br><br>IG: 363 (12.6) vs. CG: 344 (12.5), 1.01 (0.88–1.16)<br><br>*HIC:*<br><br>IG: 69 (7.7) vs. CG: 77 (9.0), 0.86 (0633–1.18)	See CRASH-3 (below)<br><br>**Authors' conclusion**<br><br>"Tranexamic acid reduces early deaths in non-moribund TBI patients regardless of TBI severity or country income. The effect of tranexamic acid in patients with isolated TBI is similar to that in polytrauma. Treatment is safe and <u>even severely injured patients appear to benefit when treated soon after injury</u>."<br><br>**Reviewers' conclusion**<br><br>The exploratory analysis of the CRASH-3 trial is of good quality indicating reliable results.<br><br>Downgraded due to post-hoc analysis.

Study: Reference, aim, design, setting	Participants: selection criteria, characteristics	N Participants; Intervention (IG) vs. Control group (CG)	Main outcomes	Assessment: LoE, risk of bias; Conclusions
			**Effect of tranexamic acid on deaths at 28 days**<br><br><u>All patients, n (%), RR (95% CI)</u><br>IG: 544 (14.0) v. CG: 568 (15.1), 0.93 (0.83–1.03)<br><br><u>Severity n (%), RR (95% CI)</u><br>*Mild/ moderate (GCS 9-15):*<br>IG: 188 (6.7) vs. CG: 223 (8.1), 0.82 (0.68–0.99)<br>*Severe (GCS 3-8):*<br>IG: 356 (34.7) vs. CG: 345 (35.4), 0.98 (0.87–1.10)<br><br><u>Country income, n (%), RR (95% CI)</u><br>*LMIC:*<br>IG: 461 (15.5) vs. CG: 470 (16.3), 0.95 (0.84–1.07)<br>*HIC:*<br>IG: 83 (9.2) vs. CG: 98 (11.1), 0.82 (0.62–1.08)<br><br>**Effects of tranexamic acid on vascular occlusive events (fatal and non-fatal) in all patients irrespective of time to treatment**<br><br><u>Severe (GCS 3-8), n/N (%), RR (95% CI):</u><br>IG: 60/2264 (2.7) vs. CG: 50/2247 (2.2), 1.19 (0.82–1.73)<br><br>Pooled data from the CRASH-2 and CRASH-3 trials were not reported separately for severe TBI.	
**CRASH-3 collaborators (2019).**<br>"Effects of tranexamic acid on death, disability, vascular occlusive events	**Inclusion criteria**<br>• Adults with traumatic brain injury (TBI) ≤3 h of injury (at the beginning ≤8 h)<br>• Glasgow Coma Scale (GCS) score ≤12	**Participants**<br>N=12,737 patients, out of whom 9,202 (72.2%) patients within 3 h of injury.<br><br>**Study groups**	All results for patients randomly assigned within 3h:<br><br>**Head injury-related death in hospital (28 days): n/N (%)**<br><br><u>overall:</u>	**Level of evidence**<br>1b<br><br>**Risk of bias**<br>Selection bias: +

Study: Reference, aim, design, setting	Participants: selection criteria, characteristics	N Participants; Intervention (IG) vs. Control group (CG)	Main outcomes	Assessment: LoE, risk of bias; Conclusions
and other morbidities in patients with acute traumatic brain injury (CRASH-3): a randomised, placebo-controlled trial". *Lancet* 394(10210): 1713-23<br><br>**Study design**<br><br>Randomised controlled trial<br><br>**Aim of the study**<br><br>The CRASH-3 trial aimed to quantify the effects of tranexamic acid on head injury-related death, disability, and adverse events in patients with TBI.<br><br>**Setting**<br><br>29 countries, 2012-2019	• OR any intracranial bleeding on CT scan,<br>• no major extracranial bleeding<br>• responsible clinician was substantially uncertain as to the appropriateness of tranexamic acid treatment<br><br>**Baseline characteristics of those randomly assigned within 3 h of injury**<br><br><u>Age, years, mean ± SD</u><br>IG: 41.7 (19.0)<br>CG: 41.9 (19.0)<br><br><u>Male sex, n (%)</u><br>IG: 3,742 (80)<br>CG: 3,660 (80)<br><br><u>Glagow Coma Scale Scores, n (%)</u><br><u>GCS 3</u>: IG: 495 (11) vs. CG: 506 (11)<br><u>GCS 4</u>: IG: 213 (5) vs. CG: 213 (5)<br><u>GCS 5</u>: IG: 163 (4) vs. CG: 172 (4)<br><u>GSC 6</u>: IG: 221 (5) vs. CG: 232 (5)<br><u>GCS 7:</u> IG: 311 (7) vs. CG: 294 (6)<br><u>GCS 8</u>: IG: 354 (8) vs. CG: 315 (7)<br><u>GCS 9</u>: IG: 335 (7) vs. CG: 292 (6)<br><u>GCS 10</u>: IG: 371 (8) vs. CG: 364 (8)<br><u>GCS 11</u>: IG: 375 (8) vs. CG: 390 (9)<br><u>GCS 12</u>: IG: 476 (10) vs. CG: 478 (10)<br><u>GCS 13</u>: IG: 297 (6) vs. CG: 312 (7)<br><u>GCS 14</u>: IG: 526 (11) vs. CG: 458 (10)	IG: TXA (N=6,406; 4,649 ≤3 h)<br><br>1 g of tranexamic acid infused over 10 min, started immediately after randomisation, followed by an intravenous infusion of 1 g over 8 h (four ampules of TXA 500 mg)<br><br>CG: 100 mL bag of 0.9% sodium chloride (N=6,331; 4,553 ≤3 h)	IG: 855/4,613 (18.5) vs. CG: 892/4514 (19.8)<br>Risk ratio (95% CI): 0.94 (0.86–1.02)<br><br><u>GCS severe (3-8)</u><br>IG: 689/1,739 (39.6) vs. GC: 685/1,710 (40.1)<br>Risk ratio (95% CI): 0.99 (0.91–1.07)<br><br>no obvious effect of time to treatment in patients with severe head injury (p=0·73).<br><br>**Stratification by time**<br><br>Early treatment was more effective than later treatment in patients with mild and moderate head injury (p=0·005) but we found no obvious effect of time to treatment in patients <u>with severe head injury</u> (p=0·73).<br><br>Other endpoints were not reported separately for severe TBI.	Performance bias: +<br><br>Attrition bias: +<br><br>Detection bias: +<br><br>**Authors' conclusion**<br><br>"We found a substantial reduction in head injury-related deaths with tranexamic acid in patients with mild and moderate head injuries <u>but no apparent reduction in those with severe head injury</u>."<br><br>**Reviewers' conclusion**<br><br>CRASH-3 is a randomised controlled trial of good quality with large sample size, indicating reliable results.

Study: Reference, aim, design, setting	Participants: selection criteria, characteristics	N Participants; Intervention (IG) vs. Control group (CG)	Main outcomes	Assessment: LoE, risk of bias; Conclusions
	GCS 15: IG: 484 (10) vs. CG: 492 (11)<br>Unknown: IG: 28 (1) vs. CG: 35 (1)			
**Guyette (2020)**<br>"Tranexamic Acid During Prehospital Transport in Patients at Risk for Hemorrhage After Injury: A Double-blind, Placebo-Controlled, Randomized Clinical Trial". *JAMA Surgery* (2021); 156(1): 11-20<br>**Study design**<br>Randomised controlled trial<br>(STAAMP)<br>**Aim of the study**<br>To assess the effectiveness and safety of tranexamic acid administered before<br>hospitalization compared with placebo in injured patients at risk for hemorrhage.<br>**Setting**<br>USA, 2015-2019	**Inclusion criteria**<br>• injured patients at risk for hemorrhage transported from the scene or transferred from an outside emergency department<br>• at least 1 episode of hypotension (systolic blood pressure ≤90 mmHg) or tachycardia (heart rate ≥110 beats per minute) before arrival at a participating center<br>**Exclusion criteria**<br>• age >90 years, <18 years<br>• lack of intravenous or intraosseous access<br>• isolated fall from standing<br>• documented cervical cord injury<br>• known prisoner or pregnancy<br>• traumatic arrest of >5 minutes<br>• penetrating brain injury<br>• isolated drowning or hanging<br>• objection to study voiced at scene<br>• wearing a STAAMP study opt-out bracelet.<br>**Characteristics**<br>Age, years, mean ± SD<br>Overall: 42 ± 18<br>IG: 41 ± 17<br>CG: 42 ± 18<br>Male sex, n (%)	**Participants**<br>N=903 patients<br>**Study groups**<br>IG: TXA (N=477)<br>The treatment arms received a 1-g bolus of tranexamic acid (for 10 minutes) en route to the hospital.<br>• TXA abbreviated: 1g TXA + placebo + placebo (N=151)<br>• TXA standard: 1g TXA +1g TXA + placebo (N=141)<br>• TXA repeat: 1g TXA + 1g TXA + 1g TXA (N=150)<br>CG: placebo bolus + placebo bolus + placebo infusion (N=456)<br>**Co-interventions**<br>Phase A (prehospital): infusion over 10 min<br>• 1 g of TXA 10mL of solution + 100-mL bag of 0.9% saline<br>• 10 mL of sterile water + 100-mL bag of 0.9% saline.<br>Phase B intervention (hospital): infusion over 10 min<br>• 1g TXA in 10 mL of solution	**Mortality (30 days): n/N (%)**<br>Overall<br>IG: 36/442 (8.1) vs. 45/452 (10.0) (9 missing)<br>Risk ratio (95% CI) 0.82 (0.60-1.11)<br>By dosing regimen<br>*TXA abbreviated* 14/150 (9.3) vs. CG: 45/452 (10.0)<br>Risk ratio (95% CI): 0.94 (0.65-1.36), p=0.74<br>*TXA standard* 11/141 (7.8) vs. CG: 45/452 (10.0)<br>Risk ratio (95% CI): 0.78 (0.50-1.24), p=0.30<br>*TXA repeat* 11/151 (7.3) vs. CG: 45/452 (10.0)<br>Risk ratio (95% CI): 0.73 (0.54-0.99), p=0.04<br>**By TBI severity**<br>No severe TBI (head AIS≤2)<br>IG: 17/352 (4.8) vs. CG: 25/374 (6.7)<br>Risk ratio (95% CI): 0.72 (0.46-1.14)<br>Severe TBI (head AIS>2)<br>IG: 19/90 (21.1) vs. 20/78 (25.6)<br>Risk ratio (95% CI): 0.82 (0.55-1.24)<br>Adjusted p=0.86 for interaction<br>**By transfusion received**<br>No transfusion received	**Level of evidence**<br>1b<br>**Risk of bias**<br>Selection bias: +<br>Performance bias: +<br>Attrition bias: +<br>Detection bias: +<br>**Authors' conclusion**<br>"Patients with severe shock (systolic blood pressure ≤70mmHg) who received tranexamic acid demonstrated lower 30-day mortality compared with placebo."<br>**Reviewers' conclusion**<br>The study has a low risk of bias. The subgroup of severe shock patients (SBP ≤70mmHg) contains a small number of patients (N=58).

Study: Reference, aim, design, setting	Participants: selection criteria, characteristics	N Participants; Intervention (IG) vs. Control group (CG)	Main outcomes	Assessment: LoE, risk of bias; Conclusions
	Overall: 686 (74.0)<br>IG: 327 (73.2)<br>CG: 341 (74.8)<br>ISS, median (IQR)<br>Overall: 12 (5-22)<br>IG: 13 (5-22)<br>CG: 11 (4-22)<br>Initial GCS<8: n (%)<br>IG: 89 (19.9)<br>CG: 107 (23.5)	• 10 mL of placebo (sterile water) added to a 100 mL bag of 0.9% saline<br>Phase C intervention (hospital): infusion over 8h<br>• 1 g of TXA in 10 mL of solution<br>10 mL of placebo + 100-mL bag of 0.9% saline	IG: 10/289 (3.5) vs. CG: 10/295 (3.4)<br>Risk ratio (95% CI): 1.02 (0.49-2.15)<br>Transfusion received<br>IG: 26/153 (17.0) vs. 35/157 (22.3)<br>Risk ratio (95% CI): 0.76 (0.57-1.01)<br>Adjusted p=0.32 for interaction<br>**By shock severity** (post-hoc analysis)<br>Tachycardia only<br>IG: 18/316 (5.7) vs. CG: 21/320 (6.6)<br>Risk ratio (95% CI): 0.87 (0.56-1.34), p=0.52<br>SBP <90 mm Hg<br>IG: 13/99 (13.1) vs. CG: 13/101 (12.9)<br>Risk ratio (95% CI): 1.02 (0.55-1.90), p=0.95<br>SBP <70 mm Hg<br>IG: 5/27 (18.5) vs. CG: 11/31 (35.5)<br>Risk ratio (95% CI): 0.52 (0.34-0.80), p=0.003<br>**By time from injury** (post-hoc analysis)<br>≤1h<br>IG: 10/219 (4.6) vs. CG: 18/238 (7.6)<br>Risk ratio (95% CI): 0.60 (0.44-0.83)<br>>1h<br>IG: 26/223 (11.7) vs. 27/214 (12.6)<br>Risk ratio (95% CI): 0.92 (0.52-1.64)<br>§only percentage reported	
**Khan (2018)**	In Addition to PROPPR<br>**Inclusion criteria**	**Participants**	**Mortality§** - *primary outcomes*	**Level of evidence**

Study: Reference, aim, design, setting	Participants: selection criteria, characteristics	N Participants; Intervention (IG) vs. Control group (CG)	Main outcomes	Assessment: LoE, risk of bias; Conclusions
„Severely Injured Trauma Patients With Admission Hyperfibrinolysis; Is There A Role Of Tranexemic Acid? Findings From The PROPPR Trial", *Journal of Trauma and Acute Care Surgery, 85(5): 851–857*<br><br>**Study design**<br>Comparative registry trial (secondary analysis of PROPPR database)<br><br>**Aim of the study**<br>The aim of the study was to analyze the role of TXA in severely injured trauma patients with admission hyperfibrinolysis.<br><br>**Setting**<br>North America, PROPPR Trial 2012-2013	• trauma patients with hyperfibrinolysis on admission measured via thromboelastography. Hyperfibrinolysis was defined as Ly30 ≥3% on thromboelastography<br><br>**Exclusion criteria**<br>• all patients who received TXA >3 hours of injury<br><br>**Characteristics**<br><br>Age: mean, ± SD<br>IG: 42.5 ± 20 (p=0.33)<br>CG: 38.7 ± 17<br><br>Male gender: % §<br>IG: 66% (p=0.84)<br>CG: 68%<br><br>Injury Severity Score (ISS): median (IQR)<br>IG: 38 (23 - 45) (p=0.56)<br>CG: 35 (21 - 45)<br><br>GCS: median, IQR)<br>IG: 6 (3 - 15) (p=0.34)<br>CG: 8 (3 - 15)<br><br>SBP: median (IQR)<br>IG: 90 (70 - 126) (p=0.28)<br>CG: 101 (80 - 131)<br><br>Lactate: median (IQR)<br>IG: 8.3 (5.1 - 11.7) (p=0.83)<br>CG: 9.5 (5.1 - 12.7)<br><br>Transfusion ratio (1:1:1): %§	N=93 patients, matched in 1:2 ratio<br><br>(117 patients pre-matching)<br><br>**Study groups**<br>IG: TXA (N=31)<br><br>CG: no TXA (N=62)<br><br>**Matching criteria**<br>Propensity score matching according to age, gender, race, ED SBP, ED HR, mechanism of injury, ISS, head-AIS, GCS, and PROPPR intervention groups (1:1:1 or 1:1:2 transfusion ratios).	6-hour: %§<br>IG: 13 (p=0.04)<br>CG: 34<br><br>24-hour: %§<br>IG: 26 (p=0.25)<br>CG: 39<br><br>30-day: %§<br>IG: 45 (p=0.82)<br>CG: 50<br><br>**Cause of death§**<br><br>Exsanguination/Hemorrhagic shock: %§<br>IG: 26 (p=0.39)<br>CG: 32<br><br>TBI: %§<br>IG: 10 (p=0.62)<br>CG: 13<br><br>Respiratory: %§<br>IG: 6.4 (p=0.26)<br>CG: 1.6<br><br>Other: %§<br>IG: 3.2 (p=1.00)<br>CG: 3.2<br><br>**Complications§** - *Secondary outcomes*<br><br>Deep venous thrombosis: %<br>IG: 6.5 (p=0.59)<br>CG: 3.2<br><br>Acute Kidney Injury (AKI): %	2b<br><br>**Risk of bias**<br>Selection bias: +<br><br>Performance bias: +<br><br>Attrition bias: +<br><br>Detection bias: +<br><br>**Authors' conclusion**<br>"Tranexamic acid (TXA) was associated with increased 6 hour survival but does not improve long term outcomes in severely injured trauma patients with hemorrhage who develop hyperfibrinolysis."<br><br>**Reviewers' conclusion**<br>There may be a risk of performance bias because TXA use was not prescribed in the PROPPR study protocol and left to the discretion of the trauma attending. However, patients received the same care apart from TXA use and cohorts were matched according to confounders and transfusion ratios showing consistent results.

Study: Reference, aim, design, setting	Participants: selection criteria, characteristics	N Participants; Intervention (IG) vs. Control group (CG)	Main outcomes	Assessment: LoE, risk of bias; Conclusions
	IG: 55%<br>CG: 47%<br>§ n=total number of patients not reported		IG: 45 (p=0.01)<br>CG: 19<br><u>Sepsis: %</u><br>IG: 35 (p=0.04)<br>CG: 16<br><u>Multiple organ failure: %</u><br>IG: 19 (p=0.01)<br>CG: 6.4<br><u>ICU free days: median (IQR)</u><br>IG: 0 (0 – 3) (p=0.22)<br>CG: 0 (0–5)<br>§ n=number of patients with event not reported	
**Meizoso (2018)**<br>"Increased risk of fibrinolysis shutdown among severely injured trauma patients receiving tranexamic acid". *Journal of Trauma and Acute Care Surgery* 84(3), 426 - 432.<br>**Study design**<br>Prospective cohort study<br>(subanalysis)<br>**Aim of the study**<br>"The aim of this study was to test the hypothesis that TXA administration is associated with fibrinolysis shutdown in a	**Inclusion criteria**<br>• Adult trauma patients [1]<br>• enrolled in a prospective observational trial of postinjury coagulation changes in severely injured patients in the trauma intensive care unit (ICU)<br>• thromboelastography (TEG) drawn upon ICU admission<br>**Exclusion criteria**<br>• burn injury<br>• pregnancy<br>• incarceration<br>**Characteristics**<br><u>Age (years, mean ± SD)</u><br>IG: 46 ± 15 vs. CG: 46 ± 19 (p=0.892)<br><u>Sex (male / female, n (%))</u>	**Participants**<br>N=218 patients<br>**Study groups**<br>IG: Tranexamic acid (TXA) (N=35, 16%): 1-g bolus over 10 minutes followed by 1 g infusion over 8 hours at the discretion of the trauma surgeons and anesthesiologists within 3 hours after injury.<br>CG: No TXA (N=183, 84%)	**<u>ICU-free days, median (IQR)</u>**<br>IG: 16 (4–55) vs. CG: 10 (4–19) (p=0.060)<br><u>Hospital LOS (days), median (IQR)</u><br>IG: 41 (23–92) vs. CG: 25 (15–44) (p=0.015)<br><u>Mortality, n (%)</u><br>IG: 6 (17.1) vs. CG: 27 (14.8) (p=0.718)<br>**Fibrinolysis phenotype, n (%)**<br><u>Shutdown</u><br>IG: 32 (91.4) vs. CG: 107 (58.5) (p<0.0001)<br><u>Physiologic</u><br>IG: 3 (8.6) vs. CG: 68 (37.2) (p=0.001)<br><u>Hyperfibrinolysis</u><br>IG: 0 (0) vs. CG: 8 (4.4) (p=0.208)<br>**Regression analysis**	**Level of evidence**<br>3b↓<br>**Risk of bias**<br>Selection bias: –<br>Performance bias: ?<br>Attrition bias: +<br>Detection bias: +<br>**Authors' conclusion**<br>"Patients who received TXA were at increased risk of fibrinolysis shutdown compared with patients who did not receive TXA. We recommend that administration of TXA be limited to severely injured patients with evidence of

Study: Reference, aim, design, setting	Participants: selection criteria, characteristics	N Participants; Intervention (IG) vs. Control group (CG)	Main outcomes	Assessment: LoE, risk of bias; Conclusions
group of high-risk trauma patients."<br><br>**Setting**<br><br>USA, 2011-2015	IG: 27 (77.1) / 8 (22.9) vs.<br>CG: 148 (81.3) / 34 (18.7) (p=0.567)<br><br><u>Admission SBP (mmHg, mean ± SD)</u><br><br>IG: 98 ± 32 vs. CG: 123 ± 41 (p=0.001)<br><br><u>Admission Shock Index (mean ± SD)</u><br><br>IG: 1.23 ± 0.60 vs. CG: 0.90 ± 0.41 (p<0.0001)<br><br><u>Admission GCS (median, IQR)</u><br><br>IG: 9 (4–15) vs. CG: 14 (6–15) (p=0.074)<br><br><u>ISS (mean ±SD)</u><br><br>IG: 30 ± 15 vs. CG: 28 ± 13 (p=0.377)<br><br><u>ISS >15[1] (n (%))</u><br><br>IG: 30 (85.7) vs. CG: 150 (82.0) (p=0.592)<br><br><u>Massive Transfusion , n (%)</u><br><br>IG: 23 (65.7) vs. CG: 31 (18.9) (p<0.0001)<br><br><br>[1] CAVE: 14.3% / 18% are ISS <15		<u>Independent predictors of fibrinolysis shutdown (adjusted RR, 95% CI)</u><br><br>TXA: 1.35 (1.10–1.64) (p=0.004)<br><br>Cryoprecipitate: 1.29 (1.07–1.56) (p=0.007)	hyperfibrinolysis and recommend caution in those with evidence of fibrinolysis shutdown."<br><br>**Reviewers' conclusion**<br><br>There may be a high risk of selection bias. The groups differ in baseline factors.
**Moore (2017)**<br><br>"Tranexamic acid is associated with increased mortality in patients with physiological fibrinolysis ", *Journal of Surgical Research* 2017; 220: 438-443.<br><br>**Study design**<br><br>Prospective cohort study	**Inclusion criteria**<br><br>• Adult trauma patients (aged >18 years)<br>• highest level of activation at level I trauma center<br>• new injury severity score (NISS) >15<br><br>**Characteristics**<br><br><u>Age [y], median (IQR)</u><br><br>IG: 27 (24-54), p=0.214<br>CG: 34 (27-49)	**Participants**<br><br>N=232 patients<br><br>**Study groups**<br><br>IG: TXA (N=26)<br><br>CG: no TXA (N=206)	**Mortality**<br><br><u>Mortality (in-hospital): %[§]</u><br><br>IG: 50, p<0.001<br>CG: 17<br><br>*Mortality (in-hospital) by phenotype*<br><br><u>Hyperfibrinolysis, %</u><br><br>IG: 56, p=0.023<br>CG: 19	**Level of evidence**<br><br>2b<br><br>**Risk of bias**<br><br>Selection bias: –<br><br>Performance bias: –<br><br>Attrition bias: +<br><br>Detection bias: +<br><br>**Authors' conclusion**

Study: Reference, aim, design, setting	Participants: selection criteria, characteristics	N Participants; Intervention (IG) vs. Control group (CG)	Main outcomes	Assessment: LoE, risk of bias; Conclusions
**Aim of the study**<br>"The aim of the study was to investigate if TXA in patients with a physiological level of fibrinolysis will have an increase in mortality compared with other fibrinolytic phenotypes."<br>**Setting:**<br>USA, 2014-2016	<u>Male gender, %[§]</u><br>IG: 85, p=0.362<br>CG: 77<br><u>NISS, median (IQR)</u><br>IG: 48 (29-57), p=0.001<br>CG: 29 (22-43)<br><u>INR, median (IQR)</u><br>IG: 1.4 (1.2-1.8), p<0.001<br>CG: 1.2 (1.1-1.3)<br>**Fibrinolysis phenotype (n)**<br><u>Hyperfibrinolysis, n (N=64)</u><br>IG: 10<br>CG: 54<br><u>Shutdown, n (N=54)</u><br>IG: 8<br>CG: 46<br><u>Physiologic, n (N=114)</u><br>IG: 8<br>CG: 106<br>[§] n=number of patients with event not reported		<u>Shutdown, %</u><br>IG: 38, p=0.604<br>CG: 28<br><u>Physiologic, %</u><br>IG: 63, p<0.001<br>CG: 11<br><u>Death associated with haemorrhage[§] (%)</u><br>IG: 55% (p=0.060)<br>CG: 23%<br><u>TXA as predictor of mortality by fibrinolysis phenotype (adj. for NISS)</u><br>Physiologic (p=0.018)<br>Hyperfibrinolysis (p=0.116)<br>Shutdown (p=0.597)<br><u>Massive transfusion: %[§]</u><br>IG: 69, p<0.001<br>CG: 12<br>[§] n=number of patients with event not reported	"There was no clear benefit of receiving TXA in this study, and patients who present to the hospital with physiologic levels of fibrinolysis, who received TXA, had the highest mortality."<br>**Reviewers' conclusion**<br>There is a substantial risk of selection bias due to imbalance of NISS and INR. Blinding was unclear and co-interventions were different (patients in the TXA group tended to receive more blood products), so that there is a risk for performance bias. The risk of attrition bias is unclear because the numbers in the analyses were not reported.
**Nishijima (2019)**<br>"The Effect of Tranexamic Acid on Functional Outcomes: An Exploratory Analysis of the CRASH-2 Randomized Controlled Trial". *Annals of*	**Inclusion criteria**<br>• Same as in CRASH-2 trial and<br>• only patients randomised 3 hours or less from the time of injury<br>**Exclusion criteria**	**Participants**<br>N=13,432 patients, subset of CRASH-2 dataset<br>**Study groups**<br>IG: TXA (N=6,753)<br>CG: Placebo (N=6,679)	**Modified Oxford Handicap Scale score (at discharge or at 28 days): n (%)**<br><u>No symptoms</u><br>IG: 1,052 (15.6)<br>CG: 941 (13.9)<br><u>Minor symptoms</u>	**Level of evidence**<br>1b<br>**Risk of bias**<br>Selection bias: +<br>Performance bias: +

Study: Reference, aim, design, setting	Participants: selection criteria, characteristics	N Participants; Intervention (IG) vs. Control group (CG)	Main outcomes	Assessment: LoE, risk of bias; Conclusions
Emergency Medicine. 74(1), 79-87<br>**Study design**<br>Randomised controlled trial (exploratory analysis of CRASH-2 trial data)<br>**Aim of the study**<br>The aim of the study was to evaluate whether tranexamic acid was associated with improved functional outcomes and, if so, which patients benefitted from tranexamic acid use.<br>**Setting**<br>40 countries, 2005-2010	• patients who did not have modified Oxford Handicap Scale scores reported<br>**Characteristics**<br><u>Age (mean ± SD)</u><br>IG: 34.1 (± 13.8)<br>CG: 34.1 (± 14.2)<br><u>Male sex: n (%))</u><br>IG: 5,605 (83.0)<br>CG: 5,606 (84.0)<br><u>Initial GCS: median (IQR)</u><br>IG: 12.7 (3.6)<br>CG: 12.7 (3.6)<br>**Baseline risk of mortality stratum: n %)§**<br><u><6</u><br>IG: 2,415 (35.8)<br>CG: 2,325 (34.9)<br><u>6-20</u><br>IG: 2,410 (35.7)<br>CG: 2,391 (35.9)<br><u>21- 50</u><br>IG: 1,171 (17.4)<br>CG: 1,201 (18.0)<br><u>>50</u><br>IG: 753 (11.2)<br>CG: 752 (11.3)<br><u>Days in hospital (median, IQR)</u>		IG: 2,190 (32.4)<br>CG: 2,140 (32.0)<br><u>Some restrictions</u><br>IG: 1,311 (19.4)<br>CG: 1,324 (19.8)<br><u>Dependent</u><br>IG: 807 (11.9)<br>CG: 779 (11.7)<br><u>Fully dependent</u><br>IG: 421 (6.2)<br>CG: 396 (5.9)<br><u>Dead</u><br>IG: 972 (14.4)<br>CG: 1,109 (16.6)<br><u>mean utility-weighted modified Oxford Handicap Scale score: mean ± SD</u><br>IG: 0.66 (± 0.33)<br>CG: 0.64 (± 0.34)<br>mean difference = 0.02 (95% CI 0.01 – 0.03) (p<0.001)<br><u>28-day mean utility-weighted modified Oxford Handicap Scale score (Area under the curve analysis): mean ± SD:</u><br>IG: 0.55 (± 0.30)<br>CG: 0.53 (± 0.31)<br>mean difference = 0.02 (95% CI 0.01 – 0.03)<br>**functional outcomes, stratified by CRASH-2 prognostic score: n (%) (95% CI) §**<br><u>0-6 % baseline risk</u>	Attrition bias: +<br>Detection bias: +<br>**Authors' conclusion**<br>"In this exploratory analysis we found that adult trauma patients randomized to tranexamic acid within 3 hours of injury had better functional outcomes compared with patients randomized to placebo."<br>**Reviewers' conclusion**<br>The trial is of good quality indicating reliable results.

Study: Reference, aim, design, setting	Participants: selection criteria, characteristics	N Participants; Intervention (IG) vs. Control group (CG)	Main outcomes	Assessment: LoE, risk of bias; Conclusions
	IG: 7 (3–14)<br>CG: 7 (3–14)		Overall favorable outcome (no symptoms)<br><br>IG: 534 (22.1) (95% CI 20.5 – 23.8)<br>CG: 425 (18.3) (95% CI 16.7 – 19.9)<br><br>IG vs. CG: adjusted OR for favorable outcome = 1.28 (95% CI 1.11 – 1.48)<br><br><u>6-20 % baseline risk</u><br>Overall favorable outcome (no or minor symptoms)<br><br>IG: 1209 (50.2) (95% CI 48.1 – 52.2)<br>CG: 1202 (50.3) (95% CI 48.2 – 52.3)<br><br>IG vs. CG: adjusted OR for favorable outcome = 0.99 (95% CI 0.88 – 1.11)<br><br><u>21-50 % baseline risk</u><br>Overall favorable outcome (no or minor symptoms or some restrictions)<br><br>IG: 611 (52.2) (95% CI 49.3 – 55.1)<br>CG: 588 (49.0) (95% CI 46.1% - 51.8)<br><br>IG vs. CG: adjusted OR for favorable outcome = 1.15 (95% CI 0.97 – 1.37)<br><br><u>>50 % baseline risk</u><br>Overall favorable outcome (no or minor symptoms or some restrictions or dependent)<br><br>IG: 238 (31.6) (95% CI 28.3 – 35.1)<br>CG: 217 (28.9) (95% CI 25.6 – 32.2)<br><br>IG vs. CG: adjusted OR for favorable outcome = 1.24 (95% CI 0.97 – 1.57)<br><br><u>Overall proportion of patients with favorable outcomes: n (%) (95% CI):</u>	

Study: Reference, aim, design, setting	Participants: selection criteria, characteristics	N Participants; Intervention (IG) vs. Control group (CG)	Main outcomes	Assessment: LoE, risk of bias; Conclusions
			IG: 5,360 (79.4) (95% CI 78.4% - 80.3)<br>CG: 5,174 (77.) (95% CI 76.5% - 78.5)<br>difference 1.9% (95% CI 0.5% - 3.3)<br>NNT = 52 (95% CI 30 – 196)<br><br>§ Favorable versus unfavorable outcomes were defined separately for each risk stratum	
**Roberts (2014)**<br>"Mechanism of action of tranexamic acid in bleeding trauma patients: an exploratory analysis of data from the CRASH-2 trial." *Critical Care* 2014; 18(6): 1-5.<br>**Study design**<br>Randomised controlled trial (CRASH-2)<br>**Aim of the study**<br>“We conducted further analyses of the CRASH-2 trial data to examine the timing of the effect of TXA on mortality.”<br>**Setting**<br>40 countries, 2005-2010	Same as *CRASH-2:*<br>**Inclusion criteria**<br>• adult trauma patients with, or at risk of, significant bleeding, and<br>• who were within 8 h of their injury<br>**Characteristics**<br>no patient characteristics reported	**Participants**<br>N=20,211 patients<br>**Study groups**<br>IG: TXA (loading dose 1 g over 10 minutes followed by an infusion of 1 g over 8 h) (N=10,060 with outcome data)<br>CG: matching placebo (N=10,067 with outcome data)	**All cause mortality (incl. non-bleeding patients!)**<br>0 days since injury, Hazard Ratio (95% CI)<br>0.83 (0.73, 0.93)<br>1 day since injury, Hazard Ratio (95% CI)<br>0.91 (0.79, 1.04)<br>2 days since injury, Hazard Ratio (95% CI)<br>0.96 (0.77, 1.19)<br>3 days since injury, Hazard Ratio (95% CI)<br>1.01 (0.76, 1.34)<br>4 days since injury, Hazard Ratio (95% CI)<br>0.96 (0.70, 1.36)<br>**Mortality due to bleeding**<br>0 days since injury, Hazard Ratio (95% CI)<br>0.80 (0.68, 0.94)<br>1 day since injury, Hazard Ratio (95% CI)<br>0.89 (0.72, 1.11)<br>2 days since injury, Hazard Ratio (95% CI)<br>1.17 (0.74, 1.86)	**Level of evidence**<br>1b<br>**Risk of bias**<br>Selection bias: +<br>Performance bias: +<br>Attrition bias: +<br>Detection bias: +<br>**Authors’ conclusion**<br>“Early administration of tranexamic acid appears to reduce mortality primarily by preventing exsanguination on the day of the injury.”<br>**Reviewers’ conclusion**<br>It is unclear if the analysis was predefined. Apart from that, this subgroup analyses of the CRASH-2 trial is of good quality indicating reliable results.

Study: Reference, aim, design, setting	Participants: selection criteria, characteristics	N Participants; Intervention (IG) vs. Control group (CG)	Main outcomes	Assessment: LoE, risk of bias; Conclusions
			<u>3 days since injury, Hazard Ratio (95% CI)</u><br>0.66 (0.32, 1.37)<br><u>4 days since injury, Hazard Ratio (95% CI)</u><br>0.77 (0.29, 2.06)<br>**Non-bleeding mortality**<br><u>0 days since injury, Hazard Ratio (95% CI)</u><br>0.87 (0.71, 1.06)<br><u>1 day since injury, Hazard Ratio (95% CI)</u><br>0.92 (0.76, 1.11)<br><u>2 days since injury, Hazard Ratio (95% CI)</u><br>0.91 (0.71, 1.16)<br><u>3 days since injury, Hazard Ratio (95% CI)</u><br>1.09 (0.80, 1.48)<br><u>4 days since injury, Hazard Ratio (95% CI)</u><br>1.01 (0.71, 1.43)<br>**All cause mortality, Time to treatment <u>≤3h</u> (incl. non-bleeding patients!)**<br><u>0 days since injury, Hazard Ratio (95% CI)</u><br>0.78 (0.68, 0.90)<br><u>1 day since injury, Hazard Ratio (95% CI)</u><br>0.86 (0.72, 1.02)<br><u>2 days since injury, Hazard Ratio (95% CI)</u><br>0.86 (0.65, 1.13)<br><u>3 days since injury, Hazard Ratio (95% CI)</u><br>0.95 (0.66, 1.37)	

Study: Reference, aim, design, setting	Participants: selection criteria, characteristics	N Participants; Intervention (IG) vs. Control group (CG)	Main outcomes	Assessment: LoE, risk of bias; Conclusions
			<u>4 days since injury, Hazard Ratio (95% CI)</u><br>0.94 (0.61, 1.45)<br>**All cause mortality, time to treatment <u>>3h</u> (incl. non-bleeding patients!)**<br><u>0 days since injury, Hazard Ratio (95% CI)</u><br>1.02 (0.76, 1.36)<br><u>1 day since injury, Hazard Ratio (95% CI)</u><br>1.02 (0.80, 1.31)<br><u>2 days since injury, Hazard Ratio (95% CI)</u><br>1.16 (0.81, 1.66)<br><u>3 days since injury, Hazard Ratio (95% CI)</u><br>1.11 (0.73, 1.71)<br><u>4 days since injury, Hazard Ratio (95% CI)</u><br>1.04 (0.62, 1.75)	
**Roberts (2017)**<br>"Tranexamic acid in bleeding trauma patients: an exploration of benefits and harms." *Trials* 18: 48<br>**Study design**<br>Randomised controlled trial (predefined subgroup analysis of CRASH-2)<br>**Aim of the study**<br>We examine how patient characteristics vary by time to treatment in the	Same as *CRASH-2:*<br>**Inclusion criteria**<br>• Adult trauma patients<br>• with, or at risk of, significant bleeding within 8 h of their injury<br>**Characteristics**<br>no patient characteristics reported	**Participants**<br>N=20,211 patients<br>**Study groups**<br>IG: TXA (loading dose 1 g over 10 min followed by an infusion of 1 g over 8 h) (N=10,093)<br><br>CG: matching placebo (N=10,114)	**Subgroup analyses of CRASH-2**<br>• SBP (≤75, 76–89, >89 mmHg)<br>• GCS score (severe 3–8, moderate 9–12, mild 13–15)<br>• type of injury (penetrating versus blunt)<br><u>1. Effects of **early** tranexamic acid (TXA) treatment stratified by systolic blood pressure on death due to bleeding: Risk Ratio (95% CI)</u><br>SBP ≤75 RR: 0.73 (0.61-0.86)<br>SBP 76-89 RR: 0.86 (0.64-1.16)<br>SBP>89 RR: 0.71 (0.54-0.92)<br>SBP <100 mg and treatment initiated within 1h	**Level of evidence**<br>1b<br>**Risk of bias**<br>Selection bias: +<br>Performance bias: +<br>Attrition bias: +<br>Detection bias: +<br>**Authors' conclusion**<br>"When given within 3 h of injury, TXA reduces death due to bleeding regardless

Study: Reference, aim, design, setting	Participants: selection criteria, characteristics	N Participants; Intervention (IG) vs. Control group (CG)	Main outcomes	Assessment: LoE, risk of bias; Conclusions
CRASH-2 trial and explore whether any such variations explain the time-dependent treatment effect.<br><br>**Setting**<br><br>40 countries, 2005-2010			RR = 0.69 (0.58 - 0.83)<br><br>SBP <100 mg and treatment between 1-3h<br><br>RR = 0.84; 95% (0.67 - 1.04)<br><br>Effects of **late** tranexamic acid (TXA) treatment stratified by **systolic blood pressure** (SBP) on death due to bleeding: Risk Ratio (95% CI)<br><br>Systolic blood pressure (mm Hg) ≤75,<br><br>1.36 (0.92-2.01)<br><br>2. Effects of **early** tranexamic acid (TXA) treatment stratified by **Glasgow Coma Scale** (GCS) score on death due to bleeding: Risk Ratio (95% CI)<br><br>GCS 3-8 RR: 0.82 (0.66-1.02)<br><br>Effects of **late** tranexamic acid (TXA) treatment stratified by **Glasgow Coma Scale** (GCS) score on death due to bleeding: Risk Ratio (95% CI)<br><br>GCS 3-8<br><br>1.42 (0.90-2.25)<br><br>3. Effects of **early** tranexamic acid (TXA) treatment stratified by **type of injury** on death due to bleeding: Risk Ratio (95% CI)<br><br>Blunt 0.72 (0.60 - 0.86)<br><br>Penetrating 0.73 (0.60 – 0.90)<br><br>Effects of **late** tranexamic acid (TXA) treatment stratified by **type of injury** on death due to bleeding: Risk Ratio (95% CI)<br><br>Blunt 1.48 (1.12 – 1.96)<br><br>Penetrating 1.25 (0.74 – 2.12)	of injury type, GCS or blood pressure."<br><br>**Reviewers' conclusion**<br><br>This predefined subgroup analyses of the CRASH-2 trial is of good quality indicating reliable results.

Study: Reference, aim, design, setting	Participants: selection criteria, characteristics	N Participants; Intervention (IG) vs. Control group (CG)	Main outcomes	Assessment: LoE, risk of bias; Conclusions
**Shiraishi (2017)**<br>„Effectiveness of early administration of tranexamic acid in patients with severe trauma". *British Journal of Surgery* 104: 710-717.<br>**Study design**<br>Comparative registry study<br>(Japanese Observational Study for Coagulation and Thrombolysis in Early Trauma)<br>**Aim of the study**<br>The aims of the present study were to compare 28-day mortality and blood transfusion amounts among severely injured subjects who did or did not receive tranexamic acid within 3 h of injury, based on propensity score matching that balanced for background characteristics including ISS and indicators of coagulopathy and fibrinolysis.<br>**Setting**	**Inclusion criteria**<br>• patients aged ≥18 years<br>• ISS ≥16<br>• Admitted to one of the study hospitals<br>**Exclusion criteria**<br>• complications such as out-of-hospital cardiac arrest<br>• burns<br>• liver cirrhosis<br>• isolated cervical spine injury not caused by a high-energy accident<br>• pregnant<br>**Characteristics (after matching)**<br><u>Age (median, IQR)</u><br>IG: 57 (36–72) (SMD = −0.038)<br>CG: 56 (38–69)<br><u>Gender (male, n (%))</u><br>IG: 181 (72.4) (SMD = −0.065)<br>CG: 186 (74.4)<br><u>Systolic BP (mmHg, median, IQR)</u><br>IG: 136 (110 - 159) (SMD = 0.031)<br>CG: 133 (110 - 157)<br><u>GCS (median, IQR)</u><br>IG: 13 (8 - 15) (SMD = −0.004)<br>CG: 13 (8 - 15)<br><u>Injury Severity Score (ISS) (median, IQR)</u><br>IG: 25 (17 - 29) (SMD = −0.007)<br>CG: 25 (17 - 29)	**Participants**<br>N=500 after matching<br>**Study groups**<br>IG: TXA (N=250), IV administration within 3h after injury<br>CG: No TXA (N=250 propensity score-matched controls)<br>**Matching criteria**<br>1:1 Propensity score matching<br>Before matching: N=281 TXA, n=525 control patients.<br>Logistic regression analysis was employed to compute the propensity score for the use of the study intervention in each subject from the known pretreatment variables that were considered to be associated clinically with the primary outcome of the study.	<u>28-day mortality§ (%) (mean difference, (95% CI):</u><br>IG: 10.0 (−8.4 (−14.5, −2.3))<br>CG: 18.4<br>Odds Ratio (95% CI) = 0.49 (0.29, 0.83)<br><u>Cause-specific mortality§ (%):</u><br>Primary brain injury<br>IG: 6.0 (−7.2 (−12.3, −2.1))<br>CG: 13.2<br>Odds Ratio (95% CI) = 0.42 (0.22, 0.88))<br><u>Haemorrhage</u><br>IG: 2.8 (−1.2 (−4.4, 2.0))<br>CG: 4.0<br>Odds Ratio (95% CI) = 0.69 (0.26, 1.85)<br><u>Thromboembolic complications§ (%):</u><br>IG: 1.2 (−0.8 (−3.0, 1.4))<br>CG: 2.0<br>Odds Ratio (95% CI) = 0.60 (0.14, 2.53)<br><br>§ N=number of patients with event not reported	**Level of evidence**<br>2b<br>**Risk of bias**<br>Selection bias: +<br>Performance bias: ?<br>Attrition bias: +<br>Detection bias: +<br>**Authors' conclusion**<br>"Early tranexamic acid use was associated with reduced mortality in severely injured patients, in particular those with a primary brain injury."<br>**Reviewers' conclusion**<br>Precautions were taken to minimise biases. The intervention and control groups were comparable with respect to baseline characteristics; co-interventions are not reported.

Study: Reference, aim, design, setting	Participants: selection criteria, characteristics	N Participants; Intervention (IG) vs. Control group (CG)	Main outcomes	Assessment: LoE, risk of bias; Conclusions
Japan, 2012	<u>Traumatic Brain Injury (n, (%))</u><br>IG: 188 (75.2) (SMD = −0.019)<br>CG: 190 (76.0)<br><u>Lactate (mmol/l, median, IQR)</u><br>IG: 2.6 (1.7 - 4.0) (SMD = −0·007)<br>CG: 2.6 (1.6 - 3.9)<br>SMD = Standardized Mean Difference			
**Spinella (2020)**<br>"The immunologic effect of early intravenous two and four gram bolus dosing of tranexamic acid compared to placebo in patients with severe traumatic bleeding (TAMPITI): A randomized, double-blind, placebo-controlled, single-center trial". *Frontiers in Immunology* 11: 2085.<br>**Study design**<br>Randomised controlled trial<br>**Aim of the study**<br>The hemostatic properties of tranexamic acid (TXA) are well described, but the immunological effects of TXA administration after traumatic injury have not been thoroughly examined. We	**Inclusion criteria**<br>• Age ≥18<br>• sustained a traumatic injury which required them to receive at least one unit of red blood cells (RBC) or required an emergent operation for possible bleeding control<br>• were able to receive the study medication (TXA or placebo) within 2 h of time of injury<br>**Exclusion criteria**<br>• Suspected acute MI or stroke (thromboembolic and/or hemorrhagic) on admission<br>• Known inherited coagulation disorders<br>• Known past medical history of thromboembolic events (DVT, PE, MI, Thromboembolic Stroke)<br>• Known history of seizures and/or seizure after injury/on admission related to this hospitalization<br>• Suspected or known pregnancy<br>• Futile care	**Participants**<br>N=150 patients<br>**Study groups**<br>TXA 2g: 2 g of TXA (N=49)*<br>TXA 4g: 4 g of TXA (N=50)<br>CG: placebo (N=50)<br>each in 40 mL of normal saline i.v. over 10 min<br><br>* 1 patient withdrawn (age <18 yrs)	<u>28-day mortality n/N, (%)</u><br>CG: 6/49 (12.2) vs.<br>TXA 2g: 5/44 (11.4) vs.<br>TXA 4g: 4/48 (8.33), p=0.8<br><u>Thromboembolic event n/N, (%)</u><br>CG: 6/50 (12.0) vs.<br>TXA 2g: 13/49 (26.5) vs.<br>TXA 4g: 16/50 (32.0), p=0.05<br><u>ICU admission n/N, (%)</u><br>CG: 37/50 (74.0) vs.<br>TXA 2g: 36/49 (73.5) vs.<br>TXA 4g: 38/50, p=0.96<br><u>Mechanical ventilation n/N, (%)</u><br>CG: 30/50 (60.0) vs.<br>TXA 2g: 28/48 (58.3) vs.<br>TXA 4g: 30/49 (61.2), p=0.96<br><u>ICU-free Days, [N] median (IQR)</u><br>CG: [50] 27.3 (17.4 – 28.6) vs.<br>TXA 2g: [45] 27.1 (24.0 – 29.4) vs.<br>TXA 4g: [49] 27.1 (24.3 – 29.0), p=0.77<br><u>Max MODS in 7 days , [N] median (IQR)</u>	**Level of evidence**<br>2b↓<br>**Risk of bias**<br>Selection bias: +<br>Performance bias: +<br>Attrition bias: +<br>Detection bias: +<br>**Authors' conclusion**<br>"In conclusion, in this RCT in patients with primarily penetrating traumatic injuries, 2 and 4 g i.v. bolus dosing of TXA had minimal immunomodulatory and hemostatic effects."<br>**Reviewers' conclusion**<br>The clinical outcomes (mortality, morbidity) were secondary outcomes.<br>The RCT was not powered

Study: Reference, aim, design, setting	Participants: selection criteria, characteristics	N Participants; Intervention (IG) vs. Control group (CG)	Main outcomes	Assessment: LoE, risk of bias; Conclusions
hypothesized TXA would reduce monocyte activation in bleeding trauma patients with severe injury.<br>**Setting**<br>USA, 2016-2017	• Known current state of immunosuppression (i.e. on high dose steroids, chemotherapeutics, etc.)<br>• Unknown estimated time of injury<br>• Patients wearing an "Opt Out" TAMPITI Study bracelet<br>• Known presence of subarachnoid hemorrhage<br>• Isolated injuries to hands and/or feet (distal)<br>• Administration of antifibrinolytics pre-hospital and/or during this ED admission prior to enrollment<br>**Characteristics**<br><u>Age [y], median (IQR)</u><br>CG: 27.0 (22.0 – 34.0) vs.<br>TXA 2g: 26.0 (22.0 – 40.0) vs.<br>TXA 4g: 31.0 (25.0 – 44.0), p=0.13<br><u>Male n (%)</u><br>CG: 45 (90.0) vs.<br>TXA 2g: 44 (90.0) vs.<br>TXA 4g: 42 (84.0), p=0.58<br><u>GCS, [n] median (IQR)</u><br>CG: 15.0 (12.0 – 15.0) vs.<br>TXA 2g: 15.0 (11.0 – 15.0) vs.<br>TXA 4g: 15.0 (14.0 – 15.0), p=0.26		CG: [49] 4.00 (1.00 – 7.00) vs.<br>TXA 2g: [49] 4.00 (1.00 – 6.00) vs.<br>TXA 4g: [50] 4.00 (1.00 – 8.00), p=0.79<br><u>Seizure n/N, (%)</u><br>CG: 0/49 (0.00) vs.<br>TXA 2g: 1/44 (2.27) vs.<br>TXA 4g 2/48 (4.17), p=0.42<br>MODS = multiple organ dysfunction score	to detect differences in clinical outcomes.
**Wafaisade (2016)**<br>"Prehospital administration of tranexamic acid in trauma	**Inclusion criteria**<br>ADAC Air Rescue Service database:<br>• Primarily admitted trauma patient<br>• Critical injury, defined as preclinically assessed NACA IV (potentially life-	**Study groups**<br>IG: prehospital TXA (N=258)<br>CG: no prehospital TXA (N=258 propensity score-matched controls)	<u>Time to death, days, mean ± SD</u><br>IG: 8.8 (13.4) vs. CG: 3.6 (4.9), p=0.001<br><u>6-h mortality, n (%)</u><br>IG: 5/258 (1.9) vs. CG: 24/258 (9.3), p<0.001	**Level of evidence**<br>2b<br>**Risk of bias**<br>Selection bias: +

Study: Reference, aim, design, setting	Participants: selection criteria, characteristics	N Participants; Intervention (IG) vs. Control group (CG)	Main outcomes	Assessment: LoE, risk of bias; Conclusions
patients". *Critical Care* 20(1): 143.<br>**Study design**<br>Comparative registry study<br>(TraumaRegister DGU®)<br>**Aim of the study**<br>the aim of the present study was to assess whether prehospital intravenous (i.v.) administration of TXA in trauma patients is associated with improved outcomes.<br>**Setting**<br>Germany, 2012-2014	threatening), NACA V (acute danger) or NACA VI (respiratory and/or cardiac arrest)<br>• Admission to a trauma centre participating in the TR-DGU<br>TR-DGU database:<br>• Primary admission<br>• Treatment in a German trauma centre (i.e., exclusion of trauma centres from other countries)<br>**Characteristics**<br>Age, years, mean ± SD<br>IG: 43 ± 19 vs. CG: 41 ± 18, p=0.48<br>Male, sex, n (%)<br>IG: 187 (72.5) vs. CG: 187 (72.5), p=1.00<br>ISS, points, mean ± SD<br>IG: 24 ± 14 vs. CG: 24 ± 16, p=0.46<br>SBP at scene ≤90 mmHg, n (%)<br>IG: 55 (21.3) vs. CG: 54 (20.9), p=1.0<br>SBP at scene, mmHg, mean ± SD<br>IG: 118 ± 34 vs. CG: 116 ± 33, p=0.36<br>GCS at scene ≤8, n (%)<br>IG: 89 (34.5) vs. CG: 96 (37.2), p=0.58<br>GCS at scene, points, mean ± SD<br>IG: 10.5 ± 4.9 vs. CG: 10.2 ± 5.0, p=0.69	**Matching criteria**<br>Propensity score matching<br>Multivariable analysis using a logistic regression model with prehospital administration of tranexamic acid as a dependent variable (N=5765)	12-h mortality, n (%)<br>IG: 9/258 (3.5) vs. CG: 28/258 (10.9), p=0.002<br>24-h mortality, n (%)<br>IG: 15/258 (5.8) vs. CG: 32/258 (12.4), p=0.01<br>30-day mortality, n (%)<br>IG: 36/258 (14.0) vs. CG: 42/258 (16.3), p=0.54<br>In-hospital mortality overall, n (%)<br>IG: 38/258 (14.7) vs. CG: 42/258 (16.3), p=0.72<br>Mortality prognosis in %, based on RISC 2 score (n)<br>IG: 15.4% (258) vs. CG: 15.2% (258), p=0.38<br>ICU LOS, days, mean ± SD<br>IG: 10.7 ± 12.6 vs. CG: 9.2 ± 11.4, p=0.03<br>Hospital LOS, days, mean ± SD<br>IG: 25.5 ± 23.2 vs. CG: 22.3 ± 25.4, p=0.04<br>Thromboembolic event, n (%)$<br>IG: 4/71 (5.6) vs. CG: 10/121 (8.3), p=0.58<br>Sepsis, n (%)$<br>IG: 4/67 (6.0) vs. CG: 8/119 (6.7), p=1.00<br>Multiple organ failure, n (%)$<br>IG: 27/74 (36.5) vs. CG: 35/121 (28.9), p=0.34<br>$ As some values were missing, the respective population is documented in brackets for continuous variables and in the denominator for categorical variables.	Performance bias: +<br>Attrition bias: +<br>Detection bias: ?<br>**Authors' conclusion**<br>"In the present study of trauma patients, prehospital use of TXA was associated with prolonged time to death and significantly improved early survival, suggesting benefits of TXA on haemostatic resuscitation. Until further evidence emerges, the results support the use of TXA during prehospital treatment of severely injured patients."<br>**Reviewers' conclusion**<br>Precautions were taken to minimise biases. The intervention and control groups were comparable with respect to both baseline characteristics and co-interventions.<br>It is unclear how outcomes with missing values were determined.

Fibrinogen

Study: Reference, aim, design, setting	Participants: selection criteria, characteristics	N Participants; Intervention (IG) vs. Control group (CG)	Main outcomes	Assessment: LoE, risk of bias; Conclusions
**Ziegler (2021)**<br>"Efficacy of prehospital administration of fibrinogen concentrate in trauma patients bleeding or presumed to bleed (FIinTIC): A multicentre, double-blind, placebo-controlled, randomised pilot study". *European Journal of Anaesthesiology* 38(4): 348.<br>**Study design**<br>Randomised controlled trial<br>**Aim of the study**<br>The aim of the study was to administer fibrinogen concentrate in the prehospital setting to improve blood clot stability in trauma patients bleeding or presumed to bleed.<br>**Setting**<br>Austria, Germany, Czech Republic, 2011-2015	**Inclusion criteria**<br>• Trauma patients<br>• ≥18 years of either sex<br>• Major bleeding or occult bleeding<br>• Need for volume replacement therapy<br>• Patient admitted to one of the participating hospitals<br>**Exclusion criteria**<br>• Solely penetrating trauma<br>• Solely head injury<br>• In case of ongoing severe hemodynamic instability refractory to therapy (vasopressor, volume)<br>• Patient with inevitable lethal course as evaluated by emergency physician<br>• Need for CPR on the scene<br>• Deep hypothermia (<30°C)<br>• Obviously pregnant women<br>• Patient with known recent history of thromboembolic events within the last 6 months<br>• Patient known to be on anticoagulant therapy<br>• Patient with known refusal of a participation in this clinical trial<br>**Characteristics**<br><u>Age [y], median (IQR)</u><br>IG: 46 (34.5-58) vs. CG: 54 (37-56), p=0.4868<br><u>Sex Male, n (%)</u>	**Participants**<br>N=53 patients<br>**Study groups**<br>IG: fibrinogen concentrate 1.5 g per 30 kg of estimated bodyweight (i.v., 20ml min-1) (N=37)<br>CG: placebo (N=30)<br>**Participants analysed/excluded from analysis**<br><u>IG analysed (N=28)</u><br>Excluded from analysis (*N*=9)<br>• Transfer to non-study hospital (*N*=1)<br>• Anticoagulant treatment (*N*=1)<br>• Inevitably lethal course (*N*=2)<br>• Primary endpoint missing (*N*=5)<br><u>CG analysed (N=25)</u><br>Excluded from analysis (*N*=5)<br>• Transfer to non-study hospital (*N*=1)<br>• Age <18 years (*N*=1)<br>• Inevitably lethal course (*N*=1)<br>• Primary endpoint missing (*N*=2)	<u>FIBTEM maximum clot firmness (FIBTEM MCF) at emergency room admission, median difference (IQR)</u><br>CG vs. IG: -4, (-7 to -2), p<0.0026<br><u>FIBTEM maximum clot firmness (FIBTEM MCF) change from baseline to emergency room admission, median difference (IQR)</u><br>CG vs. IG: -5 (-7 to -3), p<0.0001<br><u>EXTEM clotting time (EXTEM CT) at emergency room admission, median difference (IQR)</u><br>CG vs. IG: 0 (-5 to 5), p<0.9858<br><u>EXTEM clotting time (EXTEM CT) change from baseline to emergency room admission, median difference (IQR)</u><br>CG vs. IG: 6.8 (0 to 12), p<0.0509<br><u>EXTEM maximum clot firmness (EXTEM MCF) at emergency room admission, median difference (IQR)</u><br>CG vs. IG: -5 (-9 to -2), p<0.0102<br><u>EXTEM maximum clot firmness (EXTEM MCF) change from baseline to emergency room admission, median difference (IQR)</u><br>CG vs. IG: -5 (-8 to -2), p<0.0031<br>**Changes in FIBTEM maximum clot firmness (FIBTEM MCF) between baseline (T1) and 7 days posttrauma (T7)**<br><u>T2: on arrival at the ED</u>	**Level of evidence**<br>2b↓<br>**Risk of bias**<br>Selection bias: +<br>Performance bias: +<br>Attrition bias: –<br>Detection bias: +<br>**Authors' conclusion**<br>"Early fibrinogen concentrate administration is feasible in the complex and time-sensitive environment of prehospital trauma care. It protects against early fibrinogen depletion, and promotes rapid blood clot initiation and clot stability."<br>**Reviewers' conclusion**<br>There is a risk of attrition bias as the primary endpoint is missing for more patients in the IG than then CG.<br>The outcomes are surrogate outcomes; mortality, morbidity or

Study: Reference, aim, design, setting	Participants: selection criteria, characteristics	N Participants; Intervention (IG) vs. Control group (CG)	Main outcomes	Assessment: LoE, risk of bias; Conclusions
	IG: 23/28 (82.1) vs. CG: 21/25 (84.0), p=1<br><u>ISS, median (IQR)</u><br>IG: 25 (16-36) vs. CG: 16 (16-34), p=0.393<br><u>GCS, median (IQR)</u><br>IG: 14.5 (6-15] vs. CG: 15 (13-15), p=0.574		CG vs. IG:–4 (–7 to –2), p<0.0026<br><u>T3: 3 h after ED admission</u><br>CG vs. IG:–3 (–6 to 0), p<0.0851<br><u>T4: 9 h after ED admission</u><br>CG vs. IG: -2 (-5 to 1), p<0.1812<br><u>T5: 24 h after ED admission</u><br>CG vs. IG: -2 (-5 to 1), p<0.102<br><u>T6: 48 h after ED admission</u><br>CG vs. IG: -1 (-4 to 2), p<0.7328<br><u>T7: 7 days after ED admission</u><br>CG vs. IG: -2 (-8 to 3), p<0.5084	transfusion requirements were not investigated.

Intraosseous access

Study: Reference, aim, design, setting	Participants: selection criteria, characteristics	N Participants; Intervention (IG) vs. Control group (CG)	Main outcomes	Assessment: LoE, risk of bias; Conclusions
**Chreiman (2018)**<br>"The IOs have it: a prospective observational study of vascular access success rates in patients in extremis using video review." *The Journal of Trauma and Acute Care Surgery* 84(4): 558-563.<br>**Study design**<br>Retrospective cohort study<br>(study included because	**Inclusion criteria**<br>• Hypovolemic trauma patients<br>• Presenting to the hospital in extremis (absence of a palpable pulse or measureable blood pressure)<br>• Vascular access: intraosseous (IO) (including tibial and humoral), peripheral IV (PIV), central venous catheter (CVC) (including internal jugular, subclavian, and femoral line) or intracardiac line (IC)<br>**Characteristics**	**Participants**<br>N=38 patients, 145 vascular access attempts<br>**Study groups**<br>IG: intraosseous access (52 attempts)<br>CG 1: peripheral intravenous access (37 attempts)<br>CG 2: central venous catheter access (52 attempts)	**Primary endpoint**<br><u>Success rates by type of vascular access attempt: n/N (%)</u><br>IG 48/52 (92) [tibial 38/38 (100), 2 with missing data; humeral 10/12 (83.3)]<br>vs. CG 1: 12/37 (43.2)<br>vs. CG 2: 23/52 (44.2) [Femoral: 11/ 24 (45.8), Subclavian: 11/24 (45.8), Internal Jugular 1/2 (50)]<br>vs. CG 3: 3/4 (75)<br>p<0.001<br>Success rates were not different between sites by access type (p=0.54 for tibia vs. humeral IO;	**Level of evidence**<br>3b↓<br>**Risk of bias**<br>Selection bias: –<br>Performance bias: ?<br>Attrition bias: ?<br>Detection bias: ?<br>**Authors' conclusion**<br>"Access attempts using IO are as fast as PIV attempts

Study: Reference, aim, design, setting	Participants: selection criteria, characteristics	N Participants; Intervention (IG) vs. Control group (CG)	Main outcomes	Assessment: LoE, risk of bias; Conclusions
"The use of audiovisual recordings allowed us to collect data in a fashion similar to prospective real-time data collection […].")<br><br>**Aim of the study**<br>With the goal of informing guidelines for vascular access in hypovolemic trauma patients, we sought to study the real-time provision of vascular access in patients presenting to the hospital in extremis, defined for the purpose of this study as the absence of a palpable pulse or measurable blood pressure.<br><br>**Setting**<br>USA, 2016-2017	<u>Male gender n (%)</u><br>35 (92)<br><br><u>Age [y], median (IQR)</u><br>30 (25-38)<br><br><u>Injury Mechanism, n (%)</u><br>Gunshot wound 31 (82%)<br>Stab wound 4 (10%)<br>Other 3 (8%)<br><br><u>ISS, median (IQR)</u><br>25 (16-25)<br><br>All patients undergoing Emergency Department Thoracotomy.	CG 3: intracardiac line (4 attempts)	p=0.99 for femoral vs. subclavian vs. internal jugular CVC)<br><br>**Secondary endpoint**<br><u>The time to completion of access attempts by type of vascular access, minutes: median (IQR)</u><br>IG: 0.39 (0.13-0.65)<br>vs.CG 1: 0.63 (0.35-0.96), adjusted p=0.03<br><br>Both IG and CG1 were faster than CG 2 [3.2 (1.72 – 5.23)], adjusted p<0.001	but are more than twice as likely to be successful. Attempts at CVC access in patients in extremis have high rates of failure and take a median of over 3 minutes. While IO access may not completely supplant PIVs and CVCs, IO access should be considered as a first line therapy for trauma patients in extremis."<br><br>**Reviewers' conclusion**<br>Due to insufficient reporting it is unclear whether the groups are comparable. There may be a high risk of selection bias, and results need to be interpreted with caution.
**Leidel (2012)**<br>"Comparison of intraosseous versus central venous vascular access in adults under resuscitation in the emergency department with inaccessible peripheral veins".	**Inclusion criteria**<br>• adults<br>• all severely injured or critically ill patients under resuscitation<br>• admitted to ED without at least 1 efficient 18-gauge peripheral IV access<br>• indications for vascular access included blood drawing for serum analysis, delivery of drugs, antibiotics, fluids or	**Participants**<br>N=40 patients<br><br>**Study groups**<br>IG: intraosseous (IO) access (N=40) primarily proximal humerus<br><br>CG: central venous catheter (CVC) (N=40) primarily subclavian vein	<u>Success rate on first attempt, n/N, % (95% CI)</u><br>IG: 34/40, 85 (74 to 96)<br>CG: 24/40, 60 (45 to 75), p=0.024<br><br><u>Procedure time [min], median (IQR)</u><br>IG: 2.0 (1.0 to 3.0)<br>CG: 8.0 (5.5 to 10.0), p<0.001<br><br><u>Unsuccessful attempts n/N</u>	**Level of evidence**<br>3b↓<br><br>**Risk of bias**<br>Selection bias: +<br>Performance bias: –<br>Attrition bias: +<br>Detection bias: +

Study: Reference, aim, design, setting	Participants: selection criteria, characteristics	N Participants; Intervention (IG) vs. Control group (CG)	Main outcomes	Assessment: LoE, risk of bias; Conclusions
Resuscitation 2012, 83: 40-45.<br><br>**Study design**<br>Prospective cohort study<br><br>**Aim of the study**<br>To compare the time required to establish IO access versus CVC in adult patients undergoing resuscitation who initially had unsuccessful attempts at peripheral IV access, as well as report on their complication rates.<br><br>**Setting**<br>Germany, 2007-2009	blood products when no other access was available.<br><br>**Exclusion criteria**<br>• age <18 years<br>• pregnancy<br>• prisoners<br><br>**Characteristics**<br><br><u>Gender male, n/N (%)</u><br>27/40 (68)<br><br><u>Age [y], mean ± SD (range)</u><br>48 ± 21 (18-87)<br><br><u>Obesity, BMI >30 kg/m², n/N (%)</u><br>7/40 (18)<br><br><u>Trauma, n/N (%) *CAVE!*</u><br>29/40 (73)	(each patient received both IO and CVC, site depending on injury pattern)<br><br>During initial resuscitation, peripheral IV access was attempted for ≤3 efforts or ≤2 min. If unsuccessful, IO access and CVC were performed in a standardised course of action by 2 independent operators.<br><br>**IO devices**<br>• battery driven EZ-IO system (Vidacare Corp.)<br>• spring load driven Adult BIG Bone Injection Gun (WaisMed Ltd.)	IG: 6/40<br>CG: 16/40<br><br><u>Reasons for unsuccessful attempts</u><br>IG: cannula did not penetrate the bone cortex due to incorrect tibial insertion site (n=4) (BIG Bone Injection Gun); excessive humeral overlying soft tissue (n=2) (EZ-IO)<br><br>CG: inability to insert or advance the guide wire into the vessel probably due to incorrect insertion site or technique	**Authors' conclusion**<br>"We found IO vascular access a safe, reliable and rapid option in adults under resuscitation in the emergency department with inaccessible peripheral veins. Compared to landmark-based CVC, IO cannulation was significantly more successful on first attempt and required significantly less time."<br><br>**Reviewers' conclusion**<br>The study was well conducted and reported. Study subjects were the same in both arms, and the study was sufficiently powered. Due to lack of blinding, there is a residual risk of performance bias.<br><br>The relevance of the results for the severe trauma population is limited by the fact that a substantial fraction of the population (27%) were non-trauma patients, leading to downgrading.
+: low risk; –: high risk; ?: unclear risk; CI: Confidence Interval; HR: Hazard Ratio; IQR: Interquartile Range; ITT: Intention to Treat; OR: Odds Ratio; RR: Relative Risk; SD: Standard Deviation; SEM: Standard Error of Mean				

Table S5. Deleted Recommendations

Number	Recommendation (German)	Reason
1.24 A	Isotone Kochsalzlösung soll nicht verwendet werden.	Expert consensus
1.27 A	Humanalbumin soll nicht zur präklinischen Volumentherapie herangezogen werden.	Expert consensus
1.28 0	Beim polytraumatisierten Patienten nach stumpfem Trauma mit hypotonen Kreislaufverhältnissen können hypertone Lösungen verwendet werden.	Expert consensus
1.29 0	Bei penetrierendem Trauma können hypertone Lösungen verwendet werden, sofern hier eine präklinische Volumentherapie durchgeführt wird.	Expert consensus
